# Computational analysis of LexA regulons in Cyanobacteria

**DOI:** 10.1186/1471-2164-11-527

**Published:** 2010-09-29

**Authors:** Shan Li, Minli Xu, Zhengchang Su

**Affiliations:** 1 Bioinformatics Research Center, Department of Bioinformatics and Genomics, the University of North Carolina at Charlotte, Bioinformatics Building 351, 9201 University City Blvd., Charlotte, NC 28223, USA

## Abstract

**Background:**

The transcription factor LexA plays an important role in the SOS response in *Escherichia coli *and many other bacterial species studied. Although the *lexA *gene is encoded in almost every bacterial group with a wide range of evolutionary distances, its precise functions in each group/species are largely unknown. More recently, it has been shown that *lexA *genes in two cyanobacterial genomes *Nostoc sp*. PCC 7120 and *Synechocystis sp*. PCC 6803 might have distinct functions other than the regulation of the SOS response. To gain a general understanding of the functions of LexA and its evolution in cyanobacteria, we conducted the current study.

**Results:**

Our analysis indicates that six of 33 sequenced cyanobacterial genomes do not harbor a *lexA *gene although they all encode the key SOS response genes, suggesting that LexA is not an indispensable transcription factor in these cyanobacteria, and that their SOS responses might be regulated by different mechanisms. Our phylogenetic analysis suggests that *lexA *was lost during the course of evolution in these six cyanobacterial genomes. For the 26 cyanobacterial genomes that encode a *lexA *gene, we have predicted their LexA-binding sites and regulons using an efficient binding site/regulon prediction algorithm that we developed previously. Our results show that LexA in most of these 26 genomes might still function as the transcriptional regulator of the SOS response genes as seen in *E. coli *and other organisms. Interestingly, putative LexA-binding sites were also found in some genomes for some key genes involved in a variety of other biological processes including photosynthesis, drug resistance, etc., suggesting that there is crosstalk between the SOS response and these biological processes. In particular, LexA in both *Synechocystis sp. *PCC6803 and *Gloeobacter violaceus *PCC7421 has largely diverged from those in other cyanobacteria in the sequence level. It is likely that LexA is no longer a regulator of the SOS response in *Synechocystis sp*. PCC6803.

**Conclusions:**

In most cyanobacterial genomes that we analyzed, LexA appears to function as the transcriptional regulator of the key SOS response genes. There are possible couplings between the SOS response and other biological processes. In some cyanobacteria, LexA has adapted distinct functions, and might no longer be a regulator of the SOS response system. In some other cyanobacteria, *lexA *appears to have been lost during the course of evolution. The loss of *lexA *in these genomes might lead to the degradation of its binding sites.

## Background

The LexA protein was first characterized as the transcriptional regulator of the SOS response in *Escherichia coli *[1,2], and later in several other bacteria, including *Bacillus subtilis *[3,4] and *Fibrobacter succinogenes *[5]. In fact, the *lexA *gene is found in almost all eubacterial groups examined so far [5,6]. In *E. coli*, around 30 genes involved in the SOS response are under the regulation of LexA [2]. Under normal growth conditions, LexA represses the SOS response genes by binding to their promoter regions, and thus blocking their transcription. When DNA is damaged, the binding of RecA to the released single-stranded DNA induces the auto-cleavage of the Ala^84^-Gly^85 ^peptide bond [7,8] in LexA, thereby inhibiting the dimerization of LexA and preventing its binding to DNA [9-11]. In this manner, SOS response genes are de-repressed and expressed at different time points and different levels in a coordinated way [10].

LexA in *E. coli *consists of an N-terminal DNA-binding domain and a C-terminal dimerization domain [8,12]. The N-terminal contains three α-helices (I, II, III) and an anti-parallel β-sheet [12]. Helices II and III form a helix-turn-helix DNA-binding motif, and all the DNA-contacting residues Ser^39^, Asn^41^, Ala^42^, Glu^44 ^and Glu^45 ^are located in helix III [13] as revealed by both NMR [12] and X-Ray crystallography analyses [8]. The LexA-binding sites in *E. coli *were found to be a 16-bp palindromic motif with the consensus sequence CTG(TA)_5_CAG [14]. It has been shown that two reactive residues Ser^119 ^and Lys^156 ^in *E. coli *LexA are critical for the auto-hydrolysis of the peptide bond Ala^84^-Gly^85 ^[1,9]. The core set of the SOS response system consists of *lexA, recA, uvrABCD, umuCD *and *ruvB *[10]. Upon the auto-hydrolysis of LexA, the *uvrABCD *operon is expressed first, whose products are responsible for the nucleotide excision repair (NER). Then *recA *and several other genes for homologous recombination are expressed, retrieving the excised DNA double strands. Next, the cell division inhibitor SfiA is induced to guarantee a sufficient time for the DNA repairing to be completed. In the end, if the DNA is not completely repaired, the operon *umuCD *encoding the mutagenic DNA repair polymerase Pol V will be induced to perform translesion DNA synthesis [9,14]. Since the *lexA *gene itself is also under the control of LexA, after the damaged DNA is repaired, the activity of RecA declines, the production of LexA surpasses its auto-cleavage. Consequently, the increased concentration of LexA restores the inhibition of the expression of the SOS response genes.

More recently, LexA homologs were also experimentally studied in a few cyanobacteria [15-21]. These studies suggest that LexA in *Nostoc sp*. PCC 7120 [16] binds to the promoter regions of *lexA *and *recA*; however, LexA in *Synechocystis sp*. PCC 6803 may regulate different genes/systems other than the SOS system. Domain *et al*. concluded from microarray gene profiling analysis [21] that LexA in this species might be involved in carbon metabolism. Later, LexA in *Synechocystis sp*. PCC 6803 was found to regulate the *crhR *gene encoding a RNA helicase [19]. Moreover, it has been shown that the transcription of the bidirectional hydrogenase genes *hoxEFUYH *was regulated by LexA in *Synechocystis sp*. PCC6803[17]. In *Nostoc sp*. PCC 7120, *hoxEFUYH *genes are split into two separate operons, and LexA was found to bind to the upstream regions for both operons [15]. Mazon *et al. *[16] showed that the LexA-binding sites in *Nostoc sp*. PCC 7120 have a 14-bp pseudo-palindromic structure in the form of RGTACNNNDGTWCB, which are similar to those in *B. subtilis*. Additionally, Sjöholm *et al*. [15] found two putative palindromic LexA-binding sites: one in the promoter region of *alr0750-hoxE-hoxF *that resembles Mazon's LexA boxes[16], and another, TTACACTTTAA in the upstream region of *hoxU *in *Nostoc sp*. PCC 7120. Meanwhile, multiple putative LexA boxes were identified in *Synechocystis sp*. PCC6803: a 13-bp pseudo-palindromic segment AGTAACTAGTTCG in the upstream region of *hoxE*, which is similar to Mazon's site but with one base deletion [17]; another direct repeat pattern, CTA-N_9_-CTA proposed to be recognized by LexA in vitro [20]; and two putative LexA boxes that resemble none of the putative LexA boxes listed above [18]. Despite this progress, a more extensive study of LexA proteins and their binding sites and regulons in cyanobacterial genomes is still needed. In this study, we have predicted LexA-binding sites and regulons in all the sequenced cyanobacterial genomes that harbor a *lexA *gene, and analyzed the evolutionary changes in the LexA regulons in cyanobacteria, as well as their relationship with those in proteobacteria and gram-positive bacteria.

## Results and Discussion

### Conservation of the DNA-binding domain of LexA in cyanobacteria

**Figure 1:**
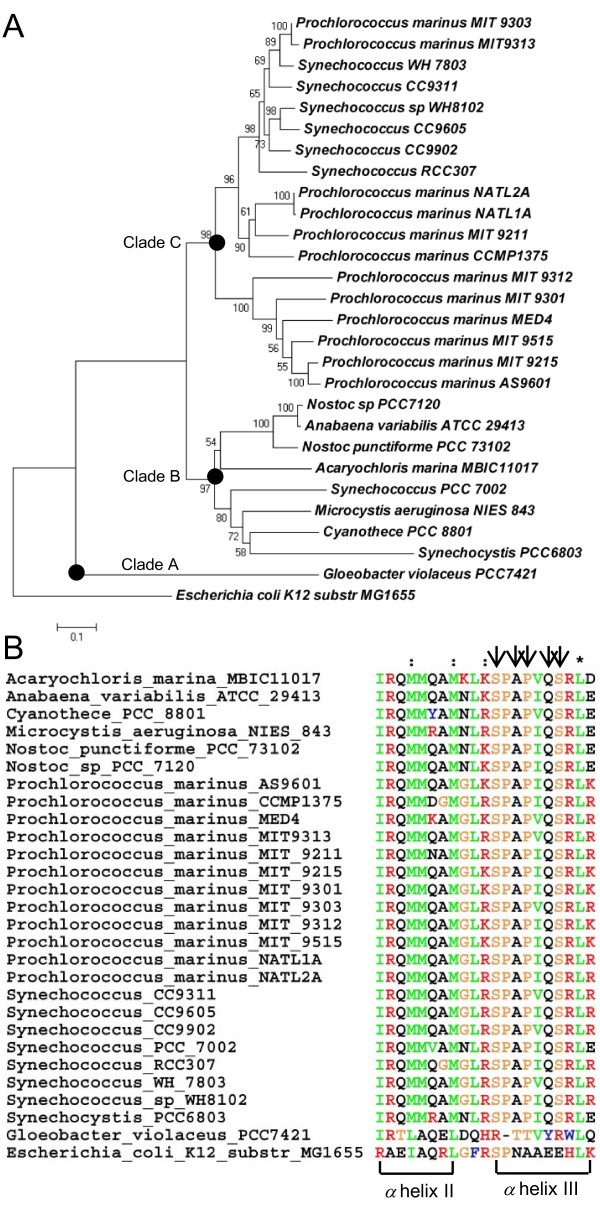
**Phylogenetic relationships of 27 cyanobacterial LexA proteins and their DNA-binding domains**. (A) Phylogenetic relationships of the 27 cyanobacterial LexA proteins. The tree is rooted with the LexA in *E. coli *K12. Bootstrap values are shown on the nodes. (B) Alignment of the DNA-binding domain (DBD) of the 27 cyanobacterial LexA proteins. The DBD of LexA contains a helix-turn-helix motif, and DNA-contacting residues are located in helix III, and are labelled by vertical arrows.

We identified orthologs of the LexA protein in *Nostoc sp*. PCC7120 (alr4908) in 26 of the 33 sequenced cyanobacterial genomes using the bi-directional best hit (BDBH) method based on BLASTP search with an *E*-value cutoff 10^-10 ^(see Methods). Seven genomes appear not to harbor a *lexA *gene under this criterion, namely, *Gloeobacter violaceus *PCC7421, *Synechococcus sp. *JA-3-3Ab A-Prime, *Synechococcus sp. *JA-2-3B'a(2-13) B-Prime, *Synechococcus elongatus *PCC6301, *Synechococcus elongatus *PCC7942, *Trichodesmium erythraeum *IMS101 and *Thermosynechococcus elongatus *BP-1. We removed the *Synechococcus elongatus *PCC7942 genome from our study since *Synechococcus elongatus *PCC6301 is virtually identical to it [22]. However, an ortholog of the *lexA *gene (Gll0709) does exist in *Gloeobacter violaceus *PCC7421. The reason we failed to identify this ortholog is that it does not meet our BDBH criterion due to its largely divergent sequence. The phylogenetic tree of these 27 LexA amino acid sequences indicates that they can be clustered into three groups (Figure 1A), corresponding to the previously described Clade A (containing *Gloeobacter violaceus *PCC7421), Clade C (containing small marine *Prochlorococcus *and *Synechococcus*), and Clade B (containing most remaining cyanobacteria) [23,24]. However, aside from *Gloeobacter violaceus *PCC7421, the DNA-binding domains (DBD) of LexA from these cyanobacteria are highly conserved (Figure 1B), especially the helix III, where DNA-contacting residues are located [13]. This result is in agreement with earlier observations [16,21]. This provides the rationale of our analysis, including the phylogenetic footprinting analysis (next section) and genome-wide scanning for LexA-binding site predictions. On the other hand, since the DBD in *Gloeobacter violaceus *PCC7421 is quite different from those in other cyanobacteria, especially where the DNA-contacting residues locate, thus, we excluded it from our study, leaving 31 species/strains for the putative LexA regulon prediction.

### LexA-binding sites predicted by phylogenetic footprinting

We considered both an operon and a singleton gene as a transcription unit (TU). As Mazon *et al. *[16] have demonstrated the binding of LexA to the upstream regions of two genes, *lexA *and *recA*, and predicted LexA-binding sites for other four genes (*uvrA*, *ssb*, *alr4905*, and *all4790*) in *Nostoc sp *PCC7120, we used phylogenetic footprinting to identify possible LexA-binding sites in the pooled 118 inter-TU sequences associated with these six genes in *Nostoc sp*. PCC7120 [16] and their orthologs in the other 25 cyanobacterial genomes (excluding *Gloeobacter violaceus *PCC7421) that harbor a *lexA *gene (see Methods). We identified 49 high-scoring 14-bp palindromic sequences (Table 1) out of the 118 input sequences by applying the motif finding tools MEME [25] and BioProspector [26] and incorporating the best motifs found by these two programs (See Methods and Additional file 1). However, the putative LexA box AGTCCTAGAGTCCT (Additional file 1) identified in *Synechocystis sp*. PCC6803 was not identified by Patterson-Fortin *et al*. [20] using DNaseI footprinting assays or by Gutekunst *et al. *[17]. Therefore, we removed this site, leaving 48 putative LexA-binding sites (Table 1) for profile construction. The two LexA boxes that have been characterized in *Nostoc sp *PCC 7120 [16] were accurately recovered by the phylogenetic footprinting procedure (Table 1), suggesting that most of these high-scoring motifs are likely to be genuine LexA boxes. These putative LexA-binding sites show either a strong palindromic structure similar to the experimentally characterized LexA boxes in *Nostoc sp*. PCC7120 [16], or a tandem repeat structure with the consensus sequence AGTACWNWTGTACT. As demonstrated in Figure S1 in Additional file 2, this pattern is rather similar to the consensus sequence of the LexA-binding sites previously identified in *B. subitlis *(CGAACN_4_GTTCG) [3], and to a less extent, to that of LexA-binding sites found in α-proteobacteria (GTTCN_7_GTTC and GAACN_7_GAAC) [27], but differs remarkably from that in *E. coli *CTG(TA)_5_CAG [14]. These results are consistent with our phylogenetic analysis of the 183 LexA proteins detected in 598 genomes, showing that LexA proteins in cyanobacteria are more closely related to those in gram-positive and α-proteobacteria bacteria than to those in γ-proteobacteria (Figure S2 in Additional file 3). Accordingly, since the LexA-binding sites in *B. subtilis *[3,16] have a palindromic structure, it is not surprising that the LexA-binding sites in cyanobacterial genomes might have a similar palindromic structure.

**Table 1:** 48 Putative LexA binding sites identified by phylogenetic footprinting analysis

Genome	Transcription Unit	Name	Putative LexA-binding sites	Position^1^
Acaryochloris marina MBIC11017	*AM1_3549 AM1_3550*	*- recA*	**AATAAATCTGTACT**	-97
	*AM1_3948*	*lexA*	**AGTACAGGTGTTTT**	-132
Anabaena variabilis ATCC 29413	*Ava_2176*	-	**AGTTCTCATGTACT**	-144
	*Ava_1462*	-	**AGTACTTATGTACT**	-56
	*Ava_3591*	-	**AGTTCTTCTGTATC**	-112
	*Ava_2198*	*lexA*	**AGTACTAATGTTCT**	-47
	*Ava_2059 Ava_2058*	*- -*	**CGTACATTTGTACC**	-71
	*Ava_4925*	*recA*	**AGTATATCTGTTCT**	-93
Cyanothece PCC 8801	*PCC8801_0945*	-	**AAAACTCTTGTACT**	-78
	*PCC8801_2186 PCC8801_2185*	*- -*	**AGTACTTATGTTCG**	-101
Microcystis aeruginosa NIES 843	*MAE_39060*	*ssb*	**CATACTATTGTACT**	-59
	*MAE_16070*	*recA*	**CATACTGCTGTACT**	-68
Nostoc punctiforme PCC 73102	*Npun_F1842*	-	**AGTACACCTGTACT**	-56
	*Npun_F2914*	*recA*	**AGTATATCTGTTCT**	-102
	*Npun_F6100 Npun_F6101 Npun_F6102*	*- - -*	**AGTACGATTGTTCT**	-111
	*Npun_R5568 Npun_R5567*	*- -*	**CGTACATTTGTACT**	-74
Nostoc sp PCC7120	*alr4908*	*lexA*	**AGTACTAATGTTCT**	-35
	*all4790 all4789*	*- -*	**CGTACATTTGTACC**	-31
	*alr4905*	-	**AGTTCTCATGTACT**	-100
	*alr3716*	*uvrA*	**AGTACTATTGTTCT**	-72
	*alr0088*	*ssb*	**AGTACTTATGTACT**	-16
	*all3272*	*recA*	**AGTATATCTGTTCT**	-52
Prochlorococcus marinus AS9601	*A9601_17691*	*recA*	**AGTACAGATGTACT**	-126
Prochlorococcus marinus CCMP1375	*Pro1784*	*ssb*	**AAAACATAAGTATT**	-109
Prochlorococcus marinus MED4	*PMM1562*	*recA*	**AGTACACATGTACT**	-123
	*PMM1262*	*lexA*	**GGTACAAATGTATT**	-57
Prochlorococcus marinus MIT9313	*PMT0380*	-	**GGTACACATGTATT**	-56
Prochlorococcus marinus MIT9211	*P9211_13051 P9211_13041*	*lexA -*	**GGTACATATGTATT**	-69
Prochlorococcus marinus MIT9215	*P9215_18341*	*recA*	**AGTACAGATGTACT**	-126
Prochlorococcus marinus MIT9301	*P9301_17531*	*recA*	**AGTACAGATGTACT**	-125
Prochlorococcus marinus MIT9303	*P9303_19141*	*lexA*	**GGTACACATGTATT**	-81
Prochlorococcus marinus MIT9312	*PMT9312_1654*	*recA*	**AGTACAGATGTACT**	-126
Prochlorococcus marinus MIT9515	*P9515_17441*	*recA*	**AGTACGCATGTACT**	-123
	*P9515_18121*	-	**AATATATCTATTCT**	-139
Prochlorococcus marinus NATL1A	*NATL1_20071*	*recA*	**CGTACGTCTGTACT**	-132
	*NATL1_16801*	*lexA*	**AGGACAAATGTACT**	-52
Prochlorococcus marinus NATL2A	*PMN2A_1133*	*recA*	**CGTACGTCTGTACT**	-132
	*PMN2A_0828*	*lexA*	**AGGACGAATGTACT**	-52
Synechococcus CC9605	*Syncc9605_0929*	*lexA*	**GGTACAAATGTATT**	-61
	*Syncc9605_0104*	-	**GATACCGCAGTTTA**	-140
Synechococcus CC9902	*Syncc9902_1949*	*recA*	**CGTACGTTTGTACT**	-104
	*Syncc9902_1481*	*lexA*	**GGTACAAATGTATT**	-59
Synechococcus PCC7002	*SYNPCC7002_A0426 SYNPCC7002_A0425 SYNPCC7002_A0424*	*recA - -*	**AGTACGATTGAACT**	-90
	*SYNPCC7002_A0119*	*ssb*	**AGAACAGTTGTATG**	-53
Synechococcus RCC307	*SynRCC307_1756*	*lexA*	**GGCACAAATGTATT**	-39
Synechococcus WH7803	*SynWH7803_0171*	*ssb*	**CAACCGTCAGTTCT**	-56
	*SynWH7803_0439*	*recA*	**CGTACATCTGTACT**	-172
Synechococcus sp WH8102	*SYNW2062*	*recA*	**CGTACGCCTGTACT**	-104

1. Positions of the LexA binding sites relative to the first codon of the operon.

### Genome-wide prediction of LexA-binding sites and regulons in cyanobacterial genomes

Both consensus sequence and position weight matrix (PWM) have been widely used to represent the pattern of similar sequences. The advantage of PWM (or profile) methods over the consensus sequence methods is that the former can capture more quantitative information about the patterns by using a probabilistic model to represent the sequences. In this way, it can differentiate subtly conserved positions from the non-conserved ones [28]. In our study, We used the profile of these 48 LexA boxes (Table 1) to scan the 31 sequenced cyanobacterial genomes to predict additional putative LexA-binding sites and members of LexA regulons, using a scanning algorithm [29-31] that incorporates orthologous information and computes a log-odds ratio (*LOR*) score for evaluating the confidence of predictions in each genome (see Methods for details). The predicted results with a p-value < 0.01 for the 26 genomes harboring a *lexA *gene are listed in Table S1-26 (Additional file 4), while those for the five genomes without a *lexA *gene are listed in Table S27-31 (Additional file 5). The predicted results with a *p*-value < 0.05 for the 31 cyanobacteria are summarized in Table S32-62 (Additional file 6).

The score of a detected putative LexA binding site for a TU is the sum of two terms: one evaluates the extent to which the putative LexA binding site resembles the scanning profile; the other evaluates the similarity of this binding site to those identified for the orthologs of genes within the TU in the other genomes. To evaluate the confidence of each motif score *s*, we used randomly selected coding sequences as the null model to test the statistical significance. A false positive rate was used to evaluate this statistical significance, which was defined as the fraction of the randomly selected coding sequences containing binding sites with a score higher than the cutoff *s *in the genome. We chose randomly selected coding regions as the null model based on the assumption that a coding sequence is less likely to contain *cis*-regulatory binding sites than an intergenic sequence. Although it might be possible for genuine LexA boxes to occur in coding regions [15,20], such kind of binding sites should be rare. The *LOR *function for a genome evaluates the ratio of the fraction of the inter-TU sequences containing a binding site with a score higher than *s *to the fraction of the randomly selected coding sequences containing a binding site with a score higher than the same *s *in the genome. Accordingly, positive *LOR *values that increase monotonically with the increase in binding site sores would suggest that an inter-TU sequence is more likely to contain a high-scoring LexA-binding site than does a randomly selected coding sequence in the genome.

**Figure 2:**
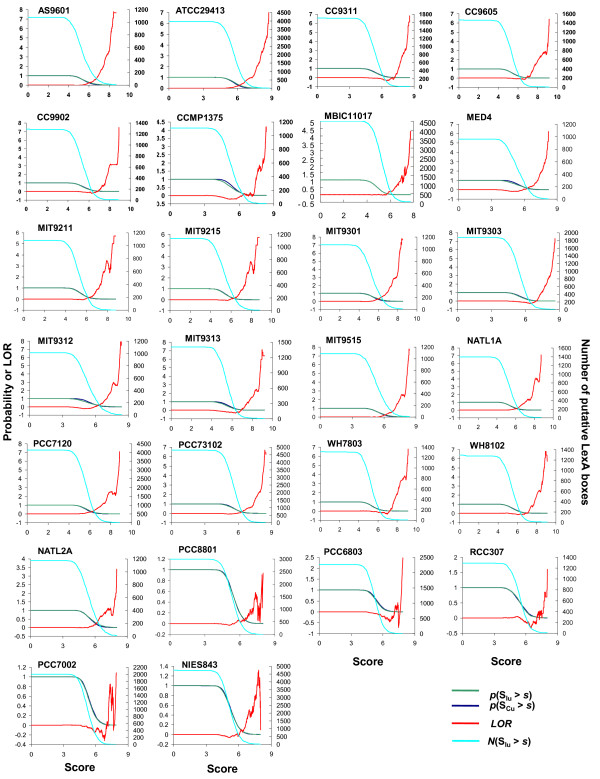
**Evaluation of the predictions of LexA-binding sites in the 26 cyanobacterial genomes**. The green curves represent the probability $p(S_{I_{U}}>s)$ and the blue curves $p(S_{C_{U}}>s)$. The cyan curves are the number of iner-TU regions containing a putative binding site with a score > s, $N(S_{I_{U}}>s)$. The red curves are the log-odds ratio (*LOR*), defined as $LOR(s)=\ln(p(S_{I_{U}}>s)/p(S_{C_{U}}>s))$, (see Methods). Refer to Abbreviation for the full name of each genome.

As shown in Figure 2, when the motif score *s *increases beyond some value, the *LOR *is generally high for most of the 26 cyanobacteria that harbor a *lexA *gene, therefore those genomes with high *LOR *values are likely to contain some true binding sites. Exceptions exist in five genomes, namely, *Cyanothece sp*. PCC 8801, *Synechocystis sp. *PCC6803, *Synechococcus *RCC307, *Synechococcus sp*. PCC 7002, and *Microcystis aeruginosa *NIES-843, in which the *LOR *curves oscillate around zero when binding site score *s *increases. These poor *LOR *values might suggest that there are not more high-scoring LexA-binding sites in the inter-TU regions than in the coding regions in the five genomes. The reason for this could be that our scanning algorithm rewards a binding site that is shared by orthologs in the other genomes. If a true binding site is unique to a genome, then it will not score high. In this sense, LexA is probably no longer a major SOS response regulator in these genomes. Instead, it might have become a specific local regulator during the course of evolution to adapt to their unique living environments (we will return to this subject later). In the case of *Synechocystis sp. PCC6803*, it is noted that the LexA-binding sites identified by Patterson-Fortin *et al. *[20] are totally different from those identified by Mazon *et al*. [16], and that the LexA sequence in this genome is largely divergent from those in the other genomes (Figure 1A). Accordingly, the LexA binding sites in this genome might differ in some way from those in the other genomes, which can be another reason for its low *LOR *values.

In contrast, as shown in Figure S3 in Additional file 2, the *LOR *values in the five genomes that do not harbor a *lexA *gene (*Synechococcus sp. *JA-3-3Ab A-Prime, *Synechococcus sp*. JA-2-3B'a (2-13) B-Prime, *Synechococcus elongatus *PCC 6301, *Thermosynechococcus elongates *BP-1 and *Trichodesmium erythraeum *IMS101) oscillate around or decrease below zero when the motif score *s *increases beyond a certain value, implying that the chance to find a relative high-scoring putative LexA-binding site in an inter-TU region is not higher than in a randomly chosen coding sequence, suggesting that these genomes are unlikely to contain functional LexA-binding sites. On the other hand, the *LOR *values in the three genomes *Synechococcus sp. *JA-3-3Ab A-Prime, *Synechococcus sp*. JA-2-3B'a (2-13) B-Prime and *Trichodesmium erythraeum *IMS101 are relatively higher than those in the other two genomes (Additional file 2, Figure S3), or even could be comparable to those of the five poor-LOR-valued cyanobacteria that harbor a *lexA *gene (Figure 2). In fact, the numbers of predicted binding sites in the three genomes are not too small (Table S27, S28, S31 in Additional file 5), which suggests that a few putative *LexA*-like binding sites exist in these genomes. A possible explanation for this phenomenon could be that these LexA-like binding sites are recognized by other transcription factors that have similar DNA-binding domains to that of LexA. The predictions of LexA regulons in the 26 cyanobacterial genomes that harbor a *lexA *gene are summarized in Table 2.

**Table 2:** Summary of genome-wide LexA-binding site predictions in the 26 cyanobacterial genomes

Genome	Number of TUs	Number of genes	Score at p < 0.05	LOR at p < 0.05	No. of sites at p < 0.05	Score at p < 0.01	LOR at p < 0.01	No. of sites at p < 0.01
Acaryochloris_marina_MBIC11017	4507	6254	6.52	-0.143	213	7.02	0.007	48
Anabaena_variabilis_ATCC_29413	3967	5043	6.44	0.549	403	6.96	0.911	107
Cyanothece_PCC_8801	2989	4260	6.24	0.01185	169	6.73	0.22	38
Microcystis_aeruginosa_NIES_843	4736	6312	6.18	0.4941	256	6.70	0.326	73
Nostoc_punctiforme_PCC_73102	4798	6087	6.40	0.323	356	6.88	0.647	89
Nostoc_sp_PCC7120	4136	5366	6.44	0.534	389	6.88	0.995	122
Prochlorococcus_marinus_AS9601	1078	1921	6.34	0.671	107	6.74	1.330	50
Prochlorococcus_marinus_CCMP1375	1110	1883	6.37	0.070	53	6.87	-0.098	9
Prochlorococcus_marinus_MED4	961	1717	6.36	0.454	79	6.79	0.847	29
Prochlorococcus_marinus_MIT9313	1406	2269	6.63	-0.149	61	7.08	0.536	24
Prochlorococcus_marinus_MIT_9211	1081	1855	6.28	0.385	75	6.77	1.036	26
Prochlorococcus_marinus_MIT_9215	1135	1983	6.30	0.668	109	6.79	1.407	42
Prochlorococcus_marinus_MIT_9301	1070	1907	6.30	0.768	117	6.74	1.345	54
Prochlorococcus_marinus_MIT_9303	1881	2997	6.52	-0.0958	83	7.01	0.527	36
Prochlorococcus_marinus_MIT_9312	1013	1810	6.33	0.567	93	6.79	1.339	40
Prochlorococcus_marinus_MIT_9515	1088	1906	6.34	0.564	99	6.78	1.247	43
Prochlorococcus_marinus_NATL1A	1393	2193	6.30	0.495	131	6.81	1.138	43
Prochlorococcus_marinus_NATL2A	1175	1892	6.33	0.678	117	6.89	1.107	36
Synechococcus_CC9311	1700	2892	6.50	-0.153	69	7.14	0.218	19
Synechococcus_CC9605	1466	2645	6.54	-0.135	64	7.12	0.305	18
Synechococcus_CC9902	1288	2307	6.52	-0.103	63	7.00	0.786	22
Synechococcus_PCC_7002	2003	2823	6.31	-0.196	91	6.79	-0.156	20
Synechococcus_RCC307	1303	2535	6.74	-0.0904	35	7.33	0.085	8
Synechococcus_sp_WH8102	1296	2519	6.62	-0.254	59	7.18	0.142	22
Synechococcus_WH_7803	1303	2535	6.56	-0.278	56	7.36	0.623	16
Synechocystis_PCC6803	1312	2533	6.52	-0.388	79	7.00	-0.256	19

### Conservation and diversity of the putative LexA regulons in cyanobacteria

**Figure 3:**
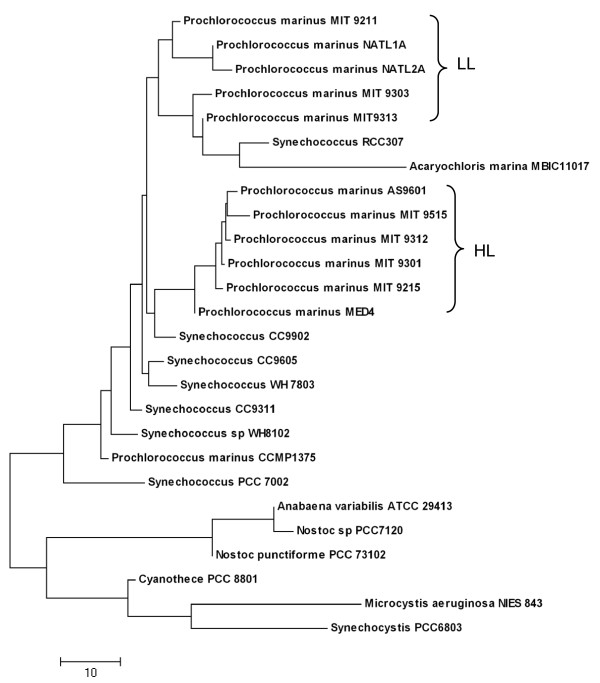
**Conservation relationships among the predicted LexA regulons in the 26 cyanobacterial genomes**. The tree is based on the pairwise conservation of the predicted LexA regulons in the 26 cyanobacterial genomes (see Methods).

**Figure 4:**
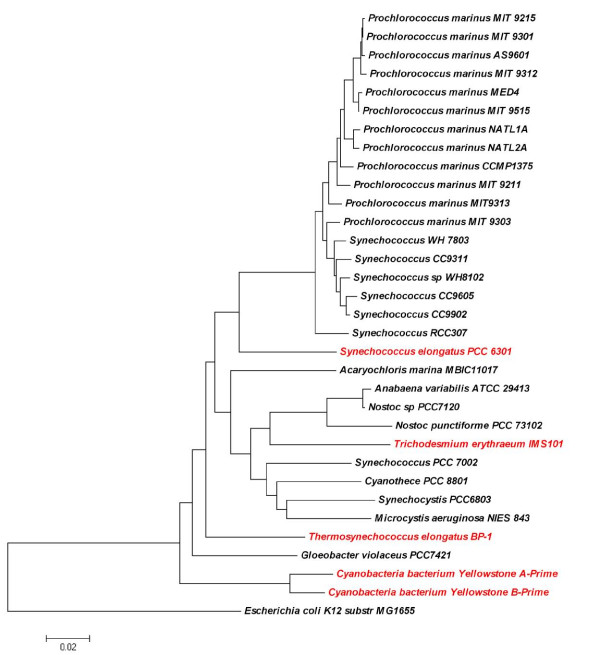
**Phylogenetic relationships of 32 cyanobacterial genomes based on the 16S rRNA genes**. The tree is rooted with the 16S rRNA gene of *E. coli *K12. Bootstrap values are shown on the nodes. Cyanobacterial genomes that do not encode a *lexA *gene are shown in red.

To investigate how well the predicted LexA regulons in the 26 cyanobacterial genomes are conserved, we constructed a LexA regulon conservation tree based on the pairwise comparison of the predicted LexA regulons in these genomes (see Methods). As shown in Figure 3, these genomes are divided into two groups. Interestingly, one group is exclusively comprised of marine strains, and the other group contains the remaining genomes isolated from different non-marine habitats. In the former group, high light (HL) adapted and low light (LL) adapted ecotypes are largely grouped into two sub-groups. The results suggest that the composition of LexA regulons is dependent on the habitat of the organisms to a large extent. The general topology of the tree (Figure 3) is basically consistent with both the 16S rRNA gene tree (Figure 4) and the LexA protein tree of these genomes (Figure 1A). Furthermore, both the HL and LL adapted marine sub-groups are very compact, indicating that the predicted LexA regulons in both sub-groups are relatively conserved. In contrast, the species in the non-marine habitats are not so close to one another (Figure 3), suggesting that the putative LexA regulons in these genomes share few genes with one another except for the closely related *Anabaena variabilis *ATCC 29413 and *Nostoc sp. *PCC7120. The tree also indicates that *Microcystis aeruginosa *NIES 843 and *Synechocystis sp. *PCC6803 have the most distinct LexA regulons from other cyanobacterial genomes (Figure 3).

### Functional classification of putative LexA regulons in cyanobacteria

Predicted members of LexA regulons in the 26 cyanobacteria that harbor a *lexA *gene are listed in Tables S1-S26 in Additional file 4, their functions can be summarized as follows.

#### 1. SOS response system

As shown in Table S63 in Additional file 6, all the 33 cyanobacterial genomes included in this study encode a few SOS response genes found in *E. coli*. Several of the SOS genes in some of the 26 genomes that harbor a *lexA *gene bear a high-scoring putative LexA-binding site in their regulatory regions (Table S1-26 in Additional file 4). In particular, two of the core SOS response genes [5,6], namely, *recA *and *lexA*, are among the most conserved putative LexA targets in the 26 cyanobacterial species/strains (Table S64 in Additional file 6). In addition, the *umuC *and *umuD *genes encoded in 13 genomes are also predicted to bear a putative LexA-binding site in their promoter regions (Table S64 in Additional file 6, Table 3 and Additional file 4). These results suggest that as in *E. coli*, the SOS response in most cyanobacteria might still be regulated by LexA. However, the other SOS response genes were found to bear a putative LexA-binding site only in a few genomes (Table 3). For instance, a high-scoring LexA-binding site for the *ruvB *gene encoding Holliday junction DNA helicase B was found only in HL adapted *Prochlorococcus *ecotypes MIT9312, MIT9515, MIT9215, MED4 and AS9601. Moreover, in the case of the nucleotide excision repair (NER) genes *uvrA, B, C and D*, which are under the regulation of LexA in *E. coli *[10], we were able to identify putative LexA-binding sites only in the promoter regions of the *uvrA *and *uvrB *in *Nostoc sp*. PCC7120 and the promoter region of uvrD in *Prochlorococcus marinus *MIT 9312 (Tables 3). Thus, it is likely that the NER process in the remaining genomes is regulated by some transcription factor other than LexA, given that *uvr *genes are present in all the 32 cyanobacterial genomes analyzed in this study, including those that do not encode a *lexA *gene (Table S63 in Additional file 6). These results are consistent with the earlier observation that LexA target genes in bacteria are highly diversified in order for them to adapt to different ecological niches [5,6].

**Table 3:** Putative LexA regulon members involved in various biological processes

Genomes	SOS	Photo-synthesis	Transporters
Acaryochloris marina_MBIC11017	lexA recA dnaK groEL umuCD		4624
Anabaena_variabilis_ATCC_29413	lexA recA dnaJ sbcC	psbA	4997 4148 4995
Cyanothece_PCC_8801	lexA recA		
Microcystis_aeruginosa_NIES_843	recA ssb	ndhH ycf4	pstB2
Nostoc_punctiforme_PCC_73102	lexA recA sbcC F4123		F3763
Nostoc_sp PCC7120	lexA recA uvrA uvrB dnaKJ		alr5147
Prochlorococcus_marinus_AS9601	recA ruvB umuCD	psbY	11511
Prochlorococcus_marinus_CCMP1375	recA sbcD groES groEL		
Prochlorococcus_marinus_MED4	recA umuCD ruvB	psbY	
Prochlorococcus_marinus_MIT9313	lexA umuCD		
Prochlorococcus_marinus_MIT_9211	recA umuCD		
Prochlorococcus_marinus_MIT_9215	recA umuCD ruvB	psbY	08441
Prochlorococcus_marinus_MIT_9301	recA umuCD	psbY	11521 02331
Prochlorococcus_marinus_MIT_9303	lexA umuCD		21241 15661
Prochlorococcus_marinus_MIT_9312	recA ruvB umuCD uvrD	psaA psbY	0561
Prochlorococcus_marinus_MIT_9515	recA ruvB dnaK	psbY	06251
Prochlorococcus_marinus_NATL1A	lexA recA		
Prochlorococcus_marinus_NATL2A	lexA recA	psaM	
Synechococcus_CC9311	recA umuCD		2443
Synechococcus_CC9605	recA umuCD		2635
Synechococcus_CC9902	recA umuCD		0850
Synechococcus_PCC7002	recA	psaF	
Synechococcus_RCC307	lexA		
Synechococcus_sp_WH8102	recA umuCD ruvC		2111 0959
Synechococcus_WH7803	recA	ndhH	
Synechocystis_PCC6803		psbB	0467

On the other hand, in *Synechococcus *PCC7002, *Synechococcus *RCC307 and *Synechococcus *WH7803, LexA boxes were only detected for one of the core SOS response genes, i.e., SYNPCC7002_A0426 (*recA*) in *Synechococcus *PCC7002, SynRCC307_1756 (*lexA*) in *Synechococcus *RCC307 and SynWH7803_0439 (*recA*) in *Synechococcus *WH7803, although these genomes all encode a *lexA *gene and other core SOS response genes, such as *recA *(SYNPCC7002_A0426, SynRCC307_2111 and SynWH7803_0439,) and *ruvB *(SYNPCC7002_A1390, SynRCC307_1756 and SynWH7803_0185), *umuC *(SynRCC307_0043 and SynWH7803_1080,) and *umuD *(SynRCC307_0042 and SynWH7803_1081). Since only one single SOS response gene bears a putative LexA box in these genomes, it is likely that the role of LexA in the regulation of the SOS response in these genomes might have been attenuated. The case of *Synechocystis sp. *PCC6803 seems to go even further in this direction as detailed below.

As indicated previously [16,18], the LexA protein of *Synechocystis sp*. PCC6803 is unusual in two aspects compared to those in the other genomes analyzed in this study. First, as shown in Figure S4 in Additional file 2, the Ala-Gly dyad in the N-terminus of LexA responsible for auto-cleavage of the protein in all other cyanobacteria as well as in *E. coli *and *B. subtilis *[16] are replaced by Gly-Gly in *Synechocystis sp*. PCC6803. Second, the reactive residue Ser (Ser^119 ^of LexA in *E. coli*) that attacks the Ala-Gly peptide bond is replaced by Asp of LexA in *Synechocystis sp*. PCC6803 [16,18]. It has been shown that SOS induction cannot be initiated by a non-cleavable LexA repressor [10,16]. Therefore, it is highly likely that LexA in *Synechocystis sp*. PCC6803 cannot undergo the auto-cleavage reaction in response to DNA damage, and it might have adopted a different function other than the canonical SOS response regulator seen in *E. coli *and *B. subtilis *[32]. This argument is consistent with the observation that *Synechocystis sp*. PCC6803 has a notably larger branch length in the 27 LexA protein tree (Figure 1A), but this is not seen in the 16S rRNA gene tree (Figure 4).

Although the *Synechocystis sp*. PCC6803 genome harbors some core SOS response genes including *lexA *and *recA *(Table S63 in Additional file 6), none of them belongs to our predicted LexA regulon at a *p*-value < 0.01 (Table S26 in Additional file 4 and Table 3). The *mutS *(sll1772) gene is the only gene that is likely to be in involved in DNA mismatch repair, while bearing a putative LexA binding site in the genome. However, the orthologs of *mutS *is not under the regulation of LexA in *E. coli *[2,33] or within the putative LexA regulon of any other cyanobacteria (Table S1-26 in Additional file 4). These results suggest that at least most of SOS response genes are not under the regulation of LexA in *Synechocystis sp*. PCC6803. Indeed, using microarray gene expression profiling in response to *lexA *depletion, Domain *et al. *[21] concluded that LexA in *Synechocystis sp*. PCC6803 might be involved in carbon metabolism or controlled by carbon availability rather than the regulation of SOS response. However, our predicted LexA regulon in *Synechocystis sp*. PCC6803 (Table S26 in Additional file 4) has no intersection with the LexA-responsive genes identified by Domain *et al. *[21]. Since the LexA-binding sites that were experimentally characterized [17,20] in *Synechocystis sp*. PCC6803 are different from the sequences in our scanning profile, and considering the distinct nature of the LexA protein in *Synechocystis sp*. PCC6803 indicated above, it would be particular interesting to determine by experiment the function of the predicted sites in this genome.

Thus, although *Synechocystis sp*. PCC6803 clearly harbors the components of a basic SOS response system (Table S63 in Additional file 6), it is probably no longer under the regulation of LexA. Accordingly, LexA in this genome might have adopted a different function. Thus, the loss of the original function of LexA in *Synechocystis sp. *PCC6803 is coupled with the loss of the sequence constraint, thereby accelerating its divergence from other cyanobacterial LexA proteins, at both the sequence and functional levels. On the other hand, given the importance of the SOS response in cell survival, it is highly likely that the transcriptional regulator of the SOS response system in *Synechocystissp*. PCC6803 has been replaced by another protein.

#### 2. Other cellular processes

Interestingly, we also found putative LexA-binding sites in the regulatory regions of genes that participate in various cellular processes in these 26 cyanobacterial genomes (Table 3). The major cellular processes that are likely under the regulation of LexA are summarized below.

##### 2.1 Photosynthesis

Putative LexA-binding sites were predicted for the following photosynthetic genes in the 26 cyanobacteria that harbor a *lexA *gene with p < 0.01(Table 3, Table S1-26 in Additional file 4): Ava_3553, A9601_12231, PMM1117, P9215_12531, P9301_12241, PMT9312_1128, and P9515_12081, coding for a photosystem II reaction center protein PsbY; slr0906, coding for the photosystem II CP47 protein; and MAE_44810, PMT9312_1615, PMN2A_1682a and SYNPCC7002_A1008, coding for a protein involved in photosystem I. These results suggest that the SOS response system might have cross-talk with photosynthesis in those genomes.

##### 2.2. Transporters

Around 20 genes encoding transporters were predicted to bear a putative LexA box (Table 3). Most of them belong to the ABC transporter proteins, including AM1_4624 in *Acaryochloris marina *MBIC11017, Ava_4995 in *Anabaena variabilis *ATCC29413, MAE_18340 in *Microcystis aeruginosa *NIES843, Npun_F3763 in *Nostoc punctiforme *PCC73102, alr5147 in *Nostoc sp *PCC7120, P9215_08441 in *Prochlorococcus marinus *MIT9215, P9303_15661 in *Prochlorococcus marinus *MIT9303, P9515_06251 in *Prochlorococcus marinus *MIT9515, SYNW2111 in *Synechococcus sp *WH8102 and slr0467 in *Synechocystis sp. *PCC6803. In addition, several toxin and antibiotics exporters were identified to have a putative LexA-binding site in their regulatory regions, including cadmium resistance transporter Ava_4997 in *Anabaena variabilis *ATCC29413; MFS (major facilitator superfamily) multidrug efflux transporter P9301_11521 in *Prochlorococcus marinus *MIT9301 and A9601_11511 in *Prochlorococcus marinus *AS9601; multidrug efflux ABC transporter P9515_06251 in *Prochlorococcus marinus *MIT9515 and SYNW0959 in *Synechococcus sp *WH8102; putative ABC transporter/multidrug efflux family protein SYNW2111 in *Synechococcus sp *WH8102; drug exporter-1 ABC transporter ATPase subunit AM1_4624 in *Acaryochloris marina *MBIC11017. These findings are interesting since it has been shown that the SOS response system is related to drug resistance in *E. coli *[34,35] and *Staphylococcus aureus *[35-37] by mechanisms that are not fully understood. It was reported that the *vP2449 *gene encoding a toxin exporter responsible for xenobiotic resistance in *Vibrionales parahaemolyticus *was under the direct control of LexA [38]. Therefore, it is likely that these drug resistance genes are regulated by LexA, thereby coupling the SOS response to drug resistance in these cyanobacteria.

### The origin of the *lexA *gene in cyanobacteria

As indicated earlier, 27 of the 32 cyanobacterial genomes analyzed evidently harbor a *lexA *ortholog, while the remaining five genomes do not, even when being scrutinized by more sensitive sequence search methods such as PSI-BLAST (data not shown). The five cyanobacteria lacking a LexA are *Synechococcus sp. *JA-3-3Ab A-Prime, *Synechococcus sp. *JA-2-3B'a(2-13) B-Prime, *Synechococcus elongatus *PCC6301, *Trichodesmium erythraeum *IMS101 and *Thermosynechococcus elongatus *BP-1. However, the core SOS response genes remain in these five genomes (Table S63 in Additional file 6). In the tree of 183 detected LexA proteins in 598 sequenced genomes (Figure S2 in Additional file 3), the 26 cyanobacterial LexA proteins that are detected by BDBH (see Methods) form a monophyletic group while LexA in *Gloeobacter violaceus *PCC7421 is clustered with the group of α-proteobacteria. Furthermore, the topology of the 16S rRNA gene tree (Figure 4) and the LexA tree/subtree of 27 cyanobacterial genomes (Figure 1A and Figure S2 in Additional file 3) are quite similar. This result suggests that *lexA *in the 26 cyanobacterial genomes (excluding *Gloeobacter violaceus *PCC7421) is likely to be vertically inherited from the last common ancestor of cyanobacteria. However, *Gloeobacter violaceus *PCC7421 might have lost its LexA protein during evolution and obtained an ortholog later through horizontal transfer from an α-proteobacterium. The five genomes that lack a *lexA *gene do not form a monophyletic group in the 16S rRNA gene-based phylogenetic tree of these 32 cyanobacteria (Figure 4). In particular, *Synechococcus elongatus *PCC6301, and *Trichodesmium erythraeum *IMS101 are spread in a clade whose members except these two genomes harbor a *lexA *gene. The most parsimonious explanation of this distribution would be that these two genomes *Synechococcus elongatus *PCC6301 and *Trichodesmium erythraeum *IMS101 lost their *lexA *genes through two independent events (one for each genome) to adapt to their corresponding environments during the course of evolution. Furthermore, the remaining three genomes, *Thermosynechococcus elongatus *BP-1, *Synechococcus sp. JA-3-3Ab *A-prime and *Synechococcus sp. JA-2-3B'a (2-13) *B-prime, which do not possess a *lexA *gene, branch earlier from the others (Figure 4). A plausible explanation of this distribution would be that these genomes lost their *lexA *genes inherited from the last common ancestor of cyanobacteria during the course of evolution. Interestingly, all these three genomes are thermophilic, their extreme ecological niches might facilitate the loss of the *lexA *gene. Since the core SOS response genes remain in these five genomes (Table S63 in Additional file 6) after *lexA *was lost, they might have been hijacked by another transcription factor given the importance of the regulation of the SOS response genes for cell survival. The genomes that lost their *lexA *gene appear to have lost LexA-binding sites (Figure S3 in Additional file 2). Alternatively, these five cyanobacteria might still harbor a *lexA *gene that has largely diverged from the others' during evolution to such a level that our method could not detect them.

In addition, it has been suggested that the *lexA *gene was derived from gram-positive bacteria, which then spread into cyanobacteria and fibrobacteres. Then α-proteobacteria acquired *lexA *from cyanobacteria [5,6,16]. Our phylogenetic analysis of the LexA proteins and their binding sites supports such an argument. As mentioned before, cyanobacterial LexA proteins are more closely-related to those in gram-positive bacteria and α-proteobacteria than those in the other groups (Figure S2 in Additional file 3), and the predicted LexA-binding sites in cyanobacteria are clustered together with those in the gram-positive bacterium *B. subtilis *and in α-proteobacteria, but are far away from those in *E. coli *(Figure S1 in Additional file 2).

Moreover, Erill *et al*. [27] have suggested that there is a common set of genes in the LexA regulon of proteobacteria and gram-positive bacteria: recA, uvrA, ssb, and ruvC. However, our predicted LexA regulons in cyanobacteria do not always include this set of genes. Thus, the concept of a common set of SOS response gene in its more general form warrants further scrutinization.

## Conclusions

In this study we have predicted LexA-binding sites and analyzed the putative LexA regulons in 26 cyanobacterial genomes that harbor a *lexA *gene using a highly efficient motif scanning and regulon prediction algorithm. In most *lexA*-containing cyanobacterial genomes, some SOS response genes bear a putative LexA box. Some genes involved in various cellular processes such as photosynthesis, drug resistance, etc. are also predicted to bear a putative LexA box in their promoter regions. However, in *Synechocystis sp. *PCC6803, LexA might have adopted a new function and no longer be in charge of the SOS response genes. In some genomes, *lexA *was likely lost during the course of evolution accompanied by the loss of its binding sites. The SOS response genes in these genomes that appear to lack a *lexA *gene might be regulated by another or multiple transcription factors. Moreover, we conclude that cyanobacteria inherited the *lexA *gene from their last common ancestor; however, substantial genome-wide turnover seems to have led to the high degree of variation of the LexA regulons in some species during evolution.

## Methods

### Materials

The sequences and annotation files of 33 sequenced cyanobacterial and the other genomes were downloaded from NCBI at ftp://ftp.ncbi.nih.gov/genomes/Bacteria/. The cyanbacterial genomes used in this study include: *Acaryochloris marina *MBIC11017 (MBIC11017), *Anabaena variabilis *ATCC 29413 (ATCC29413), *Synechococcus sp*. JA-3-3Ab (A-Prime), *Synechococcus sp*. JA-2-3B'a (2-13) (B-Prime), *Cyanothece sp*. PCC 8801(PCC8801), *Gloeobacter violaceus *PCC7421 (PCC7421), *Microcystis aeruginosa *NIES 843 (NIES843), *Nostoc punctiforme *PCC 73102 (PCC73102), *Nostoc sp*. (PCC7120), *Prochlorococcus marinus *AS9601 (AS9601), *Prochlorococcus marinus *CCMP1375 (CCMP1375), *Prochlorococcus marinus *MED4 (MED4), *Prochlorococcus marinus *MIT9313 (MIT9313), *Prochlorococcus marinus *MIT 9211 (MIT9211), *Prochlorococcus marinus *MIT 9215 (MIT9215), *Prochlorococcus marinus *MIT 9301 (MIT9301), *Prochlorococcus marinus *MIT 9303 (MIT9303), *Prochlorococcus marinus *MIT 9312 (MIT9312), *Prochlorococcus marinus *MIT 9515 (MIT9515), *Prochlorococcus marinus *NATL1A (NATL1A), *Prochlorococcus marinus *NATL2A (NATL2A), *Synechococcus sp*. CC9311 (CC9311), *Synechococcus sp*. CC9605 (CC9605), *Synechococcus sp. *CC9902 (CC9902), *Synechococcus sp. *PCC 7002 (PCC7002), *Synechococcus sp*. RCC307 (RCC307), *Synechococcus *WH 7803 (WH7803), *Synechococcus elongatus *PCC 6301 (PCC6301), *Synechococcus sp*. WH8102 (WH8102), *Synechocystis sp*. PCC6803 (PCC6803), *Synechocystis sp*. PCC7942 (PCC7942), *Thermosynechococcus elongates *BP-1 (BP-1) and *Trichodesmium erythraeum *IMS101 (IMS101).

### Prediction of transcription units

We predicted the operon structures in cyanobacterial genomes using the operon prediction algorithm developed by Dam et al. [39]. The algorithm is based on the integration of both genome-specific and comparative genomic information. In this work, both the multi-gene operon and singleton operon (containing one gene) are considered as a transcription unit (TU), and the upstream intergenic sequence of the first open reading frame is not considered as a part of the operon.

### Prediction of orthologs

We used the bi-directional best hit (BDBH) method based on BLASTP searches with an *E*-value cut-off of 10^-10 ^for both directions to predict orthologous protein pairs between any two proteomes. The BDBH method assumes that a cross-species protein pair are orthologous if each protein returns the other as the best hit in the whole proteome comparison [40].

### Phylogenetic analysis

To construct the phylogenetic tree of LexA in cyanobacteria, multiple sequence alignment of the LexA amino acid sequences from 27 cyanobacterial genomes and the *E. coli *K12 genome were made using ClustalW implemented in MEGA [41] with default settings. The phylogenetic tree was then constructed using the neighbor-joining method with Poisson correction model in MEGA. *E. coli *LexA was placed as the outgroup of the tree. To construct the species tree, the DNA sequences of 16S ribosomal RNA genes from the 32 cyanobacteria and *E. coli *were aligned using ClustalW with manual adjustment by removing the unalignable regions. A neighbor-joining tree was then constructed with *E. coli *K12 being the outgroup using the Kimura 2-parameter model. Statistical significance at each node in the trees was evaluated using 500 bootstrap resamplings.

To construct the LexA protein tree across cyanobacteria, gram-positive bacteria, α-proteobacteria, δ-proteobacteria and γ-proteobacteria and some other bacteria strains/species (Figure S2 in Additional file 3), we first downloaded 598 sequenced microbial genome sequences from NCBI, and then identified LexA orthologs in them by the BDBH method described above. Multiple sequence alignments of these LexA sequences were made using ClustalW implemented in MEGA[41] with default settings. The phylogenetic tree was then constructed in the same way as the 27 LexA protein tree.

The phylogenetic tree (Figure S1 in Additional file 2) of LexA-binding sites in cyanobacteria, *B. subtilis*, α-proteobacteria and *E. coli *K12 was generated by the STAMP web tool [42] with the default alignment parameters: Pearson correlation coefficient for column comparison metric; ungapped Smith-Waterman for pair-wise alignment. The phylogenetic tree was constructed using the UPGMA method implemented in STAMP [42].

### Phylogenetic footprinting and construction of LexA-binding sites in cyanobacteria

The previous study by Mazon *et al. *[16] characterized the LexA boxes associated with two genes: alr4908 (*lexA*) and all3272 (*recA*). Four putative LexA boxes were also identified in the promoter regions of alr3716 (*uvrA*), alr0088 (*ssb*), alr4905, and all4790 in *Nostoc sp *PCC7120 in that study. The orthologs (if they exist) of these six genes in PCC7120 were identified in the other 25 cyanobacterial genomes which harbor a *lexA *gene. We pooled the entire upstream inter-TU regions of these six genes in the target genome *Nostoc sp*. PCC7120 as well as those of the TUs containing at least one of the orthologs of these six genes in other cyanobacteria. If the length of the inter-TU region is longer than 800 bases, then only the immediate upstream 800 bases were extracted. Two motif finding programs, MEME [25,43] and BioProspector [26], were then applied to these pooled inter-TU regions to identify palindromic 14-mers as putative LexA-binding sites in these sequences according to previous studies [16]. MEME applies an expectation maximization method to fit a two-component finite mixture model and returns the identified motifs with an E-value[43], while BioProspector employs a Gibbs sampling strategy and estimates the significance of the identified motif by a Monte Carlo method [44]. These two programs were selected as they are widely used and often have complementary predictions [45,46]. MEME identified 45 putative LexA-binding sites with an overall E-value of 1.4e-026 for its most significant predicted motif, while BioProspector detected 39 putative LexA boxes in its most significant predicted motif (see Additional file 1 for details). High-scoring putative LexA-binding sites from either program were selected to build the LexA-binding sites profile (Table 1) in cyanobacteria. Sequence logos of binding sites were created using the Weblogo server [47].

### Genome wide prediction of LexA-binding sites

We used the profile constructed above to scan the inter-TU regions of the genomes to predict all putative LexA-binding sites using a scanning algorithm that we previously developed [29-31]. This algorithm is briefly described as follows.

For each predicted TU *U*(*g_1_,g_2_,...,g_n_*) composed of genes *g_1_,g_2_,...,g_n _*in genome G, we extracted its upstream inter-TU regions and the first 40 bases of coding region(if its length is longer than 800 bases, then only the immediate upstream 800 bases were extracted), denoted as I_*U*(*g1,g2,...,gn*)_. The set of all the I_*U*(*g1,g2,...,gn*) _in this genome is denoted as I*_U_*. To find the best matching substring in a sequence *t *in I_U _(*t *∈ *I_U_*) when scanned by profile M, we use the following scoring function:

(1) $$s_{M}(t)=\underset{h\subset t}{\max}\sum_{i=1}^{l}I_{i}\ln\frac{p(i,h(i))}{q(h(i))},$$

(2) $$I_{i}=\left(\sum_{b\in \{A,C,G,T\}}p(i,b)\ln\frac{p(i,b)}{q(b)}\right)/a,$$

(3) $$\begin{array}{c} a=\frac{n+1}{n+4}\ln(n+1)-\ln(n+4)\\ -\frac{1}{n+4}\sum_{b\in \{A,C,G,T\}}\ln q(b)-\frac{n}{n+4}\ln\underset{b\in \{A,C,G,T\}}{\min}q(b), \end{array}$$

where *l *is the length of the binding sites of profile M, *h *any substring of sequence t with length *l *(i.e. each *l*-mer of the sequence *t*), *h(i) *the base at the *i*-th position of *h*, *p(i,b) *the frequency of base *b *at position *i *in *M*, *q(b) *is the frequency of base *b *in the aggregated inter-TU regions for the organism, *I_i _*is basically the information content or the relative entropy of the column [28,48] divided by a normalization factor *a*, *a *is the upper limit of the information content *I_i _*for this column to keep *I_i _*∈ [0, 1], *n *the number of binding sites for constructing the profile M. To avoid zero value of the numerator *p(i,b)*, a pseudo count 1 is added to the counts of the each base {A, C, G, T} in column *i*.

To show the derivation of the normalization factor *a*, we considered the extreme case: for a column *i *of profile M containing *n *binding sites, the more conserved the column is, the higher its information content *I_i _*will be, and the maximum information content for column *i *occurs when this column is completely homogeneous. That is, all sequences have the same nucleotide, say, A at that position, and this nucleotide has the smallest background frequency, *q(A)*, noted as *q_0_*. Thus, after adding one pseudocount to the counts of each of the four nucleotides to column *i*, the frequency of base A of column *i *in the profile will therefore be (n+1)/(n+4), and 1/(n+4) for the other three nucleotides. Then the upper limit *a *of the prenormalized *I_i _*as shown by formula (3) can be derived as follows.

(4) $$\begin{array}{c} I_{i}=\sum_{b\in \{A,C,G,T\}}p(i,b)\ln\frac{p(i,b)}{q(b)}\\ \leq \frac{n+1}{n+4}\ln\frac{n+1}{(n+4)q_{0}}\\ +\sum_{b\in \{A,C,G,T\}}\frac{1}{n+4}\ln\frac{1}{\left(n+4)\cdot q(b\right)}\\ -\frac{1}{n+4}\ln\frac{1}{(n+4)q_{0}}\\ =\frac{n+1}{n+4}\ln(n+1)-\frac{n+1}{n+4}\ln(n+4)-\frac{n+1}{n+4}\ln q_{0}\\ +\frac{1}{n+4}(-4\ln(n+4)-\sum_{b=A}^{T}\ln q(b))\\ +\frac{\ln(n+4)}{n+4}+\frac{\ln q_{0}}{n+4}\\ =-\frac{4}{n+4}\ln(n+4)-\frac{1}{n+4}\sum_{b=A}^{T}\ln q(b)\\ \begin{array}{c} -\frac{n}{n+4}\ln(n+4)-\frac{n}{n+4}\ln q_{0}+\frac{n+1}{n+4}\ln(n+1) \end{array}\\ =-\ln(n+4)-\frac{1}{n+4}\sum_{b=A}^{T}\ln q(b)\\ -\frac{n}{n+4}\ln q_{0}+\frac{n+1}{n+4}\ln(n+1)\\ =a \end{array}$$

where $q_{0}=q(A)=\underset{b\in \{A,C,G,T\}}{\min}q(b)$.

Intuitively, we slide a window of length *l *across sequence *t *with the profile *M*, and return the substring *h *with the highest score defined by (1).

Since true regulatory binding sites are likely to be more conserved than other inter-TU sequences and thus tend to be shared by closely related orthologous genes. For each genome (considered as a target genome), we reward its putative binding sites appeared to be conserved in regions upstream from orthologous genes in other genomes. To do this, we assume a transcription unit *U*(*g_1_,g_2_,...,g_n_*) in the target genome *G *is composed of n genes. Gene *g_i _*(*i *= 1...*n*) has orthologs in *m_i _*genomes *G_1_*, *G_2_*, ..., $G_{m_{i}}$, and *o_k _*(*g_i_*) is the upstream inter-TU sequence associated with the orthologous gene *g_i _*in genome *G_k _*(for a graphic explanation, see Figure S5 in Additional file 2). Then the *s_M_(t) *score for the inter-TU sequence *t *upstream from *U*(*g_1_,g_2_,...,g_n_*) in genome G can be increased by a term *A_max_(g*_i_):

(5) $$s(t)=s_{M}(t)+A_{\max}(g_{i})$$

where *A_max_(g_i_) *is the value calculated for gene *g_i _*whose orthologs across other genomes have the maximum average of the product of two terms:

(6) $$\begin{array}{l} A_{\max}(g_{i})\\ =\underset{1\leq i\leq n}{\max}\left\{\begin{array}{l} \text{average[(similarity between the two sites)}\\ \text{*(score of this orthologous site)] } \end{array}\right\}\\ =\underset{1\leq i\leq n}{\max}\left\{average[\frac{l-d_{i,k}}{l}s_{M}(o_{k}(g_{i}))]\right\} \end{array}$$

where *d_i,k _*is the Hamming distance between the sequence *h *detected by the profile *M *in sequence *t *and the corresponding sequence in, *o_k _*(*g_i_*) and *l *is the length of the binding sites in profile *M*.

Since the orthologs of genes of a transcription unit in one organism may not comprise a single transcription unit in another. For *U*(*g_1_,g_2_,...,g_n_*) in target genome G, orthologs of *g_1_,g_2_,...,g_n _*may be separated into different TUs in other genomes, therefore we evaluated the orthologous inter-TU sequences for each gene in (*g_1_,g_2_,...,g_n_*), and chose the gene *g_i _*whose orthologs across other genomes have the maximum average of the product of two terms indicated above. Then by combining formula (5) and (6), the refined score of the best putative binding site in sequence *t *can be defined as:

(7) $$s(t)=s_{M}(t)+\underset{1\leq i\leq n}{\max}\frac{1}{m_{i}}\sum_{k=1}^{m_{i}}\frac{l-d_{i,k}}{l}s_{M}(o_{k}(g_{i}))$$

### Statistical significance of predicted binding sites

To evaluate the extent to which a putative binding site with a score *s *or higher can be found purely by chance, we randomly extracted coding sequence with the same length as I_*U*(*g1,g2,...,gn*)_, denoted as C_*U*(*g1,g2,...,gn*)_. All the C_*U*(*g1,g2,...,gn*) _extracted in genome G form the set C*_U_*. The score of an extracted sequence t (t ∈ *C_U_*) scanned by a profile M is also defined by formula (1). Note that each randomly chosen C_*U*(*g1,g2,...,gn*) _has nothing to do with *U*(*g_1_,g_2_,...,g_n_*). Therefore, when incorporating the additional score from reference genomes (formula (7)), the coding sequence *o_k _*(*g_i_*) is unlikely the coding sequence associated with the orthologous genes of *g_1_,g_2_,...,g_n _*in a reference genome *G_k _*as it is a randomly chosen one. To avoid possible biased sampling of coding sequences for each I_*U*(*g1,g2,...,gn*) _in *I_U_*, we randomly extracted 300 coding sequences C_*U*(*g1,g2,...,gn*) _sharing the same length as I_*U*(*g1,g2,...,gn*)_. These randomly chosen coding regions for all the *U*(*g_1_,g_2_,...,g_n_*) in genome G form a sequence set *C_U_*, then each sequence in the set *C_U _*was scanned using formula (7). Let *S*(*I_U_*) and *S*(*C_U_*) be the set of scores of binding sites found in *I_U _*and *C_U_*, respectively, and *P*(*S*(*t*) >*s*) be the cumulative probability of finding a binding site in a sequence *t *(*t *∈ *I_U _*(or) *t *∈ *C_U_*) with a score *S*(*t*) >*s *as defined by equation (7). Next, the false positive rate, $p(S_{C_{U}}>s)$ can be used to evaluate the statistical significance of the motif score *s *of a inter-TU sequence. $p(S_{C_{U}}>s)$ is actually the fraction of coding sequences bearing a putative binding site with a score higher than *s *in the coding sequences set *C_U _*in genome G. In other words, it describes the extent to which one can find a motif with a score higher than *s *by chance. Thus, it can be considered as an empirical p-value for a binding site score *s*. A cut-off score *s *corresponding to a *p-*value *<*0.01 is used for the LexA-binding site and regulon prediction in each genome in this study.

To evaluate the confidence of our overall predictions in inter-TU regions in one genome, we used a log odds ratio (*LOR*) to compare the probability of finding a putative binding site in an inter-TU region with the probability of finding a putative binding site in a randomly extracted coding region by considering all the extracted *I_U_s *and *C_U_s *in a genome. We estimated the statistical significance of the predictions using the *LOR *function defined as

(8) $$LOR(s)=\ln\frac{p(S_{I_{U}}>s)}{p(S_{C_{U}}>s)}.$$

The *LOR *function for a genome is the log-odds ratio of the fraction of the inter-TU sequences containing a binding site with a score higher than s to the fraction of the randomly selected coding sequences containing a binding site with a score higher than the same *s *in the genome. Accordingly, a monotonic increase in positive *LOR *with the increase in the motif score in a genome would suggest that this genome is likely to contain some high-scoring LexA-binding sites.

### Analysis of the conservation of LexA regulons in cyanobacteria

We defined the conservation (*c_ij_*) between two regulons *R_i _*and *R_j _*from genome *i *and *j*, respectively, as,

(9) $$c_{ij}=\frac{\left|R_{i}\cap R_{j}\right|}{\left|R_{i}\cup R_{j}\right|}=\frac{\left|R_{i}\cap R_{j}\right|}{\left|R_{i}|+|R_{j}|-|R_{i}\cap R_{j}\right|}$$

Where |*R*_*i *_∩ *R*_*j*_| is the number of orthologous genes shared by both regulons *R_i _*and *R_j_*. We took the reciprocal of $\frac{1}{c_{ij}}$ as the distance *d_ij _*between the two regulons. A neighbor joining tree (Figure 3) based on a distance matrix such defined was constructed using PHYLIP [49] and displayed by MEGA [41].

## Abbreviations

TU: transcription unit; BDBH: bidirectional best hit; DBD: DNA-binding domain; HTH: helix-turn-helix; LOR: log-odds ratio; MBIC11017: *Acaryochloris marina *MBIC11017; ATCC29413: *Anabaena variabilis *ATCC 29413; A-prime: *Synechococcus sp. *JA-3-3Ab; B-prime: *Synechococcus sp. *JA-2-3B'a(2-13); PCC8801: *Cyanothece sp*. PCC 8801; PCC7421:*Gloeobacter violaceus *PCC7421; NIES843: *Microcystis aeruginosa *NIES-843; PCC73102: *Nostoc punctiforme *PCC 73102; PCC7120: *Nostoc sp*. PCC 7120; CCMP1375: *Prochlorococcus marinus *CCMP1375; MED4: *Prochlorococcus marinus *MED4; AS9601: *Prochlorococcus marinus *AS9601; MIT9211: *Prochlorococcus marinus *MIT 9211; MIT9215: *Prochlorococcus marinus *MIT 9215; MIT9301: *Prochlorococcus marinus *MIT 9301; MIT9312: *Prochlorococcus marinus *MIT 9312; MIT9303: *Prochlorococcus marinus *MIT9303; MIT9313: *Prochlorococcus marinus *MIT9313; MIT9515: *Prochlorococcus marinus *MIT 9515; NATL1A: *Prochlorococcus marinus *NATL1A; NATL2A: *Prochlorococcus marinus *NATL2A; PCC7942: *Synechococcus elongatus *PCC 7942; PCC6301: *Synechococcus elongatus *PCC 6301; RCC307: *Synechococcus sp*. RCC307; WH7803: *Synechococcus sp*. WH 7803; WH8102: *Synechococcus sp*. WH8102; CC9605: *Synechococcus sp*. CC9605; CC9902: *Synechococcus sp*. CC9902; CC9311: *Synechococcus sp*. CC9311; PCC7002: *Synechococcus sp*. PCC 7002; PCC6803: *Synechocystis sp. *PCC 6803; BP-1: *Thermosynechococcus elongates *BP-1; and IMS101: *Trichodesmium erythraeum *IMS101.

## Authors' contributions

SL designed and conducted the experiment. MX helped conduct some analysis. ZS conceived the project. SL and ZS wrote the manuscript. All authors read and approved the final manuscript.

## Supplementary Material

Additional file 1 **Supplementary figures**. Additional file 1 contains one list: the putative LexA-binding sites found by MEME and BioProspector. Only the top1 motifs are included Click here for file

Additional file 2 **Supplementary figures**. Additional file 2 contains three figures. Figure S1: Phylogenetic tree of LexA-binding sites in cyanobacteria, *B. subtilis*, α-proteobacteria and *E. coli. *Figure S3: Results of genome-wide scanning for LexA-like binding sites in the five genomes that do not encode a *lexA *gene. Figure S4: Multiple sequence alignments of the full-length LexA in the 27 cyanobacterial genomes and *E. coli*. Click here for file

Additional file 3 **Supplementary figures**. Additional file 3 contains one figure. Figure S2: Phylogenetic tree of 183 LexA sequences from different bacteria domains. Click here for file

Additional file 4 **Supplementary tables**. Additional file 4 contains 26 tables (Table S1-26), the predicted LexA-binding sites and regulons in the 26 cyanobacterial genomes at *p *< 0.01. Click here for file

Additional file 5 **Supplementary tables**. Additional file 5 contains 5 tables: Table S27-31 containing the predicted LexA-binding sites in the five cyanobacterial genomes without a lexA gene at *p *< 0.01. Click here for file

Additional file 6 **Supplementary tables**. Additional file 6 contains 33 tables. Tables S32-S57 contains the predicted LexA-binding sites and regulons in the 26 cyanobacterial genomes harboring a lexA gene at *p *< 0.05. Table S58-62: predicted LexA-binding sites and regulons in the five cyanobacterial genomes without a *lexA *gene at *p *< 0.05. Table S63: list of the orthologs of the *E. coli *SOS response genes in 32 sequenced cyanobacteria. Table S64: list of the most conserved LexA regulon members (number of occurrence > = 6) in the 26 cyanobacterial genomes at *p *< 0.01. Click here for file
