# Supplementary material for: Computational analysis of LexA regulons in Cyanobacteria
Source: BMC Genomics. 2010 Sep 29;11:527. doi: 10.1186/1471-2164-11-527 (PMC3091678; doi:10.1186/1471-2164-11-527)
Supplement: Additional file 1 — Supplementary figures. Additional file 1 contains one list: the putative LexA-binding sites found by MEME and BioProspector. Only the top1 motifs are included [file 1471-2164-11-527-S1.DOC]

MEME MOTIF top 1 width = 14 sites = 45 llr = 486 E-value = 1.4e-026

Consensus: AGTACATATGTACT

Motif 1 sites sorted by position p-value

>Prochlorococcus_marinus_MIT_9312|78780039|-|PMT9312_1654||1541546|

AGTACAGATGTACT 1.47e-08

>Prochlorococcus_marinus_MIT_9301|126697091|recA|P9301_17531|1474905|

AGTACAGATGTACT 1.47e-08

>Prochlorococcus_marinus_MIT_9215|157414167|recA|P9215_18341|1571756|

AGTACAGATGTACT 1.47e-08

>Prochlorococcus_marinus_AS9601|123969301|recA|A9601_17691|1501246|

AGTACAGATGTACT 1.47e-08

>Prochlorococcus_marinus_MED4|33862118|recA|PMM1562|1490362|

AGTACACATGTACT 5.56e-08

>Nostoc_punctiforme_PCC_73102|186682248|-|Npun_F1842|2264642|

AGTACACCTGTACT 1.55e-07

>Nostoc_sp|17227584|-|alr0088|91454|

AGTACTTATGTACT 2.00e-07

>Anabaena_variabilis_ATCC_29413|75907684|-|Ava_1462|1803281|

AGTACTTATGTACT 2.00e-07

>Synechococcus_WH_7803|148238775|recA|SynWH7803_0439|451851|

CGTACATCTGTACT 2.60e-07

>Nostoc_punctiforme_PCC_73102|186685620 186685619|- -|Npun_R5568 Npun_R5567|6874286|

CGTACATTTGTACT 2.84e-07

>Prochlorococcus_marinus_MIT_9515|123966977|recA|P9515_17441|1531902|

AGTACGCATGTACT 8.63e-07

>Prochlorococcus_marinus_NATL2A|72382667|-|PMN2A_0828|784876|

AGGACGAATGTACT 1.26e-06

>Prochlorococcus_marinus_NATL1A|124026385|lexA|NATL1_16801|1369785|

AGGACAAATGTACT 1.26e-06

>Prochlorococcus_marinus_MIT_9211|159903846 159903845|lexA -|P9211_13051 P9211_13041| 1179390|

GGTACATATGTATT 1.26e-06

>Nostoc_sp|17232282 17232281|- -|all4790 all4789|5708604|

CGTACATTTGTACC 1.69e-06

>Anabaena_variabilis_ATCC_29413|75908280 75908279|- -|Ava_2059 Ava_2058 |2550363|

CGTACATTTGTACC 1.69e-06

>Prochlorococcus_marinus_NATL2A|72382971|recA|PMN2A_1133|1069540|

CGTACGTCTGTACT 2.43e-06

>Prochlorococcus_marinus_NATL1A|124026712|recA|NATL1_20071|1650513|

CGTACGTCTGTACT 2.43e-06

>Synechococcus_CC9902|78185048|-|Syncc9902_1481|1433703|

GGTACAAATGTATT 2.43e-06

>Synechococcus_CC9605|78212467|-|Syncc9605_0929|895147|

GGTACAAATGTATT 2.43e-06

>Prochlorococcus_marinus_MED4|33861818|lexA|PMM1262|1217007|

GGTACAAATGTATT 2.43e-06

>Synechococcus_CC9902|78185515|recA|Syncc9902_1949|1865147|

CGTACGTTTGTACT 2.61e-06

>Nostoc_sp|17232400|-|alr4908|5854261|

AGTACTAATGTTCT 3.34e-06

>Prochlorococcus_marinus_MIT_9303|124023614|lexA|P9303_19141|1682297|

GGTACACATGTATT 4.15e-06

>Prochlorococcus_marinus_MIT9313|33862653|-|PMT0380|429914|

GGTACACATGTATT 4.15e-06

>Nostoc_sp|17231208|uvrA|alr3716|strand:+|-|COG0178L|excinuclease ABC subunit A|4484828|

AGTACTATTGTTCT 6.45e-06

>Nostoc_punctiforme_PCC_73102|186686136 186686137 186686138|- - -|Npun_F6100 Npun_F6101 Npun_F6102|7546950|

AGTACGATTGTTCT 6.45e-06

>Anabaena_variabilis_ATCC_29413|75909796|-|Ava_3591| 4472591|

AGTTCTTCTGTATC 6.45e-06

>Cyanothece_PCC_8801|218246999 218246998|- -|PCC8801_2186 PCC8801_2185| 2262821|

AGTACTTATGTTCG 6.45e-06

>Synechococcus_sp_WH8102|33866594|recA|SYNW2062|1959344|

CGTACGCCTGTACT 7.08e-06

>Acaryochloris_marina_MBIC11017|158337075|lexA|AM1_3948|3998463|

AGTACAGGTGTTTT 7.54e-06

>Synechococcus_PCC_7002|170076752|ssb|SYNPCC7002_A0119|119541|

AGAACAGTTGTATG 1.75e-05

>Microcystis_aeruginosa_NIES_843|166364348|recA|MAE_16070|1445132|

CATACTGCTGTACT 1.75e-05

>Nostoc_sp|17230764|recA|all3272| 3946332|

AGTATATCTGTTCT 1.89e-05

>Nostoc_punctiforme_PCC_73102|186683193|-|Npun_F2914| 3601363|

AGTATATCTGTTCT 1.89e-05

>Anabaena_variabilis_ATCC_29413|75911121|recA|Ava_4925|6195124|

AGTATATCTGTTCT 1.89e-05

>Microcystis_aeruginosa_NIES_843|166366647|ssb|MAE_39060|3579563|

CATACTATTGTACT 2.05e-05

>Nostoc_sp|17232397|-|alr4905|5850231|

AGTTCTCATGTACT 2.34e-05

>Anabaena_variabilis_ATCC_29413|75908395|-|Ava_2176|2693530|

AGTTCTCATGTACT 2.34e-05

>Synechococcus_PCC_7002|170077054 170077053 170077052|recA - -|SYNPCC7002_A0426 SYNPCC7002_A0425 SYNPCC7002_A0424|448518|

AGTACGATTGAACT 2.34e-05

>Anabaena_variabilis_ATCC_29413|75908417|lexA|Ava_2198| 2727844|

AGTACTAATGTTCT 2.93e-05

>Prochlorococcus_marinus_CCMP1375|33241233|ssb|Pro1784|1637330|

AAAACATAAGTATT 3.27e-05

>Cyanothece_PCC_8801|218245807|-|PCC8801_0945|998592|

AAAACTCTTGTACT 3.48e-05

>Acaryochloris_marina_MBIC11017|158336681 158336682|- recA|AM1_3549 AM1_3550|3593106|

AATAAATCTGTACT 3.70e-05

>Synechococcus_RCC307|148242855|lexA|SynRCC307_1756|1537495|

GGCACAAATGTATT 6.08e-05

10 putative LexA boxes found by MEME not by BioProspector

**BioProspector motif 1**

**Motif #1: (AGTAC/GTACT)**

>Acaryochloris_marina_MBIC11017|158337075|lexA|AM1_3948|LexA repressor|3998463|

AGTACAGGTGTTTT

>Nostoc_sp|17232400|-|alr4908|5854261|

AGTACTAATGTTCT

>Prochlorococcus_marinus_MED4|33861818|lexA|PMM1262|1217007|

GGTACAAATGTATT

>Prochlorococcus_marinus_MIT9313|33862653|-|PMT0380|429914|

GGTACACATGTATT

>Prochlorococcus_marinus_MIT_9303|124023614|lexA|P9303_19141|1682297|

GGTACACATGTATT

>Prochlorococcus_marinus_NATL1A|124026385|lexA|NATL1_16801|1369769|

GGTACATATGTATT

>Prochlorococcus_marinus_NATL2A|72382667|-|PMN2A_0828|784860|

GGTACATATGTATT

>Synechococcus_CC9605|78212467|-|Syncc9605_0929|895147|

GGTACAAATGTATT

>Synechococcus_CC9902|78185048|-|Syncc9902_1481|1433703|

GGTACAAATGTATT

>Synechocystis_PCC6803|16330362|lexA|sll1626|1319721|

AGTCCTAGAGTCCT

>Anabaena_variabilis_ATCC_29413|75911121|recA|Ava_4925| 6195124|

AGTATATCTGTTCT

>Cyanothece_PCC_8801|218245807|-|PCC8801_0945| 998592|

AAAACTCTTGTACT

>Microcystis_aeruginosa_NIES_843|166364348|recA|MAE_16070|1445132|

CATACTGCTGTACT

>Nostoc_punctiforme_PCC_73102|186683193|-|Npun_F2914| 3601363|

AGTATATCTGTTCT

>Nostoc_sp|17230764|recA|all3272| 3946332|

AGTATATCTGTTCT

>Prochlorococcus_marinus_AS9601|123969301|recA|A9601_17691|1501246|

AGTACAGATGTACT

>Prochlorococcus_marinus_MED4|33862118|recA|PMM1562|1490362|

AGTACACATGTACT

>Prochlorococcus_marinus_MIT_9215|157414167|recA|P9215_18341|1571756|

AGTACAGATGTACT

>Prochlorococcus_marinus_MIT_9301|126697091|recA|P9301_17531|1474905|

AGTACAGATGTACT

>Synechococcus_CC9605|78211656|-|Syncc9605_0104|103815|

GATACCGCAGTTTA

>Prochlorococcus_marinus_MIT_9312|78780039|-|PMT9312_1654|1541546|

AGTACAGATGTACT

>Prochlorococcus_marinus_MIT_9515|123966977|recA|P9515_17441|1531902|

AGTACGCATGTACT

>Prochlorococcus_marinus_NATL1A|124026712|recA|NATL1_20071|1650513|

CGTACGTCTGTACT

>Prochlorococcus_marinus_NATL2A|72382971|recA|PMN2A_1133|1069540|

CGTACGTCTGTACT

>Synechococcus_CC9902|78185515|recA|Syncc9902_1949|1865147|

CGTACGTTTGTACT

>Synechococcus_WH_7803|148238775|recA|SynWH7803_0439|451851|

CGTACATCTGTACT

>Synechococcus_sp_WH8102|33866594|recA|SYNW2062| 1959344|

CGTACGCCTGTACT

>Anabaena_variabilis_ATCC_29413|75909796|-|Ava_3591|4472814|

AGTACGATTGTTCT

>Nostoc_sp|17231208|uvrA|alr3716|4484828|

AGTACTATTGTTCT

>Anabaena_variabilis_ATCC_29413|75907684|-|Ava_1462|1803281|

AGTACTTATGTACT

>Microcystis_aeruginosa_NIES_843|166366647|ssb|MAE_39060|3579563|

CATACTATTGTACT

>Prochlorococcus_marinus_MIT_9515|123967045|-|P9515_18121| 1596455|

AATATATCTATTCT

>Nostoc_punctiforme_PCC_73102|186682248|-|Npun_F1842|2264642|

AGTACACCTGTACT

>Nostoc_sp|17227584|-|alr0088| 91454|

AGTACTTATGTACT

>Prochlorococcus_marinus_CCMP1375|33241233|ssb|Pro1784| 1637330|

AAAACATAAGTATT

>Synechococcus_PCC_7002|170076752|ssb|SYNPCC7002_A0119|119541|

AGAACAGTTGTATG

>Anabaena_variabilis_ATCC_29413|75908395|-|Ava_2176|2693530|

AGTTCTCATGTACT

>Nostoc_sp|17232397|-|alr4905| 5850231|

AGTTCTCATGTACT

>Synechococcus_WH_7803|148238507|ssb|SynWH7803_0171| 190227|

CAACCGTCAGTTCT

4 putative LexA boxes found by BioProspector not by MEME
