# Supplementary material for: Computational analysis of LexA regulons in Cyanobacteria
Source: BMC Genomics. 2010 Sep 29;11:527. doi: 10.1186/1471-2164-11-527 (PMC3091678; doi:10.1186/1471-2164-11-527)
Supplement: Additional file 2 — Supplementary figures. Additional file 2 contains three figures. Figure S1: Phylogenetic tree of LexA-binding sites in cyanobacteria, B. subtilis, α-proteobacteria and E. coli. Figure S3: Results of genome-wide scanning for LexA-like binding sites in the five genomes that do not encode a lexA gene. Figure S4: Multiple sequence alignments of the full-length LexA in the 27 cyanobacterial genomes and E. coli. [file 1471-2164-11-527-S2.PPT]

## Slide 1
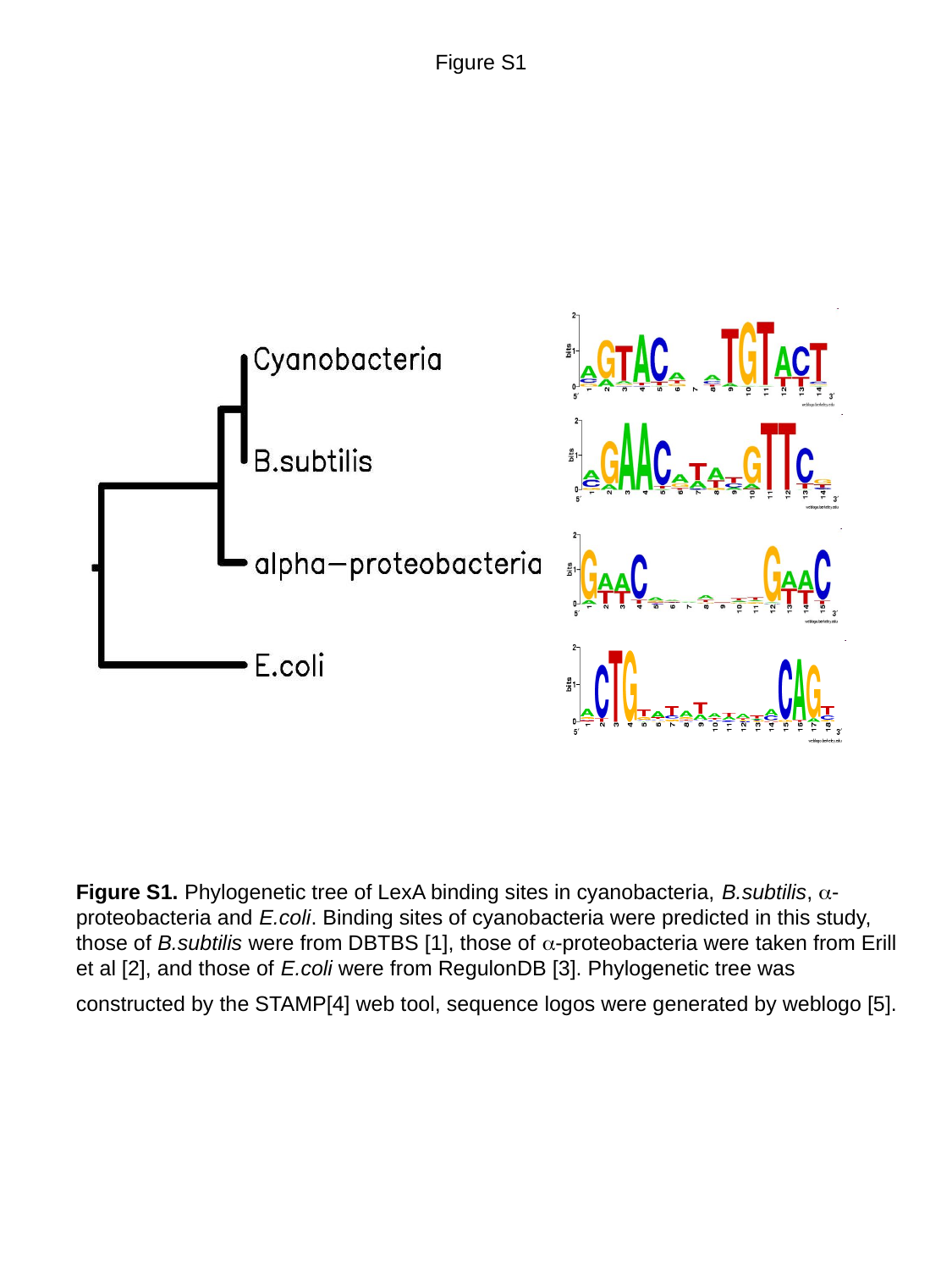

Figure S1
Figure S1. Phylogenetic tree of LexA binding sites in cyanobacteria, B.subtilis, α-proteobacteria and E.coli. Binding sites of cyanobacteria were predicted in this study, those of B.subtilis were from DBTBS [1], those of α-proteobacteria were taken from Erill et al [2], and those of E.coli were from RegulonDB [3]. Phylogenetic tree was constructed by the STAMP[4] web tool, sequence logos were generated by weblogo [5].

## Slide 2
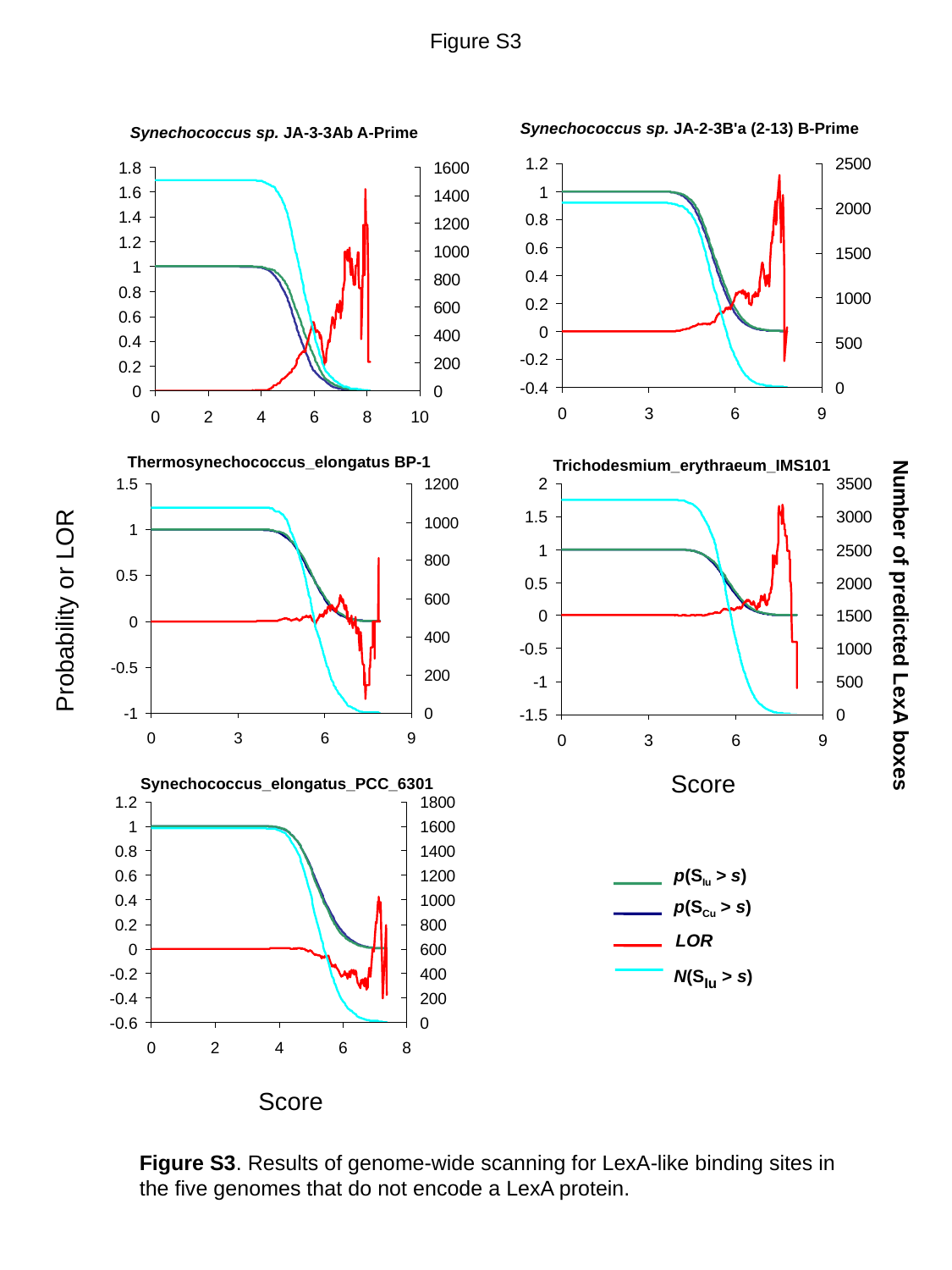

Figure S3
Synechococcus sp. JA-2-3B'a (2-13) B-Prime
Synechococcus sp. JA-3-3Ab A-Prime
Thermosynechococcus_elongatus BP-1
Trichodesmium_erythraeum_IMS101
Probability or LOR
Number of predicted LexA boxes
Score
Synechococcus_elongatus_PCC_6301
p(SIu > s)
p(SCu > s)
LOR
N(SIu > s)
Score
Figure S3. Results of genome-wide scanning for LexA-like binding sites in the five genomes that do not encode a LexA protein.

## Slide 3
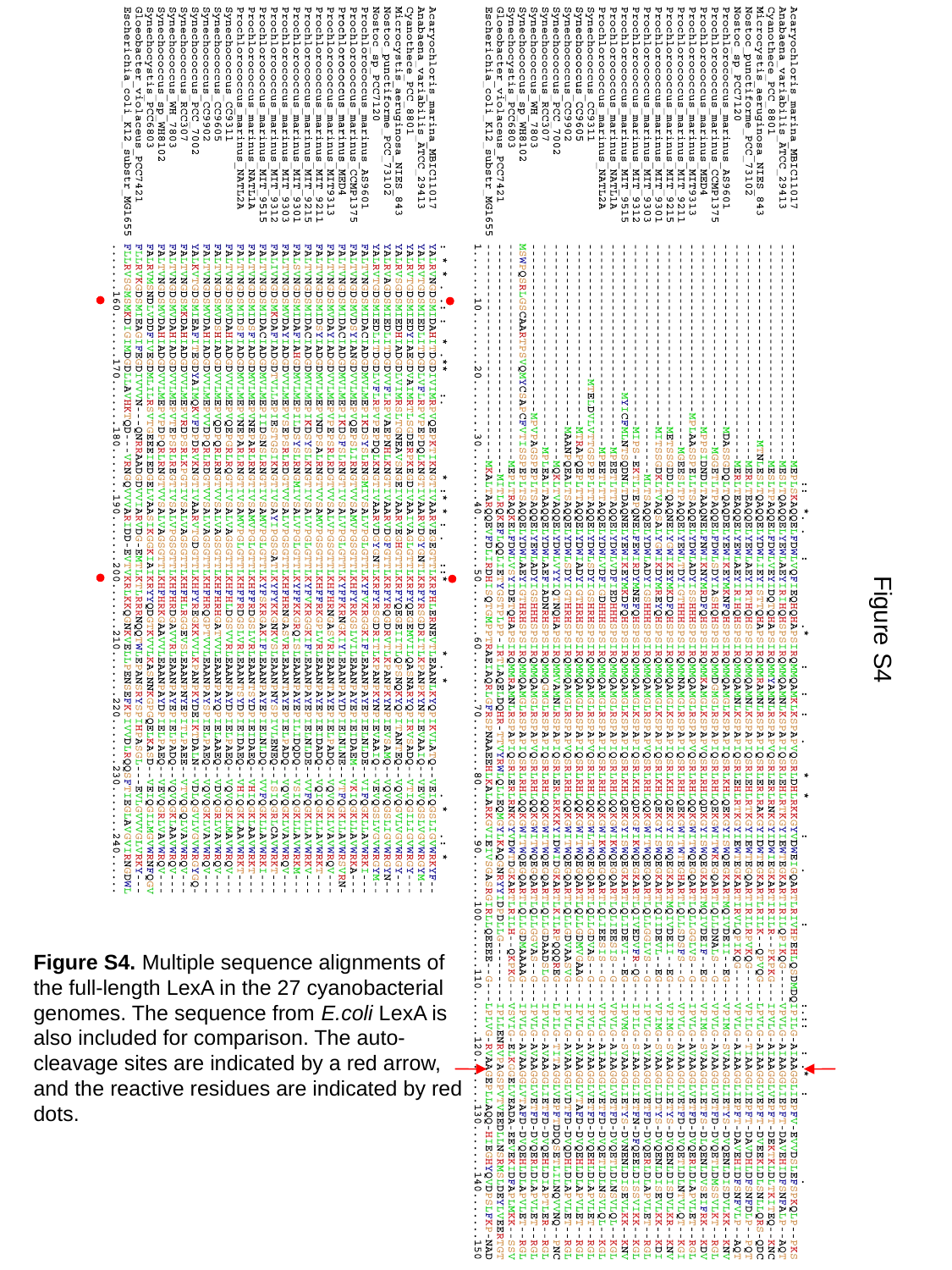

Figure S4
Figure S4. Multiple sequence alignments of the full-length LexA in the 27 cyanobacterial genomes. The sequence from E.coli LexA is also included for comparison. The auto-cleavage sites are indicated by a red arrow, and the reactive residues are indicated by red dots.

## Slide 4
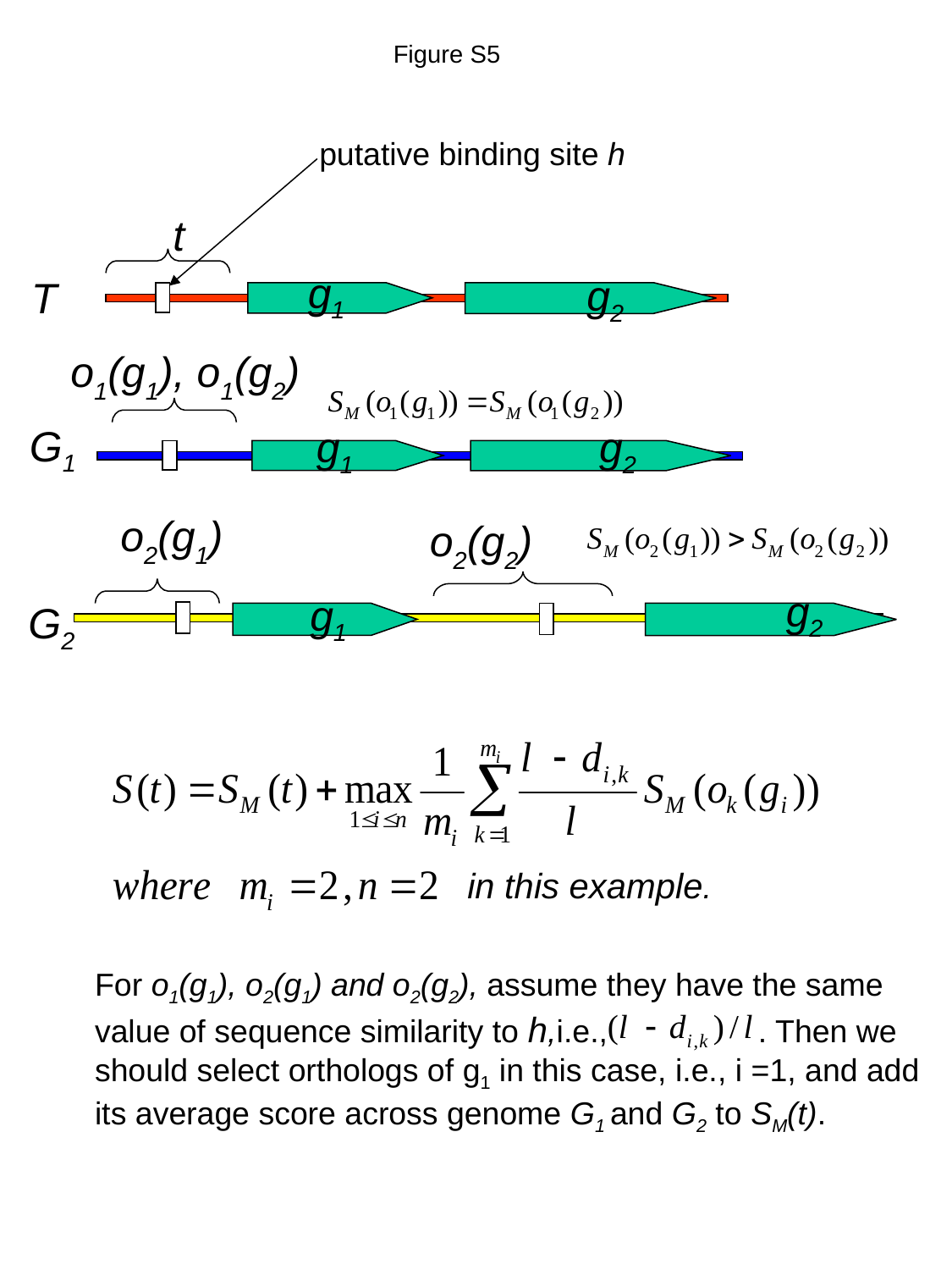

Figure S5
putative binding site h
t
g1
g2
T
o1(g1), o1(g2)
G1
g1
g2
o2(g1)
o2(g2)
g2
g1
G2
in this example.
For o1(g1), o2(g1) and o2(g2), assume they have the same
value of sequence similarity to h,i.e., . Then we
should select orthologs of g1 in this case, i.e., i =1, and add
its average score across genome G1 and G2 to SM(t).

## Slide 5
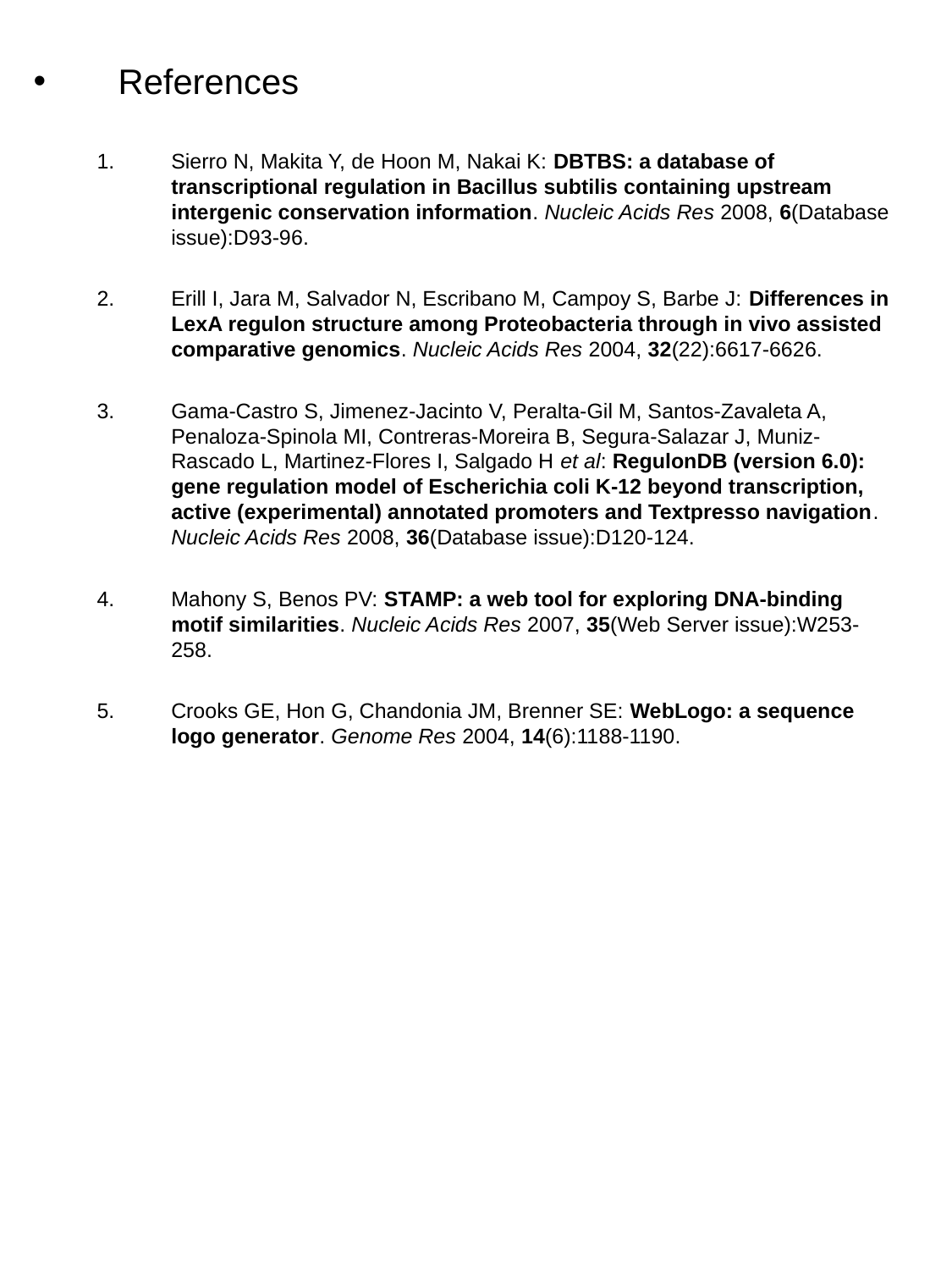

# References
Sierro N, Makita Y, de Hoon M, Nakai K: DBTBS: a database of transcriptional regulation in Bacillus subtilis containing upstream intergenic conservation information. Nucleic Acids Res 2008, 6(Database issue):D93-96.
Erill I, Jara M, Salvador N, Escribano M, Campoy S, Barbe J: Differences in LexA regulon structure among Proteobacteria through in vivo assisted comparative genomics. Nucleic Acids Res 2004, 32(22):6617-6626.
Gama-Castro S, Jimenez-Jacinto V, Peralta-Gil M, Santos-Zavaleta A, Penaloza-Spinola MI, Contreras-Moreira B, Segura-Salazar J, Muniz-Rascado L, Martinez-Flores I, Salgado H et al: RegulonDB (version 6.0): gene regulation model of Escherichia coli K-12 beyond transcription, active (experimental) annotated promoters and Textpresso navigation. Nucleic Acids Res 2008, 36(Database issue):D120-124.
Mahony S, Benos PV: STAMP: a web tool for exploring DNA-binding motif similarities. Nucleic Acids Res 2007, 35(Web Server issue):W253-258.
Crooks GE, Hon G, Chandonia JM, Brenner SE: WebLogo: a sequence logo generator. Genome Res 2004, 14(6):1188-1190.
