# Supplementary material for: Computational analysis of LexA regulons in Cyanobacteria
Source: BMC Genomics. 2010 Sep 29;11:527. doi: 10.1186/1471-2164-11-527 (PMC3091678; doi:10.1186/1471-2164-11-527)
Supplement: Additional file 3 — Supplementary figures. Additional file 3 contains one figure. Figure S2: Phylogenetic tree of 183 LexA sequences from different bacteria domains. [file 1471-2164-11-527-S3.PDF]

Figure S2

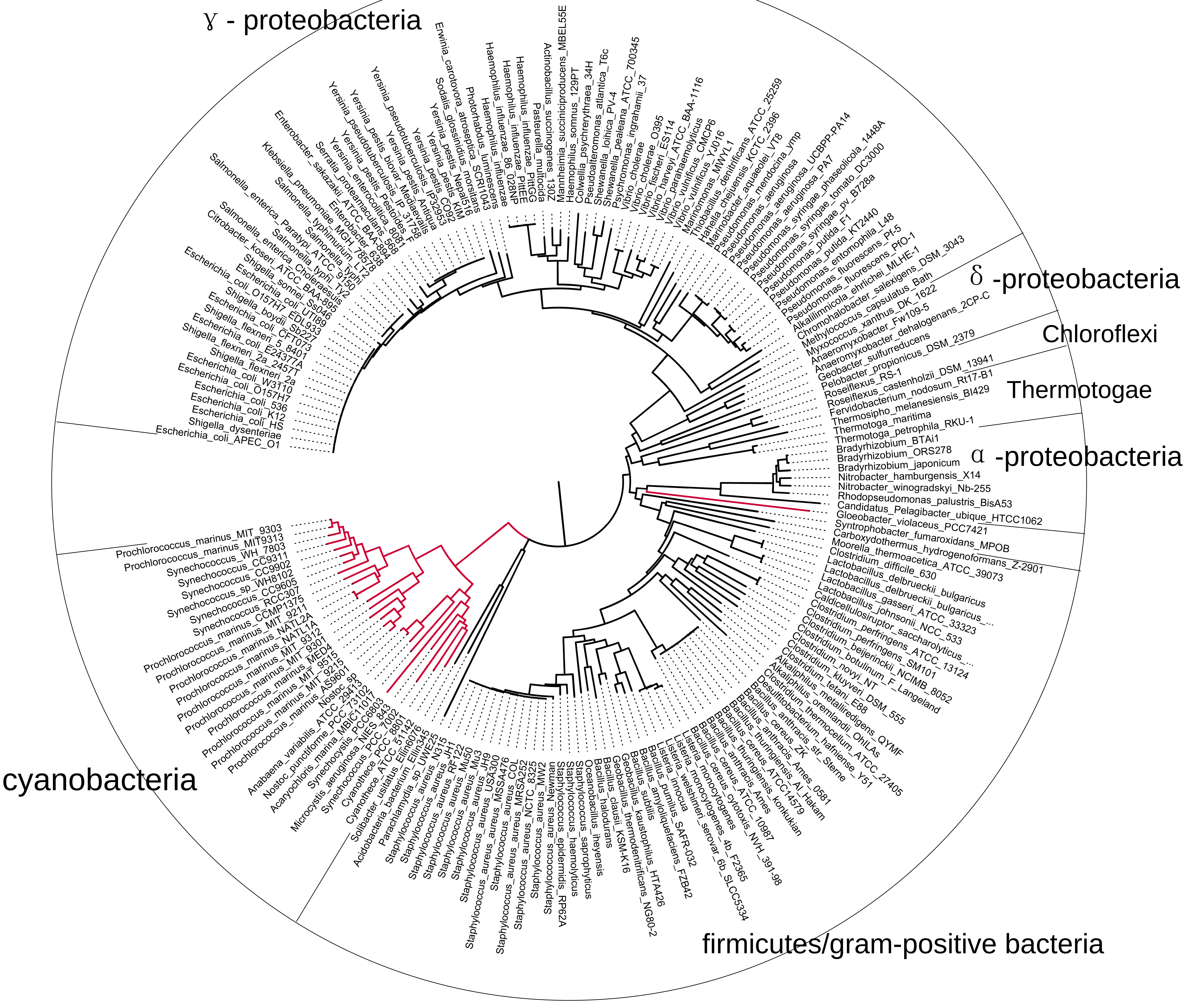

Figure S2. Phylogenetic tree of LexA sequences across a total of 183 cyanobacteria, gram-positive bacteria, α-proteobacteria, δ-proteobacteria, γ-proteobacteria and other bacterial species/strains. The tree was constructed in MEGA. Branches of cyanobacteria are colored in red.
