# Supplementary material for: Computational analysis of LexA regulons in Cyanobacteria
Source: BMC Genomics. 2010 Sep 29;11:527. doi: 10.1186/1471-2164-11-527 (PMC3091678; doi:10.1186/1471-2164-11-527)
Supplement: Additional file 4 — Supplementary tables. Additional file 4 contains 26 tables (Table S1-26), the predicted LexA-binding sites and regulons in the 26 cyanobacterial genomes at p < 0.01. [file 1471-2164-11-527-S4.DOC]

**Table S1**. Predicted LexA binding sites in *Acaryochloris marina* MBIC11017 genome at *P* < 0.01

| **Rank** | **Transcription Unit** | **Name** | **Putative LexA Binding Site** | **Position** | **Score** | **p-value** | **Have an orthologue or not(Y/N)** |
| --- | --- | --- | --- | --- | --- | --- | --- |
| 1 | AM1_2434 AM1_2433 AM1_2432 | - - - | AGTACATTTGTACT | -27 | 8.468547 | 2.96E-05 | Y |
| 2 | AM1_5804 | - | AGAACATATGTACT | -89 | 8.246821 | 5.48E-05 | Y |
| 3 | AM1_2528 AM1_2529 | umuD umuC | AGTACATATGCACT | -194 | 8.042541 | 0.000169 | Y |
| 4 | AM1_5483 | recA | AGTACAAGTATACT | -38 | 7.93748 | 0.000272 | Y |
| 5 | AM1_2966 | - | AGTACAAATGTACT | -180 | 7.8676 | 0.000426 | N |
| 6 | AM1_3948 | lexA | AGTACAGGTGTTTT | -92 | 7.839691 | 0.000504 | Y |
| 7 | AM1_4792 | - | AGTACAAAAGTACT | -291 | 7.835694 | 0.000504 | Y |
| 9 | AM1_2965 | - | AGTACATTTGTACT | -32 | 7.816 | 0.000579 | N |
| 10 | AM1_3075 | - | TGTACAGATGGACT | -183 | 7.747911 | 0.000782 | Y |
| 11 | AM1_5805 AM1_5803 | - - | CGTACAAGTGTCAT | -57 | 7.528477 | 0.001537 | Y |
| 12 | AM1_2440 | dnaK | AGTACAAATGAACT | -88 | 7.507442 | 0.001636 | Y |
| 13 | AM1_2438 | - | AGTTCATTTGTACT | -392 | 7.477934 | 0.001788 | Y |
| 14 | AM1_5484 AM1_5485 AM1_5486 AM1_5487 | - - dinB - | AGTATACTTGTACT | -59 | 7.451674 | 0.001922 | Y |
| 15 | AM1_0325 AM1_0326 | - - | GGTACATCTGAAGT | -355 | 7.408807 | 0.002343 | Y |
| 16 | AM1_4305 | - | AGAACATCTGTCCT | -95 | 7.406198 | 0.002343 | Y |
| 17 | AM1_5017 | - | AGTATATATGTACT | -104 | 7.381936 | 0.002499 | Y |
| 18 | AM1_3857 AM1_3856 | - - | AGTACTCTCGTTCT | -35 | 7.364162 | 0.002661 | Y |
| 19 | AM1_3965 | nadB | TGTACAATTGCACT | -85 | 7.333832 | 0.002945 | Y |
| 20 | AM1_4624 | - | GGTACCTCTGTTTT | -262 | 7.322071 | 0.003045 | Y |
| 21 | AM1_3214 | groL | AGGACGCTTGTAAT | -1 | 7.306734 | 0.003239 | Y |
| 22 | AM1_1600 | - | TGTACACCTGTCCT | -134 | 7.2784 | 0.003553 | N |
| 23 | AM1_2953 AM1_2954 AM1_2955 | - - - | AGTACAACCGTGCT | -17 | 7.265397 | 0.003676 | Y |
| 24 | AM1_1190 AM1_1191 | - por | AGTATATTTGTACT | -5 | 7.253744 | 0.003786 | Y |
| 25 | AM1_2750 AM1_2751 | - - | TGTACGTAAGTATT | -10 | 7.25215 | 0.003786 | Y |
| 26 | AM1_5198 | - | AGTACTCGTGCAGT | 7 | 7.245818 | 0.003898 | Y |
| 27 | AM1_5029 AM1_5028 | - - | AGTATACTTGTACT | -350 | 7.242675 | 0.003898 | Y |
| 28 | AM1_5727 | - | AGTACTGATGTAGG | -251 | 7.241957 | 0.003898 | Y |
| 29 | AM1_4180 AM1_4181 | - - | GATACACTTGTACT | -121 | 7.241871 | 0.003898 | Y |
| 30 | AM1_1133 | - | TGTACAGCTGTAAT | -176 | 7.1962 | 0.004734 | N |
| 31 | AM1_2119 | - | GGTACTCCTGTTCC | 23 | 7.178616 | 0.005157 | Y |
| 32 | AM1_6350 | - | TGTACCCCTGTCCT | -141 | 7.178 | 0.005157 | N |
| 33 | AM1_1189 AM1_1188 | - - | AGTACAAATATACT | -17 | 7.176579 | 0.005157 | Y |
| 34 | AM1_5737 | gyrA | AGTACAAGTGCATT | -93 | 7.166203 | 0.005366 | Y |
| 35 | AM1_4457 AM1_4458 AM1_4459 | - thyX - | GGCACTCATGTAGT | -350 | 7.165485 | 0.005366 | Y |
| 36 | AM1_0938 | - | CGAACCTTTGTAAT | -299 | 7.14342 | 0.006057 | Y |
| 37 | AM1_3500 | - | ACTACATTTGTATT | -345 | 7.140351 | 0.006057 | Y |
| 38 | AM1_4418 | - | ATTACATTTGTGCT | -74 | 7.137369 | 0.006358 | Y |
| 39 | AM1_0381 AM1_0380 AM1_0379 AM1_0378 AM1_0377 | - - - - - | TCTACATCTGTAGG | -642 | 7.105809 | 0.007091 | Y |
| 40 | AM1_1271 | - | AGCACAGAGGTATT | -474 | 7.080716 | 0.007641 | Y |
| 41 | AM1_0205 AM1_0204 AM1_0203 AM1_0202 AM1_0201 | - - - - - | AGGACTTTTGTCTT | -419 | 7.060935 | 0.008176 | Y |
| 42 | AM1_6286 AM1_6285 AM1_6284 AM1_6283 AM1_6282 | - - - - - | AGTACCACTGTGAA | -745 | 7.056329 | 0.008488 | Y |
| 43 | AM1_3409 | - | TCTACTGCTGTAGT | 15 | 7.050685 | 0.008488 | Y |
| 44 | AM1_2133 AM1_2134 | - - | AGTACGTTTGGATT | -352 | 7.042883 | 0.008816 | Y |
| 45 | AM1_5978 | - | AGTGCCATTGTTCT | -126 | 7.040665 | 0.008816 | Y |
| 46 | AM1_0287 | - | ACTACGTTTGTAGT | -169 | 7.039806 | 0.009174 | Y |
| 47 | AM1_1741 AM1_1740 AM1_1739 AM1_1737 | - - - folC | AGAACTTTTGTTAT | -107 | 7.029097 | 0.00948 | Y |
| 48 | AM1_0386 | - | GGTACTCAAGTATT | -413 | 7.024204 | 0.00948 | Y |

**Table S2**. Predicted LexA binding sites in *Anabaena variabilis* ATCC29413 genome at *P* < 0.01

| **Rank** | **Transcription Unit** | **Name** | **Putative LexA Binding Site** | **Position** | **Score** | **p-value** | **Have an orthologue or not(Y/N)** |
| --- | --- | --- | --- | --- | --- | --- | --- |
| 1 | Ava_1060 | - | AGTACTTATGTACT | -173 | 9.77E+00 | 0 | Y |
| 2 | Ava_3750 | - | AGTACAATTGTACT | -50 | 9.50E+00 | 0 | Y |
| 3 | Ava_4301 | - | AGTACAATTGTACT | -62 | 9.284348 | 0 | Y |
| 4 | Ava_4578 Ava_4579 | - - | CGTACAAATGTACT | -410 | 8.900224 | 0 | Y |
| 5 | Ava_2066 | - | AGTACACTTGTACT | -264 | 8.62188 | 5.88E-06 | Y |
| 6 | Ava_3370 | - | AGTACTTTTGTACT | -194 | 8.591664 | 1.01E-05 | Y |
| 7 | Ava_1462 | - | AGTACTTATGTACT | -16 | 8.522075 | 1.60E-05 | Y |
| 8 | Ava_3837 | - | TGTACCAGTGTCAT | -456 | 8.462 | 2.10E-05 | Y |
| 9 | Ava_1061 | - | AGTACATAAGTACT | -47 | 8.448338 | 2.60E-05 | Y |
| 10 | Ava_2695 | - | AGTACAATTGCACT | -119 | 8.440845 | 2.60E-05 | Y |
| 11 | Ava_2198 | - | AGTACTAATGTTCT | -35 | 8.384681 | 4.20E-05 | Y |
| 12 | Ava_3961 | - | AGTACTTCCGTACT | -42 | 8.304401 | 6.97E-05 | Y |
| 13 | Ava_2841 | - | AGTACTAATGTACC | -429 | 8.295435 | 7.39E-05 | Y |
| 14 | Ava_3235 Ava_3236 Ava_3237 | - - - | CGAACTGATGTACT | -26 | 8.188593 | 0.000119 | Y |
| 15 | Ava_0011 | - | GGTACCTTCGTACT | -133 | 8.182932 | 0.000119 | Y |
| 16 | Ava_3591 | - | AGTACGATTGTTCT | -72 | 8.174385 | 0.00012 | Y |
| 17 | Ava_4928 Ava_4927 | - - | TGTACTGGTGTAGC | -73 | 8.162125 | 0.000126 | Y |
| 18 | Ava_2059 | - | CGTACATTTGTACC | -31 | 8.159512 | 0.000132 | Y |
| 19 | Ava_4998 | - | CATACAACTGTACT | 22 | 8.05765 | 0.000204 | Y |
| 20 | Ava_3034 | - | AGGACTTATGTACT | -354 | 8.026879 | 0.000255 | Y |
| 21 | Ava_4938 | - | GGTACCTCTGTAAC | -208 | 7.962821 | 0.000391 | Y |
| 22 | Ava_2176 | - | AGTTCTCATGTACT | -104 | 7.927492 | 0.000491 | Y |
| 23 | Ava_2100 Ava_2099 | - - | AGTACGTCTGTATT | -196 | 7.919857 | 0.000511 | Y |
| 24 | Ava_2034 | - | AATACAACTGTAAT | -226 | 7.9186 | 0.000511 | Y |
| 25 | Ava_4459 | - | AATACAAGTGTAGT | -25 | 7.9023 | 0.000531 | Y |
| 26 | Ava_0560 | - | AGTACCTGAGTCCT | -243 | 7.880005 | 0.000585 | Y |
| 27 | Ava_1777 | - | GGTACTTCTGTGAT | 5 | 7.865292 | 0.00066 | Y |
| 28 | Ava_1710 | - | AGTACAGATGCACT | -595 | 7.865 | 0.00066 | Y |
| 29 | Ava_0070 Ava_0069 | - - | CGTACTTTTGTCAT | -152 | 7.863822 | 0.00066 | Y |
| 30 | Ava_4577 | - | AGTACATTTGTACG | -87 | 7.849153 | 0.000736 | Y |
| 31 | Ava_0864 | - | AGTACTAATGTTTT | -264 | 7.837883 | 0.000785 | Y |
| 32 | Ava_1709 | - | AGTGCATCTGTACT | -38 | 7.821444 | 0.000817 | Y |
| 33 | Ava_1048 | - | TGTACGTGTGTACA | -104 | 7.797948 | 0.000952 | Y |
| 34 | Ava_4067 | - | AAAACTGATGTACT | 36 | 7.7946 | 0.000952 | Y |
| 35 | Ava_4495 Ava_4496 | - - | ATTACTCATGTACA | 30 | 7.785636 | 0.001003 | Y |
| 36 | Ava_2809 Ava_2810 Ava_2811 | - - - | AGTACCGATGTATG | -448 | 7.773846 | 0.001054 | Y |
| 37 | Ava_3697 | - | AGTACTTAAGTACT | -163 | 7.771898 | 0.001054 | Y |
| 38 | Ava_1188 Ava_1189 | - - | TGTACCTTTGTAGT | -56 | 7.771144 | 0.001054 | Y |
| 39 | Ava_2170 | - | TGCACACCTGTGCA | 28 | 7.755061 | 0.001166 | Y |
| 40 | Ava_4997 | - | ATTACATTTGTACT | -49 | 7.7512 | 0.001166 | Y |
| 41 | Ava_1956 | - | GGTACTGCTGTGGC | 34 | 7.748917 | 0.001236 | Y |
| 42 | Ava_4031 Ava_4030 | - - | CGTACCATTGTTTT | -103 | 7.744291 | 0.001236 | Y |
| 43 | Ava_1272 | - | TGAACTTTTGTACT | -53 | 7.743025 | 0.001236 | Y |
| 44 | Ava_1767 Ava_1766 | - - | AGTGCAACTGTACT | -30 | 7.739453 | 0.001292 | Y |
| 45 | Ava_3390 | - | AGTACAGAGGTTCT | -556 | 7.729114 | 0.00136 | Y |
| 46 | Ava_4694 | - | GTTACTTATGTCCT | -75 | 7.726902 | 0.00136 | Y |
| 47 | Ava_3384 | - | AGTACAGGTGTCAT | -33 | 7.707734 | 0.001507 | Y |
| 48 | Ava_3234 | - | GGTACTTTTGTGTT | -89 | 7.704073 | 0.001507 | Y |
| 49 | Ava_1735 | - | ATTACCCCTGTACT | -81 | 7.70222 | 0.001507 | Y |
| 50 | Ava_4458 | - | ACTACACTTGTATT | -32 | 7.680655 | 0.001657 | Y |
| 51 | Ava_2609 | - | CGGACCCATGTACT | -36 | 7.678874 | 0.001735 | Y |
| 52 | Ava_3486 Ava_3487 Ava_3488 Ava_3489 | - - - - | AGTACGATTGTACC | -37 | 7.673002 | 0.001735 | Y |
| 53 | Ava_3431 | - | AGTACTAGTGTTTT | -172 | 7.665812 | 0.001812 | Y |
| 54 | Ava_2918 | - | TGTACCCTTGTACC | -132 | 7.636282 | 0.002135 | Y |
| 55 | Ava_4148 Ava_4149 | - - | AGTTCAAATGTACT | -229 | 7.620272 | 0.002259 | Y |
| 56 | Ava_3610 | - | AGTACAGATGTCTT | -233 | 7.596393 | 0.002643 | Y |
| 57 | Ava_1209 | - | TGTTCTGATGTGCT | -328 | 7.595789 | 0.002643 | Y |
| 58 | Ava_1962 | - | AGTACAAGTGTTCA | -84 | 7.582118 | 0.002781 | Y |
| 59 | Ava_4618 | - | TGTACCCTTGTTTT | -671 | 7.578743 | 0.002932 | Y |
| 60 | Ava_3671 Ava_3672 Ava_3673 Ava_3674 | - - - - | TGTACCTCTGTCTT | -205 | 7.575688 | 0.002932 | Y |
| 61 | Ava_4995 | - | AGCACTAATGTAGT | -47 | 7.55893 | 0.003231 | Y |
| 62 | Ava_5064 | - | CGTGCATTTGTCCT | -53 | 7.546566 | 0.003362 | Y |
| 63 | Ava_0920 | - | ATTACAATTGTTCT | -8 | 7.512388 | 0.003863 | Y |
| 64 | Ava_0008 | - | TTTACGATTGTGCT | -58 | 7.506844 | 0.004006 | Y |
| 65 | Ava_3553 | - | TGTACATCTGTTTT | -155 | 7.493705 | 0.004164 | Y |
| 66 | Ava_1522 | - | AGTACGTAAGTCCT | -3 | 7.487206 | 0.004367 | Y |
| 67 | Ava_0870 | - | TGTTCTGATGTACT | -122 | 7.476889 | 0.004552 | Y |
| 68 | Ava_1183 | - | TTTACGACTGTACT | -552 | 7.473209 | 0.004552 | Y |
| 69 | Ava_0603 Ava_0604 | - - | AGTAATTATGTACT | -55 | 7.457459 | 0.004916 | Y |
| 70 | Ava_1553 | - | GGAACTTTTGTTAT | -139 | 7.446046 | 0.005137 | Y |
| 71 | Ava_4895 | - | CGTACTTATGATAT | -355 | 7.4433 | 0.005137 | Y |
| 72 | Ava_4121 | - | AGTACCATTGTTCA | 17 | 7.442129 | 0.005137 | Y |
| 73 | Ava_4007 | - | TGTACATTTGTTCG | -86 | 7.435629 | 0.005345 | Y |
| 74 | Ava_2071 | - | AGTACTATTGCAGT | -44 | 7.428463 | 0.005553 | Y |
| 75 | Ava_0496 | - | GGTACAAATGTATG | -136 | 7.427922 | 0.005553 | Y |
| 76 | Ava_2412 | - | ACTACTCTTGTACT | -44 | 7.425228 | 0.005553 | Y |
| 77 | Ava_3892 Ava_3891 | - - | CGTACAAATGTAAG | -211 | 7.423048 | 0.005553 | Y |
| 78 | Ava_0150 | - | CGTGCTGCTGTTCC | 20 | 7.417125 | 0.00579 | Y |
| 79 | Ava_1693 Ava_1692 Ava_1691 | - - - | CGTATATTTGTAAT | -23 | 7.414325 | 0.00579 | Y |
| 80 | Ava_4680 | - | AGTACAGATGTATG | 1 | 7.408111 | 0.006036 | Y |
| 81 | Ava_4925 | recA | AGTATATCTGTTCT | -53 | 7.398027 | 0.006286 | Y |
| 82 | Ava_1835 | - | AGTACCAAAGTTCT | -34 | 7.396492 | 0.006286 | Y |
| 83 | Ava_4820 | - | GCGACAGTTGTACT | 29 | 7.395491 | 0.006286 | Y |
| 84 | Ava_0609 | - | TGTACTGCTGTGAT | -576 | 7.3846 | 0.006557 | Y |
| 85 | Ava_1782 | - | TGTGCATCTGTATT | 28 | 7.38389 | 0.006557 | Y |
| 86 | Ava_3868 | - | CGTACAAGCGTACA | -86 | 7.371248 | 0.006852 | Y |
| 87 | Ava_2822 | - | GGTTCCTATGTATT | -280 | 7.361952 | 0.007139 | Y |
| 88 | Ava_3791 | - | TGTACAATTGTCAG | -164 | 7.361621 | 0.007139 | Y |
| 89 | Ava_2652 | - | GGATCGAGTGTTCT | -471 | 7.352325 | 0.00741 | Y |
| 90 | Ava_2163 Ava_2164 | - - | TGTACGTAAGTCCT | -40 | 7.343768 | 0.007682 | Y |
| 91 | Ava_4727 | - | AGCACCAATGTCCA | -469 | 7.343171 | 0.007682 | Y |
| 92 | Ava_2459 | - | GGTACGTAAGTCCT | -136 | 7.342907 | 0.007682 | Y |
| 93 | Ava_1456 | - | TCTACTGCTGTAAT | -47 | 7.3423 | 0.007682 | Y |
| 94 | Ava_3364 | - | TGTACCCCTGTAGC | -179 | 7.33728 | 0.007954 | Y |
| 95 | Ava_2381 | - | CGGACTTTTGTACA | 32 | 7.32434 | 0.00826 | Y |
| 96 | Ava_2432 | - | AGTACAAATGAACA | 5 | 7.322433 | 0.00826 | Y |
| 97 | Ava_3746 | - | CGTACTCAAGTAGT | -46 | 7.319991 | 0.008562 | Y |
| 98 | Ava_3311 | - | TGTACATTTGTAAG | -390 | 7.319517 | 0.008562 | Y |
| 99 | Ava_4346 | hisG | AGAACGGGTGTAAT | -311 | 7.316563 | 0.008562 | Y |
| 100 | Ava_2109 | - | ATTACAAATGTATA | -94 | 7.3041 | 0.008884 | Y |
| 101 | Ava_2383 | - | ATTACAAATGAACT | -115 | 7.29998 | 0.009215 | Y |
| 102 | Ava_0366 | - | TGAACGGATGTCAT | -117 | 7.297427 | 0.009215 | Y |
| 103 | Ava_2069 | - | TGTGCATTTGTGCT | -156 | 7.28934 | 0.009534 | Y |
| 104 | Ava_2799 | - | AGTACATCTGTGGC | -256 | 7.285008 | 0.009534 | Y |
| 105 | Ava_1763 | - | TGAACTTTAGTACT | 13 | 7.283217 | 0.009534 | Y |
| 106 | Ava_3175 | - | GGTACGAGGGTTTT | -260 | 7.282406 | 0.009534 | Y |
| 107 | Ava_3729 | - | AGTACAACTGAGGT | -3 | 7.281977 | 0.009534 | Y |

**Table S3**. Predicted LexA binding sites in *Cyanothece* PCC 8801 genome at *P* < 0.01

| **Rank** | **Transcription Unit** | **Name** | **Putative LexA Binding Site** | **Position** | **Score** | **p-value** | **Have an orthologue or not(Y/N)** |
| --- | --- | --- | --- | --- | --- | --- | --- |
| 1 | PCC8801_4135 PCC8801_4136 | - - | TGTACCATTGTCCT | -61 | 8.027755 | 0.00011 | Y |
| 2 | PCC8801_0384 | - | CGTACTTCTGTGCT | 29 | 7.934106 | 0.000232 | Y |
| 3 | PCC8801_2186 | - | AGTACTTATGTTCG | -61 | 7.756999 | 0.000627 | Y |
| 4 | PCC8801_1259 | - | ACTACACTTGTATT | -35 | 7.662255 | 0.000912 | Y |
| 5 | PCC8801_1923 PCC8801_1922 PCC8801_1921 | - - - | AGGACTGCTGTATT | -120 | 7.62E+00 | 0.001095 | Y |
| 6 | PCC8801_0605 | - | AGTACCCTTGTGGT | -41 | 7.55E+00 | 0.001531 | Y |
| 7 | PCC8801_2187 | - | TGTACTATTGTGTT | -45 | 7.54E+00 | 0.001577 | Y |
| 8 | PCC8801_1523 PCC8801_1524 | - - | AGTACGATTGTCCC | -180 | 7.53E+00 | 0.001657 | Y |
| 9 | PCC8801_4113 | - | GGTACACTTGTGTT | -93 | 7.52E+00 | 0.00172 | Y |
| 10 | PCC8801_2405 | - | AATACAATTGTACT | -376 | 7.48E+00 | 0.001957 | Y |
| 11 | PCC8801_0443 PCC8801_0442 PCC8801_0441 | - - - | TGCACCGATGTGCT | -381 | 7.46E+00 | 0.002084 | Y |
| 12 | PCC8801_0165 | - | GGTACTTCTGTTGT | 27 | 7.45E+00 | 0.002215 | Y |
| 13 | PCC8801_1159 | - | GCTACACCTGTACT | -365 | 7.40E+00 | 0.002484 | Y |
| 14 | PCC8801_1668 PCC8801_1667 PCC8801_1666 | - - - | CGTACAGAAGTAGT | -223 | 7.39413 | 0.002553 | Y |
| 15 | PCC8801_3236 PCC8801_3237 PCC8801_3238 PCC8801_3239 PCC8801_3240 | - - - - - | AGTTCAAATGTTCT | -129 | 7.387369 | 0.002643 | Y |
| 16 | PCC8801_2932 | - | TGTACCGTTGTTAT | 23 | 7.36535 | 0.002813 | Y |
| 17 | PCC8801_2404 | - | AGTACAATTGTATT | -296 | 7.3458 | 0.003009 | N |
| 18 | PCC8801_2539 | - | AGTACCCCTGTATG | -76 | 7.315501 | 0.003406 | Y |
| 19 | PCC8801_3447 PCC8801_3446 PCC8801_3445 PCC8801_3444 | - - - - | CGCACAATTGTACG | -137 | 7.303746 | 0.003595 | Y |
| 20 | PCC8801_2590 | - | GGTACAAATGTATT | -183 | 7.2879 | 0.003934 | N |
| 21 | PCC8801_1779 PCC8801_1778 | - - | AGTACAGTCGTGAT | -97 | 7.278839 | 0.004097 | Y |
| 22 | PCC8801_1518 | - | CGTACTACTGTATA | -646 | 7.263065 | 0.004294 | Y |
| 23 | PCC8801_0945 | - | AAAACTCTTGTACT | -38 | 7.249355 | 0.004659 | Y |
| 24 | PCC8801_1431 | - | TGTCCGAATGTCCT | -46 | 7.193224 | 0.005799 | Y |
| 25 | PCC8801_4299 | - | AGAACAAATGTATC | 5 | 7.186514 | 0.006028 | Y |
| 26 | PCC8801_2993 PCC8801_2994 PCC8801_2995 | - - - | TGAACAATTGTCCA | -190 | 7.181257 | 0.006028 | Y |
| 27 | PCC8801_1777 PCC8801_1776 PCC8801_1775 | - - - | GGTTCCATTGTCCT | -567 | 7.152383 | 0.006802 | Y |
| 28 | PCC8801_1616 | - | AGGACATTTGTTAT | -96 | 7.13868 | 0.007315 | Y |
| 29 | PCC8801_4235 | - | TGTACCATTGGATT | -248 | 7.132036 | 0.007315 | Y |
| 30 | PCC8801_1464 | - | GGAACTTTTGTACC | -74 | 7.120829 | 0.007578 | Y |
| 31 | PCC8801_3235 PCC8801_3234 | - - | AGAACATTTGAACT | -48 | 7.115213 | 0.007841 | Y |
| 32 | PCC8801_1307 | - | TGGACAAAAGTTCT | 20 | 7.107591 | 0.008131 | Y |
| 33 | PCC8801_2997 | - | GGTACAAAAGTCCT | -72 | 7.104379 | 0.008131 | Y |
| 34 | PCC8801_1072 PCC8801_1073 | - - | TGTACAATTGTTGA | -138 | 7.100756 | 0.008131 | Y |
| 35 | PCC8801_0762 PCC8801_0763 PCC8801_0764 PCC8801_0765 PCC8801_0766 PCC8801_0767 PCC8801_0768 | - - - - - - - | ATTACAACTGTATT | -281 | 7.0997 | 0.008421 | Y |
| 36 | PCC8801_1539 | - | GGTACTATGGTAAT | -58 | 7.091811 | 0.008421 | Y |
| 37 | PCC8801_3410 | - | TCTGCTTTTGTACT | 34 | 7.086321 | 0.008763 | Y |
| 38 | PCC8801_3130 | - | AGAACAAACGTGCT | 3 | 7.075004 | 0.009062 | Y |

**Table S4**. Predicted LexA binding sites in *Microcystis aeruginosa* NIES 843 genome at *P* < 0.01

| Rank | Transcription Unit | Name | Putative LexA Binding Site | Position | Score | p-value | Have an orthologue or not(Y/N) |
| --- | --- | --- | --- | --- | --- | --- | --- |
| 1 | MAE_25010 MAE_25000 | - - | AGTACCATTGTTCT | -584 | 8.031016 | 0.000157 | Y |
| 2 | MAE_62330 | - | AGGACAAATGTACT | -107 | 8.017179 | 0.00018 | Y |
| 3 | MAE_57020 MAE_57030 MAE_57040 MAE_57050 | - - - - | AGGACAAATGTACT | -150 | 7.9858 | 0.000228 | Y |
| 4 | MAE_33660 | - | AGGACAAATGTACT | -166 | 7.965228 | 0.000265 | Y |
| 5 | MAE_31210 MAE_31220 | - - | AGGACTAATGTACT | -329 | 7.954587 | 0.000289 | Y |
| 6 | MAE_39910 MAE_39920 | - - | AGGACAAATGTACT | -312 | 7.911563 | 0.000382 | Y |
| 7 | MAE_38440 | - | AGGACGAATGTACT | -265 | 7.897863 | 0.000436 | Y |
| 8 | MAE_23250 MAE_23240 | - - | CGTACATTTGTATT | -114 | 7.856105 | 0.000526 | Y |
| 9 | MAE_16070 | recA | CATACTGCTGTACT | -28 | 7.851116 | 0.000526 | Y |
| 10 | MAE_37940 | - | AGCACCATTGTACT | -42 | 7.850766 | 0.000526 | Y |
| 11 | MAE_09700 | ilvC | GGCACGAATGTACT | 16 | 7.819029 | 0.000605 | Y |
| 12 | MAE_03560 MAE_03570 | - - | AGAACAAGTGTATT | -511 | 7.729516 | 0.000829 | Y |
| 13 | MAE_23210 | - | AGGACGAATGTACT | -268 | 7.70767 | 0.000899 | Y |
| 14 | MAE_14540 MAE_14530 | - - | TGAACATTTGTACT | -39 | 7.610943 | 0.001284 | Y |
| 15 | MAE_18340 | pstB2 | AGTACCGATGTTGT | 20 | 7.592256 | 0.001358 | Y |
| 16 | MAE_15850 | - | AGTACAAATGTTCA | -101 | 7.582842 | 0.001391 | Y |
| 17 | MAE_14550 MAE_14560 | crtE - | AGTACAAATGTTCA | -150 | 7.536755 | 0.001623 | Y |
| 18 | MAE_23880 MAE_23870 | zam - | AGTTCTTTTGTACT | -394 | 7.525763 | 0.001676 | Y |
| 19 | MAE_37950 | - | AGTACAATGGTGCT | -31 | 7.510983 | 0.001723 | Y |
| 20 | MAE_03370 MAE_03380 MAE_03390 | - - - | AGTTCTTTTGTACT | -13 | 7.49593 | 0.001819 | Y |
| 21 | MAE_54470 | sigA | GGTGCAAGTGTACT | -465 | 7.436058 | 0.002236 | Y |
| 22 | MAE_32760 | - | AGTGCTAATGTACC | 7 | 7.431329 | 0.002236 | Y |
| 23 | MAE_19220 MAE_19230 | petC1 petA | GGTACAGATGTTCC | 27 | 7.400313 | 0.002524 | Y |
| 24 | MAE_38490 MAE_38500 MAE_38510 | - - - | GGTACAAATGTATG | 5 | 7.36832 | 0.002919 | Y |
| 25 | MAE_45190 | - | AGTACAAGTGTATT | -467 | 7.359 | 0.003036 | N |
| 26 | MAE_48170 MAE_48180 MAE_48190 | - - - | GGTGCTTTTGTACT | 22 | 7.350593 | 0.003036 | Y |
| 27 | MAE_36470 MAE_36460 | - - | AGTACGGGTGTTCA | -693 | 7.345834 | 0.003181 | Y |
| 28 | MAE_50770 | - | ATTACTTATGTCCT | 5 | 7.313199 | 0.003585 | Y |
| 29 | MAE_54960 MAE_54970 | - - | TGTACTTATGTAGG | -260 | 7.31039 | 0.003585 | Y |
| 30 | MAE_48460 | - | AGGACAAATGTACT | -96 | 7.3055 | 0.003688 | N |
| 31 | MAE_07840 | - | AGGACAAATGTACT | -108 | 7.3055 | 0.003688 | N |
| 31 | MAE_08970 MAE_08980 | - - | AGGACAAATGTACT | -73 | 7.3055 | 0.003688 | N |
| 31 | MAE_27100 | - | AGGACAAATGTACT | -84 | 7.3055 | 0.003688 | N |
| 31 | MAE_39060 | ssb | CATACTATTGTACT | -19 | 7.299533 | 0.003801 | Y |
| 32 | MAE_20310 | - | TCTACTCTTGTACT | 32 | 7.258038 | 0.004418 | Y |
| 33 | MAE_20070 MAE_20080 | - - | CGGACTTGTGTTCT | -322 | 7.23884 | 0.004714 | Y |
| 34 | MAE_56930 | efp | AGTAATATTGTACT | -176 | 7.223731 | 0.004856 | Y |
| 35 | MAE_10860 MAE_10850 | - - | AGGACGAATGTACT | -153 | 7.2235 | 0.004856 | N |
| 36 | MAE_25680 MAE_25670 | dnaB - | TGAACATTTGTATT | -57 | 7.208797 | 0.005098 | Y |
| 37 | MAE_20150 | - | CGGACTTCTGTATT | -164 | 7.18154 | 0.005428 | Y |
| 38 | MAE_27750 MAE_27760 | - - | GGTAAAAATGTACT | -157 | 7.173744 | 0.005569 | Y |
| 39 | MAE_34290 | - | TGTACCCATGTAAT | -53 | 7.1594 | 0.005863 | N |
| 40 | MAE_29680 | - | TGTACCCATGTAAT | -53 | 7.1594 | 0.005863 | N |
| 40 | MAE_34130 | - | TGTACCCATGTAAT | -55 | 7.1594 | 0.005863 | N |
| 40 | MAE_59610 MAE_59620 | - - | TGTACAGGTGTGCA | -284 | 7.156886 | 0.005863 | Y |
| 41 | MAE_52660 | phoU | TGGACAACTGTACA | -66 | 7.156672 | 0.005863 | Y |
| 42 | MAE_31110 | - | AGTACAAGCGTACG | -382 | 7.155835 | 0.005863 | Y |
| 43 | MAE_45590 | leuS | GGTAAAGTTGTACT | -435 | 7.134442 | 0.006142 | Y |
| 44 | MAE_62810 MAE_62820 MAE_62830 MAE_62840 | - - - - | GGTACTTATGAAGT | 5 | 7.105518 | 0.006646 | Y |
| 45 | MAE_39700 | - | CGCACTGCTGTACA | 35 | 7.105397 | 0.006646 | Y |
| 46 | MAE_14230 | - | AATACTTTTGTTCT | -207 | 7.101824 | 0.006646 | Y |
| 47 | MAE_27160 MAE_27150 | - - | TGTACCGATGTAAG | -346 | 7.06709 | 0.00738 | Y |
| 48 | MAE_30820 MAE_30830 MAE_30840 MAE_30850 MAE_30860 MAE_30870 | - - - cas4 cas1 cas2 | CGTTCGATTGTCCT | -25 | 7.062981 | 0.00738 | Y |
| 49 | MAE_25790 | - | AGTTCAGGTGTATT | -482 | 7.059064 | 0.007591 | Y |
| 50 | MAE_26130 | - | AGTACAAATGTTTG | 7 | 7.05727 | 0.007591 | Y |
| 51 | MAE_58700 | - | TGAGCATTTGTACT | -165 | 7.056071 | 0.007591 | Y |
| 52 | MAE_48900 | glyS | GGTACAGAAGAACT | 35 | 7.04323 | 0.007846 | Y |
| 53 | MAE_43020 MAE_43030 MAE_43040 MAE_43050 | - - - - | GGTTCTCGTGTATT | -536 | 7.040026 | 0.007846 | Y |
| 54 | MAE_13690 | - | AGCACGATTGTCAT | -134 | 7.03861 | 0.008099 | Y |
| 55 | MAE_03870 MAE_03860 MAE_03850 | - - - | AGCACTGTTGTTGT | -315 | 7.038041 | 0.008099 | Y |
| 56 | MAE_51940 | - | AGTACTTTTGGATT | -195 | 7.035672 | 0.008099 | Y |
| 57 | MAE_49970 MAE_49980 | - - | GCGACAATTGTACT | -152 | 7.033693 | 0.008099 | Y |
| 58 | MAE_56340 | - | AGTGCTAATGTACC | 5 | 7.032225 | 0.008099 | Y |
| 59 | MAE_46340 | petG | AGTACAGACGTGAT | 3 | 7.023686 | 0.008344 | Y |
| 60 | MAE_44810 MAE_44820 MAE_44830 | ycf4 ppiB - | AGTACCCTTGATCT | -131 | 7.02129 | 0.008344 | Y |
| 61 | MAE_31820 | - | AGTACAAGTGAATT | -2 | 7.020509 | 0.008344 | Y |
| 62 | MAE_13030 | - | GGGACTGATGTACG | -197 | 7.017173 | 0.008539 | Y |
| 63 | MAE_23890 | - | AGTACAAAAGAACT | -15 | 7.014193 | 0.008539 | Y |
| 64 | MAE_37250 | - | ATTACGAATGTAGT | -321 | 7.011981 | 0.008539 | Y |
| 65 | MAE_53810 | - | ACTACACTTGTAAC | -37 | 7.008554 | 0.00881 | Y |
| 66 | MAE_45080 MAE_45070 | - - | GGAACCGCCGTACT | 23 | 7.002743 | 0.00881 | Y |
| 67 | MAE_41820 | ureF | AGAACTTTTGTGTT | 26 | 6.998566 | 0.009025 | Y |
| 68 | MAE_53930 | dnaX | CATACACTTGTTCT | -68 | 6.995018 | 0.009025 | Y |
| 69 | MAE_52260 | ndhH | AGTCCTTGTGTATT | -57 | 6.988069 | 0.009275 | Y |
| 70 | MAE_30180 | infC | AGAACGCCTGTGAT | 3 | 6.98775 | 0.009275 | Y |
| 71 | MAE_05920 | - | AGAACAAAAGTTCT | -284 | 6.987214 | 0.009275 | Y |
| 72 | MAE_08000 MAE_07990 | - - | TGTGCTAATGTCCT | -128 | 6.984737 | 0.009275 | Y |
| 73 | MAE_41780 | - | AGAACAAATGTATG | -190 | 6.97977 | 0.00952 | Y |

**Table S5**. Predicted LexA binding sites in *Nostoc punctiforme* PCC73102 genome at *P* < 0.01

| **Rank** | **Transcription Unit** | **Name** | **Putative LexA Binding Site** | **Position** | **Score** | **p-value** | **Have an orthologue or not(Y/N)** |
| --- | --- | --- | --- | --- | --- | --- | --- |
| 1 | Npun_R5568 | - | CGTACATTTGTACT | -34 | 8.743842 | 0 | Y |
| 2 | Npun_F1608 | - | AGTACCCTTGTTCT | -305 | 8.618254 | 0 | Y |
| 3 | Npun_F4214 Npun_F4215 Npun_F4216 | - - - | AGTACTTTTGTACT | -245 | 8.544845 | 0 | Y |
| 4 | Npun_R1841 | - | AGTACAGGTGTACT | -299 | 8.541434 | 0 | Y |
| 5 | Npun_F1842 | - | AGTACACCTGTACT | -16 | 8.529292 | 0 | Y |
| 6 | Npun_F1319 | - | TGTACTACTGTACT | -41 | 8.388509 | 4.17E-06 | Y |
| 7 | Npun_F4481 Npun_F4482 | - - | AGTACTAATGTTCT | -33 | 8.383097 | 4.17E-06 | Y |
| 8 | Npun_R5583 Npun_R5582 | - - | TGTACTCCTGTACT | -400 | 8.289947 | 2.08E-05 | Y |
| 9 | Npun_F6100 Npun_F6101 Npun_F6102 | - - - | AGTACGATTGTTCT | -71 | 8.28095 | 2.08E-05 | Y |
| 10 | Npun_R6309 | - | AGTACAAGTGTCCT | -160 | 8.243507 | 2.92E-05 | Y |
| 11 | Npun_R4122 | - | AGTACATTTGTTCT | -116 | 8.154036 | 7.57E-05 | Y |
| 12 | Npun_F4120 | - | AGTACAAGTGTTCT | -426 | 8.091997 | 0.000166 | Y |
| 13 | Npun_R3959 | - | TGTACAATTGTATT | -359 | 8.063759 | 0.000231 | Y |
| 14 | Npun_F4123 | - | AGAACAAATGTACT | -225 | 8.012311 | 0.000333 | Y |
| 15 | Npun_R5088 | - | AGTACAAATATACT | -50 | 7.981514 | 0.000388 | Y |
| 16 | Npun_F5010 Npun_F5011 | - - | AGTACAGGTGTACC | -48 | 7.967569 | 0.000427 | Y |
| 17 | Npun_F4803 | - | AGTACAGATGTACA | -73 | 7.954866 | 0.00044 | Y |
| 18 | Npun_F0455 | - | AGTACTGATGTAGT | -341 | 7.914327 | 0.000516 | Y |
| 19 | Npun_F1577 | - | AGTGCATCTGTACT | -39 | 7.908289 | 0.000534 | Y |
| 20 | Npun_R2779 Npun_R2778 Npun_R2777 | - - - | AATACAAGTGTAGT | -23 | 7.906413 | 0.000534 | Y |
| 21 | Npun_R1533 Npun_R1532 | - - | AGAACTTTTGTATT | -22 | 7.847171 | 0.000706 | Y |
| 22 | Npun_R6240 | - | ATTACACATGTACA | 32 | 7.803679 | 0.000829 | Y |
| 23 | Npun_F4523 | - | CGTACATCTGTACG | -81 | 7.798663 | 0.000871 | Y |
| 24 | Npun_R2389 | - | AAAACTGATGTACT | 38 | 7.796025 | 0.000871 | Y |
| 25 | Npun_R1071 | - | TGTACAGCTGTACA | -287 | 7.759663 | 0.001022 | Y |
| 26 | Npun_F0446 | - | TGTACTATTGTAGT | -343 | 7.749229 | 0.001057 | Y |
| 27 | Npun_F4948 | - | AGTACAAATGTTCG | -345 | 7.742488 | 0.001057 | Y |
| 28 | Npun_R1069 | - | AGCACAGATGTGCT | -541 | 7.742046 | 0.001057 | Y |
| 29 | Npun_F5332 | - | TGCACACGTGTGCA | -26 | 7.733714 | 0.001094 | Y |
| 30 | Npun_R4925 | - | GGCACGGGTGTACT | -495 | 7.708105 | 0.001195 | Y |
| 31 | Npun_F3763 | - | ATTACTTTTGTCCT | -468 | 7.704968 | 0.001195 | Y |
| 32 | Npun_F6211 | - | TGTACACCTGTACC | -669 | 7.690502 | 0.001236 | Y |
| 33 | Npun_F2780 | - | ACTACACTTGTATT | -36 | 7.686214 | 0.001282 | Y |
| 34 | Npun_F4142 | - | GGTACTGTTGTGGC | 32 | 7.669654 | 0.001362 | Y |
| 35 | Npun_F6572 | - | CGTACAGATGTACA | 5 | 7.628496 | 0.001559 | Y |
| 36 | Npun_R1134 Npun_R1133 | - - | AGTACTAATGCACT | -309 | 7.620022 | 0.001559 | Y |
| 37 | Npun_F4149 | - | AGTACGAGTGTTCA | -364 | 7.602481 | 0.001677 | Y |
| 38 | Npun_F5722 Npun_F5723 | - - | CGTACAACTTTACT | 20 | 7.597771 | 0.00174 | Y |
| 39 | Npun_F0700 | - | AATACATTTGTATT | -157 | 7.597204 | 0.00174 | Y |
| 40 | Npun_F4808 | - | AGTACAAATGTCCC | -23 | 7.596126 | 0.00174 | Y |
| 41 | Npun_R3821 | - | TGTACTTATGTTGT | -141 | 7.581404 | 0.001813 | Y |
| 42 | Npun_R2955 | - | AGTACAAATATACT | -87 | 7.580955 | 0.001813 | Y |
| 43 | Npun_F1013 | - | TGTACTGATGCACT | -83 | 7.553908 | 0.002027 | Y |
| 44 | Npun_F2029 | - | AGTACGGGTGAACT | -16 | 7.50415 | 0.002557 | Y |
| 45 | Npun_R5494 | - | TGTACTTATGTCAT | 7 | 7.499447 | 0.002696 | Y |
| 46 | Npun_F0771 | - | AGCACATCTGTATT | -116 | 7.489823 | 0.002837 | Y |
| 47 | Npun_R1550 | - | CGTACCCCTGTGCG | -209 | 7.48799 | 0.002837 | Y |
| 48 | Npun_F1207 | - | ATTACATTTGTAGT | -471 | 7.485036 | 0.002837 | Y |
| 49 | Npun_R0420 | - | AGTACAAAAGTAAT | -370 | 7.436469 | 0.003584 | Y |
| 50 | Npun_F0946 | - | TGTACCAGTGAACT | -82 | 7.399999 | 0.004373 | Y |
| 51 | Npun_R2304 Npun_R2303 | - - | TTTACCATTGTACT | -31 | 7.399029 | 0.004373 | Y |
| 52 | Npun_F2914 | - | AGTATATCTGTTCT | -62 | 7.397235 | 0.004373 | Y |
| 53 | Npun_R2017 | - | AGTACTTGGGTATT | -219 | 7.396943 | 0.004373 | Y |
| 54 | Npun_R0273 | - | TGTACTGATGTTCA | -57 | 7.390428 | 0.004373 | Y |
| 55 | Npun_F2668 | - | AGTACTTTTGTATG | -276 | 7.386091 | 0.004614 | Y |
| 56 | Npun_F1891 | - | AGTACCTCTGTTGT | -60 | 7.379887 | 0.004819 | Y |
| 57 | Npun_F0556 | - | AGAACAAATGTAAT | -95 | 7.379615 | 0.004819 | Y |
| 58 | Npun_F0692 | - | GGTACTGTTGCACT | -337 | 7.370662 | 0.004819 | Y |
| 59 | Npun_F2956 | - | AGTATATTTGTACT | -520 | 7.356459 | 0.005262 | Y |
| 60 | Npun_F0905 | - | GGTACTGTTGTTGT | -138 | 7.354175 | 0.005262 | Y |
| 61 | Npun_F5018 | - | TGTACTTGTGTTTT | -404 | 7.34112 | 0.00548 | Y |
| 62 | Npun_R2751 | - | AGAACGAATGTACA | 7 | 7.331653 | 0.005683 | Y |
| 63 | Npun_F2708 | - | AGTACAAATGCTCT | -70 | 7.322467 | 0.00593 | Y |
| 64 | Npun_R0245 | - | CGAACTATTGTTCT | -106 | 7.310975 | 0.006174 | Y |
| 65 | Npun_F5451 | - | AGTGCCTCTGTACT | -130 | 7.309507 | 0.006415 | Y |
| 66 | Npun_F6197 | - | GATACTTATGTACT | -201 | 7.307752 | 0.006415 | Y |
| 67 | Npun_F4453 Npun_F4454 | - - | TGTACTAGTGTGCA | -167 | 7.306242 | 0.006415 | Y |
| 68 | Npun_F5440 | - | ATTACTCTTGTAAT | -599 | 7.290987 | 0.006658 | Y |
| 69 | Npun_F1636 | - | GCTACTTTTGTACT | -770 | 7.27825 | 0.007182 | Y |
| 70 | Npun_F2820 | - | TATACTTTTGTAGT | -240 | 7.276205 | 0.007182 | Y |
| 71 | Npun_R6495 | - | AGCACAGTCGTACT | -137 | 7.264728 | 0.007456 | Y |
| 72 | Npun_R1496 Npun_R1495 | - - | AGTACTATTGCTCT | -60 | 7.259519 | 0.007724 | Y |
| 73 | Npun_F6435 | - | AGTACTTGTGTATT | -179 | 7.2572 | 0.007724 | N |
| 74 | Npun_R0445 | - | AGTACATTGGTAAT | 2 | 7.255444 | 0.007724 | Y |
| 75 | Npun_F1056 | - | TGAACGCTTGTAGT | -178 | 7.253743 | 0.007724 | Y |
| 76 | Npun_R1984 Npun_R1983 | - - | AGCACGGGTGTTAT | -144 | 7.252679 | 0.007724 | Y |
| 77 | Npun_F1775 Npun_F1776 | - - | AGTACTTAAGTTAT | -117 | 7.251207 | 0.007724 | Y |
| 78 | Npun_R3824 | - | TTTACCCTTGTACT | -659 | 7.248562 | 0.007987 | Y |
| 79 | Npun_F1918 Npun_F1919 Npun_F1920 | - - - | CGGACAAATGTATT | -240 | 7.244843 | 0.007987 | Y |
| 80 | Npun_F3086 | - | GGTAATTATGTACA | 5 | 7.242055 | 0.007987 | Y |
| 81 | Npun_F2360 | - | GGTACTATTGTAGC | -742 | 7.238126 | 0.008287 | Y |
| 82 | Npun_F5089 | - | AGTATATTTGTACT | -219 | 7.233419 | 0.008287 | Y |
| 83 | Npun_R5170 | - | AGTACGGACGTTAT | -208 | 7.230707 | 0.008287 | Y |
| 84 | Npun_R2361 | - | CGTAAACTTGTACT | -620 | 7.224777 | 0.008588 | Y |
| 85 | Npun_F0354 | - | GGCACTTTTGTAAT | -326 | 7.220032 | 0.008588 | Y |
| 86 | Npun_F1217 | - | AGTTCTTGTGTTCT | -71 | 7.215025 | 0.008884 | Y |
| 87 | Npun_R4905 | - | TAAACCCGTGTACT | 7 | 7.203066 | 0.009186 | Y |
| 88 | Npun_R3495 | - | AGTGCCAGTGTGCT | -386 | 7.201941 | 0.009186 | Y |
| 89 | Npun_F0699 | - | GGTACTCATGTATC | -214 | 7.194685 | 0.009466 | Y |

**Table S6**. Predicted LexA binding sites in *Nostoc sp* PCC7120 genome at *P* < 0.01

| **Rank** | **Transcription Unit** | **Name** | **Putative LexA Binding Site** | **Position** | **Score** | **p-value** | **Have an orthologue or not(Y/N)** |
| --- | --- | --- | --- | --- | --- | --- | --- |
| 1 | all2103 all2102 | - ama | AGTACTTATGTACT | -173 | 9.7674 | 0 | Y |
| 2 | alr1056 | - | AGTACAATTGTACT | -50 | 9.496702 | 0 | Y |
| 3 | alr2735 | - | AGTACAATTGTACT | -62 | 9.285223 | 0 | Y |
| 4 | alr0647 | - | CGTACAAATGTACT | -438 | 8.90059 | 0 | Y |
| 5 | alr0088 | - | AGTACTTATGTACT | -16 | 8.522966 | 9.10E-05 | Y |
| 6 | alr3140 | - | TGTACCAGTGTCAT | -449 | 8.46E+00 | 0.000143 | Y |
| 7 | alr2104 | - | AGTACATAAGTACT | -47 | 8.45E+00 | 0.000164 | Y |
| 8 | alr0203 | - | AGTACAATTGCACT | -110 | 8.44E+00 | 0.000164 | Y |
| 9 | asl1412 | - | AGTACAATAGTACT | -29 | 8.43E+00 | 0.000168 | Y |
| 10 | alr4908 | - | AGTACTAATGTTCT | -35 | 8.39E+00 | 0.000216 | Y |
| 11 | asl0401 | - | AGTACTTTTGTACT | -57 | 8.38E+00 | 0.000216 | Y |
| 12 | alr0402 | - | AGTACAAAAGTACT | -300 | 8.335393 | 0.000263 | Y |
| 13 | alr4714 | - | GGTACTGCTGTGGT | 32 | 8.207958 | 0.000314 | Y |
| 14 | all3503 | - | CGAACTGATGTACT | -24 | 8.188043 | 0.000321 | Y |
| 15 | all4790 | - | CGTACATTTGTACC | -31 | 8.164916 | 0.000329 | Y |
| 16 | all3275 all3274 | - - | TGTACTGGTGTAGC | -73 | 8.162875 | 0.000329 | Y |
| 17 | all1774 asl1773 | xseA xseB | TGTACTAATGTATT | -10 | 8.154232 | 0.000336 | Y |
| 18 | alr3716 | uvrA | AGTACTATTGTTCT | -72 | 8.148045 | 0.000341 | Y |
| 19 | alr2019 | - | GGTACTTTGGTACT | -118 | 8.122379 | 0.000346 | Y |
| 20 | all4518 | - | AGTACAGGTGTAGT | -33 | 8.046216 | 0.000391 | Y |
| 21 | all1440 | nifK | AGTACTTTTGTTCT | -579 | 8.030693 | 0.000398 | Y |
| 22 | alr1370 | - | TGTACTTATGTTCT | 5 | 8.008135 | 0.000416 | Y |
| 23 | all2910 | - | TGTACTTCTGTTCT | -432 | 7.983195 | 0.000429 | Y |
| 24 | alr3285 | - | GGTACCTCTGTAAC | -208 | 7.963346 | 0.00045 | Y |
| 25 | alr3504 | - | GGTACTTAAGTACT | -679 | 7.944066 | 0.000477 | Y |
| 26 | all4830 all4829 | - - | AGTACGTCTGTATT | -196 | 7.922416 | 0.000507 | Y |
| 27 | alr4629 | - | AATACAACTGTAAT | -228 | 7.9204 | 0.000507 | Y |
| 28 | asr0855 | - | AATACAAGTGTAGT | -25 | 7.904663 | 0.000541 | Y |
| 29 | alr4905 | - | AGTTCTCATGTACT | -100 | 7.883651 | 0.000594 | Y |
| 30 | all4969 | - | AGTACGATTGTAGT | -173 | 7.881787 | 0.000594 | Y |
| 31 | all1252 | - | AGTACCTGAGTCCT | -205 | 7.879305 | 0.000611 | Y |
| 32 | alr3920 | - | GGTACTTCTGTGAT | 3 | 7.867167 | 0.000639 | Y |
| 33 | all2245 all2244 | - - | CGTACTTTTGTCAT | -153 | 7.865655 | 0.000639 | Y |
| 34 | all4375 | - | GGTACACCTGTAGT | -517 | 7.8597 | 0.000669 | Y |
| 35 | all0646 | - | AGTACATTTGTACG | -87 | 7.848856 | 0.000706 | Y |
| 36 | all3048 | - | AGTACTAATGTTTT | -111 | 7.840758 | 0.000706 | Y |
| 37 | all2845 asl2844 | - - | TGTACAAATGTTCA | 23 | 7.831896 | 0.000738 | Y |
| 38 | alr3988 | - | AGTGCATCTGTACT | -40 | 7.821639 | 0.000777 | Y |
| 39 | alr3466 | - | GGTACCATTGTATT | -545 | 7.811461 | 0.000819 | Y |
| 40 | alr2122 alr2123 | - - | AGTACTGCTGTGAT | 24 | 7.786257 | 0.000953 | Y |
| 41 | alr0892 alr0893 | - - | ATTACTCATGTACA | 30 | 7.785548 | 0.000953 | Y |
| 42 | asr0636 | - | AGTACGGTTGTAAA | -53 | 7.779721 | 0.001005 | Y |
| 43 | all2567 | - | GGTACAAATGTATA | -136 | 7.773127 | 0.001005 | Y |
| 44 | alr1041 | - | AGTACTTAAGTACT | -163 | 7.771601 | 0.001005 | Y |
| 45 | all3465 | - | GGTACCCATGTACC | -348 | 7.745206 | 0.001163 | Y |
| 46 | asr4321 | - | TGAACTTTTGTACT | -54 | 7.742828 | 0.001163 | Y |
| 47 | alr4582 | - | AGTACATCTGTGTT | -311 | 7.741686 | 0.001163 | Y |
| 48 | all1123 | - | GTTACTTATGTCCT | -73 | 7.727514 | 0.001272 | Y |
| 49 | alr4649 alr4650 | - - | AGTACCTTTGTGGT | 36 | 7.684715 | 0.001553 | Y |
| 50 | all0854 | - | ACTACACTTGTATT | -32 | 7.68353 | 0.001553 | Y |
| 51 | asr4942 alr4943 alr4944 | - - - | CGTACCATAGTACT | -41 | 7.635583 | 0.002027 | Y |
| 52 | alr3411 | - | AGTACTAGTGTTAT | -112 | 7.632658 | 0.002027 | Y |
| 53 | all4052 | - | AGCACGAATGTCTT | -237 | 7.632057 | 0.002027 | Y |
| 54 | alr2708 | - | AGTACGAATGTTGT | -620 | 7.595144 | 0.002523 | Y |
| 55 | asr3935 alr3936 alr3937 | - hisC - | GGTGCAACTGTACT | -32 | 7.584825 | 0.002642 | Y |
| 56 | alr1232 | - | CGTTCATTTGTACT | -531 | 7.559013 | 0.003027 | Y |
| 57 | asl4186 | rpl31 | TGTACTGGGGTACT | -476 | 7.548874 | 0.003172 | Y |
| 58 | all2056 | - | TGTACAGTTGTCTT | -70 | 7.548274 | 0.003172 | Y |
| 59 | asl1446 all1445 | - - | CGCACCTACGTAAT | -148 | 7.54725 | 0.003172 | Y |
| 60 | all2004 | - | TGTGCATCTGTCCT | -56 | 7.508078 | 0.003702 | Y |
| 61 | asr2016 | - | TTTACGATTGTGCT | -58 | 7.507369 | 0.003702 | Y |
| 62 | all1636 | - | AGTACCATTGTCTT | -659 | 7.493514 | 0.003843 | Y |
| 63 | all3793 | - | ACTACGTCTGTGAT | -5 | 7.4916 | 0.003843 | Y |
| 64 | all2909 | - | GCTACATCTGTACT | -449 | 7.488839 | 0.003999 | Y |
| 65 | all2989 | - | ATTACAACTGTTCT | -6 | 7.457981 | 0.004464 | Y |
| 66 | alr3238 | - | CGTACTTATGATAT | -354 | 7.443825 | 0.004633 | Y |
| 67 | all2316 | - | CGTACCCCTGTTGT | -417 | 7.442745 | 0.004633 | Y |
| 68 | all4801 | - | AGTACTATTGCAGT | -44 | 7.429307 | 0.004916 | Y |
| 69 | alr1052 | - | CGTACTCAAGTAGT | -46 | 7.42847 | 0.004916 | Y |
| 70 | all5089 all5088 | - - | AATACATCTGTACT | -106 | 7.425821 | 0.004916 | Y |
| 71 | all3194 all3193 asl3192 | - - - | CGTACAAATGTAAG | -210 | 7.424036 | 0.004916 | Y |
| 72 | alr5147 | - | AATACCGATGTACT | -31 | 7.421396 | 0.004916 | Y |
| 73 | alr4009 alr4010 alr4011 | - - - | CGTATATTTGTAAT | -106 | 7.418675 | 0.005098 | Y |
| 74 | asl2332 | - | CGTGCTGCTGTTCC | 20 | 7.416 | 0.005098 | Y |
| 75 | alr0377 | - | GGTTCTTATGTATT | -197 | 7.415363 | 0.005098 | Y |
| 76 | all1132 | uvrB | AGTACAGATGTATG | 3 | 7.409794 | 0.005242 | Y |
| 77 | all3272 | recA | AGTATATCTGTTCT | -52 | 7.402284 | 0.005242 | Y |
| 78 | alr3495 | - | TATACTTTTGTCCT | -81 | 7.401107 | 0.005242 | Y |
| 79 | asl0097 | - | TATACTTCTGTAGT | -677 | 7.398142 | 0.005399 | Y |
| 80 | alr4239 alr4240 | - - | GGTACTTTTGTTCG | -229 | 7.39717 | 0.005399 | Y |
| 81 | asl3860 | - | AGTACCAAAGTTCT | -32 | 7.395998 | 0.005399 | Y |
| 82 | all1814 | - | GCGACAGTTGTACT | 29 | 7.395229 | 0.005399 | Y |
| 83 | all3859 | - | AGTACCAAAGTTCT | -412 | 7.390933 | 0.005399 | Y |
| 84 | alr3915 | - | TGTGCATCTGTATT | 26 | 7.385859 | 0.005582 | Y |
| 85 | alr3170 | - | CGTACAAGCGTACA | -86 | 7.369158 | 0.005934 | Y |
| 86 | alr2990 alr2991 | dnaK dnaJ | AGAACAGTTGTAAT | -401 | 7.366308 | 0.005934 | Y |
| 87 | alr4412 | - | GGTACTAATGTGAT | -106 | 7.36129 | 0.005934 | Y |
| 88 | all0050 | - | GGATCGAGTGTTCT | -277 | 7.3518 | 0.006104 | Y |
| 89 | asr0081 | - | TCTACTGCTGTAAT | -47 | 7.344313 | 0.006297 | Y |
| 90 | all2436 | - | TGTACTGAGGTTCT | -119 | 7.341397 | 0.006297 | Y |
| 91 | all0275 | - | AGCACCAATGTCCA | -473 | 7.341296 | 0.006297 | Y |
| 92 | all5196 all5195 | - - | AGTACAAATGAACA | 7 | 7.341133 | 0.006297 | Y |
| 93 | all3983 | - | GGTACATCTGCGCT | -1 | 7.330906 | 0.0065 | Y |
| 94 | alr1324 | - | TGTACAGTTGTCAG | -112 | 7.323761 | 0.006704 | Y |
| 95 | all5137 all5136 | - moeA | CGGACTTTTGTACA | 23 | 7.322701 | 0.006704 | Y |
| 96 | all3447 | - | TGGACAGTTGTATT | -224 | 7.320954 | 0.006704 | Y |
| 97 | asr3796 | - | CTTACTCCTGTACT | -83 | 7.318248 | 0.006906 | Y |
| 98 | alr3693 | - | GGTACGTAAGTCCT | -259 | 7.31072 | 0.006906 | Y |
| 99 | all4838 | - | ATTACAAATGTATA | -91 | 7.305525 | 0.007115 | Y |
| 100 | alr0451 alr0452 | - - | CATACAACTGTACT | -145 | 7.301443 | 0.007115 | Y |
| 101 | all0997 | - | TGAACGGATGTCAT | -69 | 7.298214 | 0.007333 | Y |
| 102 | alr2940 | - | AGTACTAAAGTGCT | -414 | 7.29279 | 0.007333 | Y |
| 103 | all4799 | - | TGTGCATTTGTGCT | -156 | 7.289533 | 0.007593 | Y |
| 104 | alr1791 | - | ATTACTTCTGTCCT | -78 | 7.289417 | 0.007593 | Y |
| 105 | asr3368 | - | ATTACTCTTGTAAT | -234 | 7.288712 | 0.007593 | Y |
| 106 | alr3706 | - | AGTACGTAGGGACT | -181 | 7.286328 | 0.007593 | Y |
| 107 | all3604 | - | GGTACGAGGGTTTT | -240 | 7.283631 | 0.007593 | Y |
| 108 | alr1081 | - | AGTACAACTGAGGT | -5 | 7.282327 | 0.007593 | Y |
| 109 | alr3376 | - | GGTGCATATGTATT | -125 | 7.28201 | 0.007593 | Y |
| 110 | all2919 all2918 | - - | TTTACTATTGTACG | -81 | 7.277607 | 0.007842 | Y |
| 111 | all0796 | - | AGTACTTTTTTACT | -89 | 7.27235 | 0.007842 | Y |
| 112 | all2568 | - | GGTACAGATGTATA | -326 | 7.260947 | 0.008093 | Y |
| 113 | all0422 all0421 all0420 | - - - | CCTACAACTGTATT | -190 | 7.252357 | 0.008376 | Y |
| 114 | all1075 | - | AGAACGGGTGTACG | -37 | 7.250554 | 0.008376 | Y |
| 115 | alr3199 | - | AGTAGAAGTGTATT | -138 | 7.2493 | 0.00866 | Y |
| 116 | all0284 all0283 all0282 asl0281 | - - - - | AGTACAAATATATT | -51 | 7.2421 | 0.00866 | Y |
| 117 | all0830 | - | CGTCCAAATGTTCT | -563 | 7.241974 | 0.00866 | Y |
| 118 | alr3370 | - | GATACTTATGTCCT | -39 | 7.233297 | 0.008982 | Y |
| 119 | asl3196 | - | AGTGCATCTGTATT | -60 | 7.232896 | 0.008982 | Y |
| 120 | all5023 | - | TGTACAATTGTCGG | -42 | 7.230652 | 0.008982 | Y |
| 121 | alr1538 alr1539 | - - | TGCACCACAGTTCT | 13 | 7.229213 | 0.00932 | Y |
| 122 | alr2882 | exoD | GGTGCATTTGTATT | -44 | 7.22603 | 0.00932 | Y |

**Table S7**. Predicted LexA binding sites in *Prochlorococcus marinus* AS9601 genome at *P* < 0.01

| **Rank** | **Transcription Unit** | **Name** | **Putative LexA Binding Site** | **Position** | **Score** | **p-value** | **Have an orthologue or not(Y/N)** |
| --- | --- | --- | --- | --- | --- | --- | --- |
| 1 | A9601_17691 | recA | AGTACAGATGTACT | -86 | 9.439124 | 0 | Y |
| 2 | A9601_00291 | - | AGTACATATGTATT | -7 | 9.358525 | 0 | Y |
| 3 | A9601_03681 | - | AGTACAGATGTATT | -10 | 9.290348 | 0 | Y |
| 4 | A9601_07391 A9601_07401 A9601_07411 | - - - | AGTACATATGTATT | -10 | 9.178958 | 0 | Y |
| 5 | A9601_07471 | - | AGTACAACTGTATT | -8 | 9.01E+00 | 0 | Y |
| 6 | A9601_07141 | - | CGTACTTTTGTCCT | -323 | 8.845253 | 0 | Y |
| 7 | A9601_16301 | - | AGTACACTTGTACT | -561 | 8.82039 | 0 | Y |
| 8 | A9601_00281 A9601_00271 | - pdxA | AATACATATGTACT | -13 | 8.468268 | 3.09E-06 | Y |
| 9 | A9601_04791 A9601_04801 | - - | AGTACGGATGTTAA | -26 | 8.21458 | 2.47E-05 | Y |
| 10 | A9601_12991 A9601_12981 A9601_12971 | - - - | GATACGTTTGTACT | -105 | 8.185809 | 3.40E-05 | Y |
| 11 | A9601_12791 | - | AGGACCTCTGTTCT | -66 | 8.014561 | 0.000244 | Y |
| 12 | A9601_07461 A9601_07451 A9601_07441 A9601_07431 A9601_07421 | - - - - - | AATACAGTTGTACT | -171 | 7.972565 | 0.000306 | Y |
| 13 | A9601_13001 | - | AGTACAAACGTATC | -32 | 7.908768 | 0.000408 | Y |
| 14 | A9601_10761 A9601_10771 | - - | GGCACAATTGCCCT | -581 | 7.892912 | 0.000424 | Y |
| 15 | A9601_12231 A9601_12221 A9601_12211 A9601_12201 | - gid crtH - | AGAACAATCGTAGT | 20 | 7.866956 | 0.000464 | Y |
| 16 | A9601_00641 | - | CGAACCGCTGTCCG | -96 | 7.806428 | 0.000569 | Y |
| 17 | A9601_15841 | - | TGTACAGCTGTCAA | -43 | 7.784821 | 0.000622 | Y |
| 18 | A9601_18251 A9601_18241 A9601_18231 A9601_18221 | ruvB - - - | AGAACAATTGCAAT | -12 | 7.702344 | 0.000788 | Y |
| 19 | A9601_17771 | - | GTTACTAATGTACT | -223 | 7.641076 | 0.000993 | Y |
| 20 | A9601_17651 A9601_17641 A9601_17631 A9601_17621 A9601_17611 A9601_17601 A9601_17591 A9601_17581 A9601_17571 A9601_17561 A9601_17551 A9601_17541 | rplC rplD rplW rplB rpsS rplV rpsC rplP rpmC rpsQ rplN rplX | TGTACAATTGTAAC | -217 | 7.634436 | 0.001042 | Y |
| 21 | A9601_03591 | - | AGTACATATATACT | -18 | 7.634028 | 0.001042 | Y |
| 22 | A9601_10831 | - | TTTACTCCTGTAAT | -598 | 7.581165 | 0.00137 | Y |
| 23 | A9601_02431 | - | GGAACAACTGCACC | -428 | 7.512343 | 0.002158 | Y |
| 24 | A9601_04271 A9601_04281 A9601_04291 A9601_04301 A9601_04311 A9601_04321 | vsr - - - - - | GGTACGTGGGTATG | -83 | 7.508934 | 0.002322 | Y |
| 25 | A9601_12821 | - | TGGACGCATGAAAT | -39 | 7.471174 | 0.002786 | Y |
| 26 | A9601_10941 A9601_10931 | - purT | AGAACTGTAGTATT | -163 | 7.448533 | 0.003219 | Y |
| 27 | A9601_18761 | - | AGGACTAGCGTCCC | -115 | 7.438311 | 0.003426 | Y |
| 28 | A9601_02201 A9601_02191 A9601_02181 A9601_02171 A9601_02161 | rplJ rplL rnhA pyrD - | AGGACCACTGTTGT | -710 | 7.432715 | 0.003426 | Y |
| 29 | A9601_15761 A9601_15771 A9601_15781 | - - - | GGTACATTTGAGGT | 39 | 7.432602 | 0.003426 | Y |
| 30 | A9601_11231 | - | CGTACATATGTTAG | 5 | 7.407241 | 0.003949 | Y |
| 31 | A9601_04241 A9601_04231 | - - | AGTATATTTGTATT | -49 | 7.401275 | 0.003949 | Y |
| 32 | A9601_01701 | metF | AGTACACTTGTTAA | -21 | 7.361187 | 0.004712 | Y |
| 33 | A9601_03811 A9601_03821 | - - | AGAACTGTTGTGCA | 3 | 7.354899 | 0.004966 | Y |
| 34 | A9601_09241 A9601_09231 A9601_09221 | umuD umuC - | AGTACATATATACT | -12 | 7.34911 | 0.005161 | Y |
| 35 | A9601_11281 A9601_11291 A9601_11301 | - - - | AATACTTCAGTTCT | 24 | 7.33318 | 0.005399 | Y |
| 36 | A9601_15101 | hemN | AGTGCTTATGTGCA | 32 | 7.310777 | 0.005878 | Y |
| 37 | A9601_18601 | mesJ | GGCACTACTGAACT | -303 | 7.305028 | 0.006132 | Y |
| 38 | A9601_14861 A9601_14851 | - ffh | ATTACTCCTGTGCT | -338 | 7.30339 | 0.006132 | Y |
| 39 | A9601_11511 | - | TGGACAACAGTTCT | -499 | 7.300777 | 0.006132 | Y |
| 40 | A9601_15491 | spoIID | GGGACACCTGAGCT | -112 | 7.286907 | 0.006673 | Y |
| 41 | A9601_03801 | - | TGCACAACAGTTCT | -141 | 7.281204 | 0.006673 | Y |
| 42 | A9601_02911 | - | GGTACTCCTGAAAT | -233 | 7.271958 | 0.006908 | Y |
| 43 | A9601_18151 | rimM | AGAACAGCTGCCCT | -135 | 7.268451 | 0.007155 | Y |
| 44 | A9601_02251 | clpB2 | GGAACAATTGCTCT | -376 | 7.268442 | 0.007155 | Y |
| 45 | A9601_11241 | - | GCTACATATGTAAT | -73 | 7.255642 | 0.007434 | Y |
| 46 | A9601_16641 A9601_16651 | - glnB | TGGACATTTGTTTG | 13 | 7.247804 | 0.00769 | Y |
| 47 | A9601_17531 A9601_17521 A9601_17511 A9601_17501 A9601_17491 A9601_17481 A9601_17471 A9601_17461 | rplE rpsH rplF rplR rpsE rplO secY adk | GGTACTAGAGTCTT | -504 | 7.205927 | 0.00884 | Y |
| 48 | A9601_11411 | - | AGATCCCATGCTCT | -68 | 7.2049 | 0.00884 | Y |
| 49 | A9601_18441 A9601_18431 A9601_18421 A9601_18411 A9601_18401 A9601_18391 | gyrB - - - mgtE - | AGGACAAAAGATCT | 21 | 7.185836 | 0.009558 | Y |
| 50 | A9601_09771 | coaD | GGTACATTTGATCC | 32 | 7.183122 | 0.009558 | Y |

**Table S8**. Predicted LexA binding sites in *Prochlorococcus marinus* CCMP1375 genome at *P* < 0.01

| **Rank** | **Transcription Unit** | **Name** | **Putative LexA Binding Site** | **Position** | **Score** | **p-value** | **Have an orthologue or not(Y/N)** |
| --- | --- | --- | --- | --- | --- | --- | --- |
| 1 | Pro1716 | recA | CGTACGTGTGTACT | -91 | 9.00256 | 0 | Y |
| 2 | Pro0030 | wcaG | AATACATTTGTACT | -1 | 8.369374 | 1.50E-05 | Y |
| 3 | Pro0623 Pro0622 | - - | AGTATAAATGTACT | -95 | 8.106454 | 0.000228 | Y |
| 4 | Pro0703 Pro0702 | sbcD sbcC | AGTACTTAAGTACT | -230 | 7.877963 | 0.000655 | Y |
| 5 | Pro1218 Pro1219 | - - | AGTATATTTGTACT | -338 | 7.87099 | 0.000655 | Y |
| 6 | Pro1590 Pro1589 | groES groEL | AGAACAGTTGTAGT | -102 | 7.478763 | 0.002571 | Y |
| 7 | Pro0065 | - | CGAACCGCTGTCCG | -106 | 7.411073 | 0.00391 | Y |
| 8 | Pro0406 | - | AGTACCTAAGTAAT | -107 | 7.4059125 | 0.004297 | Y |
| 9 | Pro0031 | - | AGTACAAATGTATT | -18 | 7.382 | 0.005177 | N |

**Table S9**. Predicted LexA binding sites in *Prochlorococcus marinus* MED4 genome at *P* < 0.01

| **Rank** | **Transcription Unit** | **Name** | **Putative LexA Binding Site** | **Position** | **Score** | **p-value** | **Have an orthologue or not(Y/N)** |
| --- | --- | --- | --- | --- | --- | --- | --- |
| 1 | PMM1562 | recA | AGTACACATGTACT | -83 | 9.462387 | 0 | Y |
| 2 | PMM0029 PMM0028 | - pdxA | AATACATATGTACT | -14 | 8.49E+00 | 0 | Y |
| 3 | PMM0338 | - | AGTACAGATGTATT | -303 | 8.270617 | 0 | Y |
| 4 | PMM0334 | - | AGTATATTTGTACT | -17 | 7.901536 | 0.000246 | Y |
| 5 | PMM0053 | - | CGAACCGCTGTCCG | -96 | 7.885262 | 0.000284 | Y |
| 6 | PMM1251 PMM1250 | - - | AGTACTCGGGTTCT | -213 | 7.876558 | 0.000309 | Y |
| 7 | PMM1005 PMM1004 | - purT | AGAACAAAAGTTCT | -388 | 7.781509 | 0.000628 | Y |
| 8 | PMM1057 PMM1056 | cytM - | GGTACTATTGTTAT | -245 | 7.750383 | 0.000787 | Y |
| 9 | PMM0337 | - | AATACATCTGTACT | -271 | 7.736351 | 0.000985 | Y |
| 10 | PMM1570 | - | GCTACCAATGTACT | -224 | 7.705191 | 0.001252 | Y |
| 11 | PMM0936 PMM0937 PMM0938 | - umuC - | AGTATATATGTACT | -43 | 7.703486 | 0.001252 | Y |
| 12 | PMM0417 | - | AGCACCTCTGTAAT | -403 | 7.646904 | 0.001582 | Y |
| 13 | PMM0832 PMM0833 PMM0834 PMM0835 PMM0836 PMM0837 | dapB - ubiH - - - | ACTACACCTGTACT | 33 | 7.643746 | 0.001582 | Y |
| 14 | PMM1287 | - | GGAACACCTGCACT | -416 | 7.629234 | 0.001693 | Y |
| 15 | PMM1208 PMM1207 | gmd - | GGAACTACAGTACT | -352 | 7.603571 | 0.001769 | Y |
| 16 | PMM1117 PMM1116 PMM1115 PMM1114 | psbY gidA crtH - | AGAGCAATTGTAGT | 20 | 7.603517 | 0.001769 | Y |
| 17 | PMM1615 PMM1614 PMM1613 PMM1612 | ruvB - - - | AGAACAATTGCCAT | -12 | 7.465012 | 0.003052 | Y |
| 18 | PMM0503 PMM0504 | - - | TGTACACCAGGACT | -328 | 7.445594 | 0.003361 | Y |
| 19 | PMM1134 | - | AGTATAAATGTACC | -11 | 7.444884 | 0.003361 | Y |
| 20 | PMM0820 PMM0819 | - - | GGAACAATTGCATT | -13 | 7.431802 | 0.003514 | Y |
| 21 | PMM1568 | - | AGTACCAAAGTTAT | -699 | 7.427216 | 0.003743 | Y |
| 22 | PMM1462 PMM1463 | - glnB | TGGACATCTGTTTG | 13 | 7.405336 | 0.004037 | Y |
| 23 | PMM0195 | pgk | TGTACGTATGAACC | -194 | 7.354946 | 0.005345 | Y |
| 24 | PMM0870 PMM0869 PMM0868 | rpl33 rpsR - | GGTACAAGAGTTGT | 26 | 7.32888 | 0.006407 | Y |
| 25 | PMM0994 PMM0995 | - - | GGCACAATTGCCTT | -581 | 7.297845 | 0.007673 | Y |
| 26 | PMM0335 | - | AGGACATCAGTACG | -741 | 7.282628 | 0.007995 | Y |
| 27 | PMM0884 | coaD | GGTACATTTGATCC | 30 | 7.258758 | 0.009032 | Y |
| 28 | PMM0985 PMM0986 | - - | AGACCAATTGTCCT | -78 | 7.248555 | 0.009438 | Y |
| 29 | PMM0207 | clpB2 | GGAACTATTGCTCT | -397 | 7.244176 | 0.009438 | Y |

**Table S10**. Predicted LexA binding sites in *Prochlorococcus marinus* MIT9313 at *P* < 0.01

| **Rank** | **Transcription Unit** | **Name** | **Putative LexA Binding Site** | **Position** | **Score** | **p-value** | **Have an orthologue or not(Y/N)** |
| --- | --- | --- | --- | --- | --- | --- | --- |
| 1 | PMT1009 | - | AGTACAGGTGTACT | -12 | 9.855925 | 0 | Y |
| 2 | PMT2115 | - | AGTACAGGTGTACT | -4 | 9.723618 | 0 | Y |
| 3 | PMT0035 | - | AGTACAAATGTATT | 5 | 9.379964 | 0 | Y |
| 4 | PMT0303 | - | AGTACAGGTGTATT | -5 | 8.90789 | 7.11E-06 | Y |
| 5 | PMT1008 | - | AATACAGGTGTACT | -2 | 8.81065 | 2.37E-05 | Y |
| 6 | PMT1945 PMT1944 | - - | AGAACAGATGTTCT | -224 | 8.6578 | 0.000287 | Y |
| 7 | PMT0034 | - | AATACATTTGTACT | -1 | 8.629707 | 0.000351 | Y |
| 8 | PMT1010 | - | AGTACACCTGTACT | -502 | 8.550686 | 0.000455 | Y |
| 9 | PMT2116 | - | AGTACACCTGTACT | -265 | 8.510513 | 0.000467 | Y |
| 10 | PMT0380 PMT0381 | - - | GGTACACATGTATT | -16 | 8.334466 | 0.000545 | Y |
| 11 | PMT0834 PMT0833 | - - | AGAACATGAGTACT | -15 | 7.997634 | 0.001586 | Y |
| 12 | PMT0635 | - | GGAACGGGTGTACT | -8 | 7.884569 | 0.001946 | Y |
| 13 | PMT1383 | - | TGTACGCATGAATT | 26 | 7.859875 | 0.002155 | Y |
| 14 | PMT0614 | - | GGTACACATGTACT | -364 | 7.7936 | 0.002622 | N |
| 15 | PMT0638 | umuC | AGTATCTTTGTACT | -40 | 7.753157 | 0.002999 | Y |
| 16 | PMT1561 | - | GGTATGCCTGTATT | -462 | 7.73525 | 0.003212 | Y |
| 17 | PMT1661 PMT1660 PMT1659 | - hemK - | AGTTCATATGTACT | -69 | 7.64082 | 0.004229 | Y |
| 18 | PMT1043 | - | GCTACTGATGTCTT | -93 | 7.637025 | 0.004343 | Y |
| 19 | PMT1556 | - | AGTATGAAAGTACT | -276 | 7.532825 | 0.005545 | Y |
| 20 | PMT1578 | - | CGCACAGCTGTAGT | -505 | 7.52165 | 0.005718 | Y |
| 21 | PMT1814 PMT1815 | - gatC | AGAACCCATGTTCT | -69 | 7.471716 | 0.006617 | Y |
| 22 | PMT0975 | - | AATACAACTGTAAT | -256 | 7.451157 | 0.007032 | Y |
| 23 | PMT0222 | ydaO | AGTACGTAAGTAGT | -96 | 7.446752 | 0.00725 | Y |
| 24 | PMT0090 | - | AGTACAGCTGTAGC | -28 | 7.391948 | 0.008385 | Y |

**Table S11**. Predicted lexA binding sites in *Prochlorococcus marinus* MIT9211 genome at *P* < 0.01

| **Rank** | **Transcription Unit** | **Name** | **Putative LexA Binding Site** | **Position** | **Score** | **p-value** | **Have an orthologue or not(Y/N)** |
| --- | --- | --- | --- | --- | --- | --- | --- |
| 1 | P9211_16811 | recA | GGTACGTCTGTACT | -85 | 8.970605 | 0 | Y |
| 2 | P9211_00301 | - | AATACATTTGTACT | -1 | 8.324536 | 5.86E-05 | Y |
| 3 | P9211_08051 | - | AGTACAAGTGTACG | -12 | 8.211441 | 0.00017 | Y |
| 4 | P9211_07991 | - | AGCACAGATGTACT | -218 | 8.14E+00 | 0.000182 | Y |
| 5 | P9211_06221 P9211_06211 | - - | AGTATAGATGTACT | -95 | 8.12E+00 | 0.000188 | Y |
| 6 | P9211_12821 | - | GGTACAAGTGTTCT | -607 | 8.068769 | 0.000188 | Y |
| 7 | P9211_13401 P9211_13411 | fkpA - | GGTACCCCTGTTCT | -35 | 7.975684 | 0.000191 | Y |
| 8 | P9211_15451 | - | AGAACTTCTGTGCT | -43 | 7.709963 | 0.00057 | Y |
| 9 | P9211_06021 | - | AGTAGTTATGTACT | 7 | 7.638779 | 0.000651 | Y |
| 10 | P9211_15741 | - | AGTACTCGTGTTTT | -200 | 7.542123 | 0.000984 | Y |
| 11 | P9211_06911 | - | GGTACAGTTGTGAT | -36 | 7.459463 | 0.001795 | Y |
| 12 | P9211_15731 P9211_15721 P9211_15711 | - - atpH | AGTACTCGTGTTTT | -683 | 7.458401 | 0.001795 | Y |
| 13 | P9211_18381 P9211_18371 | purU - | AGTGCAGTTGTGCT | -161 | 7.391852 | 0.002865 | Y |
| 14 | P9211_03511 | fdx | AGTGCAAATGTTCT | -77 | 7.375902 | 0.003182 | Y |
| 15 | P9211_00311 | - | AGTACAAATGTATT | -17 | 7.3732 | 0.003182 | N |
| 16 | P9211_00331 | - | TGCACAAATGTAGT | -137 | 7.32895 | 0.003953 | Y |
| 17 | P9211_08941 P9211_08951 P9211_08961 | rpmG rpsR - | GGTACAAGGGTAGT | 24 | 7.321485 | 0.003953 | Y |
| 18 | P9211_07281 P9211_07291 P9211_07301 | hisS - - | TGGACAGGTGTATT | -29 | 7.316509 | 0.004132 | Y |
| 19 | P9211_00321 | - | TGCACAAATGTAGT | -736 | 7.309126 | 0.004366 | Y |
| 20 | P9211_00591 | - | CGAACCGCTGTCCG | -125 | 7.29485 | 0.004647 | Y |
| 21 | P9211_14181 | - | AGTACCTAAGTAAT | -210 | 7.259985 | 0.005803 | Y |
| 22 | P9211_17151 P9211_17141 P9211_17131 | ho1 pebA pebB | AGTTCTTTTGTCCT | -75 | 7.205996 | 0.007157 | Y |
| 23 | P9211_13471 P9211_13461 P9211_13451 P9211_13441 | - - lpd trpC | GGTACATGTGGCCT | -65 | 7.193124 | 0.007456 | Y |
| 24 | P9211_04451 P9211_04461 | ctaA cyoE | TGTACTCCTGTAAA | -124 | 7.180916 | 0.007767 | Y |
| 25 | P9211_07081 P9211_07071 P9211_07061 | umuD umuC - | AGTACTGATATACT | -18 | 7.179879 | 0.008094 | Y |
| 26 | P9211_00531 | ndk | AGAACAGTTGTTTT | -74 | 7.160877 | 0.008412 | Y |

**Table S12**. Predicted lexA binding sites in *Prochlorococcus marinus* MIT9215 genome at *P* < 0.01

| Rank | Transcription Unit | Name | Putative LexA Binding Site | Position | Score | p-value | Have an orthologue or not(Y/N) |
| --- | --- | --- | --- | --- | --- | --- | --- |
| 1 | P9215_07701 P9215_07691 | - - | AGTACATCTGTACT | -5 | 9.61075 | 0 | Y |
| 2 | P9215_18341 | recA | AGTACAGATGTACT | -86 | 9.44E+00 | 0 | Y |
| 3 | P9215_03671 | - | AGTACAAATGTATT | -10 | 9.219022 | 0 | Y |
| 4 | P9215_07741 P9215_07751 P9215_07761 | - - - | AGTACGCATGTATT | -10 | 9.120468 | 2.94E-06 | Y |
| 5 | P9215_07821 | - | AGTACAACTGTATT | -8 | 9.012368 | 2.94E-06 | Y |
| 6 | P9215_07441 | - | CGTACTTTTGTCCT | -325 | 8.852678 | 2.94E-06 | Y |
| 7 | P9215_00281 P9215_00271 | wcaG pdxA | AATACATATGTACT | -13 | 8.473593 | 0.000247 | Y |
| 8 | P9215_13321 P9215_13311 | - - | AATACACTTGTACT | -105 | 8.389866 | 0.00025 | Y |
| 9 | P9215_13331 P9215_13341 P9215_13351 P9215_13361 | - - - - | AGTACAAGTGTATT | -461 | 8.054848 | 0.000543 | Y |
| 10 | P9215_07801 | - | AATACAGTTGTACT | -545 | 7.978265 | 0.00079 | Y |
| 11 | P9215_19401 | - | AGGACTAGCGTCCT | -114 | 7.91963 | 0.000993 | Y |
| 12 | P9215_11061 P9215_11071 | - - | GGCACAATTGCCCT | -581 | 7.896807 | 0.001043 | Y |
| 13 | P9215_07811 | - | AATACAGTTGTACT | -171 | 7.873764 | 0.001098 | Y |
| 14 | P9215_12531 P9215_12521 P9215_12511 P9215_12501 | - gid crtH - | AGAACAATCGTAGT | 20 | 7.864894 | 0.001125 | Y |
| 15 | P9215_00641 | - | CGAACCGCTGTCCG | -95 | 7.811967 | 0.001286 | Y |
| 16 | P9215_16121 | - | TGTACAGCTGTCAA | -17 | 7.786911 | 0.001386 | Y |
| 17 | P9215_02981 | - | AGTACGTTAGTATT | -231 | 7.737254 | 0.001668 | Y |
| 18 | P9215_11121 | - | TTTACACCTGTAAT | -568 | 7.706045 | 0.001847 | Y |
| 19 | P9215_18421 | - | GCTACTAATGTACT | -222 | 7.705196 | 0.001847 | Y |
| 20 | P9215_18891 P9215_18881 P9215_18871 P9215_18861 | ruvB - - - | AGAACAATTGCAAT | -12 | 7.7 | 0.0019 | Y |
| 21 | P9215_02631 | - | CGTACTATTGTCAT | -57 | 7.672587 | 0.001968 | Y |
| 22 | P9215_03591 | - | AGTACATATATACT | -14 | 7.601139 | 0.0022 | Y |
| 23 | P9215_18301 P9215_18291 P9215_18281 P9215_18271 P9215_18261 P9215_18251 P9215_18241 | rplC rplD rplW rplB rpsS rplV rpsC | TGTACAATTGTAAC | -215 | 7.590866 | 0.002241 | Y |
| 24 | P9215_01061 | - | AGTCCGAATGGATT | -411 | 7.5182 | 0.002593 | Y |
| 25 | P9215_02431 | - | GGAACAACTGCACC | -424 | 7.513718 | 0.002593 | Y |
| 26 | P9215_14621 P9215_14611 P9215_14601 P9215_14591 | - - - - | ATTACTATTGTACT | -31 | 7.509193 | 0.002661 | Y |
| 27 | P9215_04531 P9215_04541 P9215_04551 P9215_04561 P9215_04571 P9215_04581 | vsr - - - - - | GGTACGTGGGTATG | -98 | 7.507971 | 0.002661 | Y |
| 28 | P9215_12771 P9215_12761 P9215_12751 | - - - | GCTACAAATGTTCT | -417 | 7.48395 | 0.002834 | Y |
| 29 | P9215_11041 | - | AGTTCAATTGTATT | -111 | 7.424995 | 0.003709 | Y |
| 30 | P9215_15681 P9215_15671 P9215_15661 P9215_15651 P9215_15641 P9215_15631 P9215_15621 | purC - lpxC fabZ lpxA lpxB msrA | AGTACTTTAGTAAT | -20 | 7.402601 | 0.0041 | Y |
| 31 | P9215_04501 | - | AGTATATTTGTATT | -63 | 7.3955 | 0.004297 | Y |
| 32 | P9215_01701 | metF | AGTACACTTGTTAA | -21 | 7.362474 | 0.00506 | Y |
| 33 | P9215_09541 P9215_09531 P9215_09521 | umuD umuC - | AGTACATATATACT | -13 | 7.356018 | 0.005369 | Y |
| 34 | P9215_08411 P9215_08421 P9215_08431 P9215_08441 P9215_08451 | pcyA - - - - | AGATCATATGTTCT | -198 | 7.331451 | 0.005971 | Y |
| 35 | P9215_15151 | - | ATTACTCCTGTGCT | -338 | 7.312498 | 0.006596 | Y |
| 36 | P9215_17641 | - | TGAACCAATGACCT | -96 | 7.283205 | 0.007474 | Y |
| 37 | P9215_02931 | - | GGTACTCCTGAAAT | -235 | 7.272255 | 0.007712 | Y |
| 38 | P9215_05051 | sun | AGTACGGATGTTAA | -414 | 7.256647 | 0.008164 | Y |
| 39 | P9215_17301 P9215_17311 | - glnB | TGGACATTTGTTTA | 37 | 7.219753 | 0.008993 | Y |
| 40 | P9215_13161 | - | TGGACGCATGACAT | -39 | 7.212813 | 0.008993 | Y |
| 41 | P9215_19001 | - | GGAACGGATGTTTT | -62 | 7.204336 | 0.009216 | Y |
| 42 | P9215_17861 | spr | GGTCCACTTGTGGT | -350 | 7.197615 | 0.009413 | Y |

**Table S13**. Predicted LexA binding sites in *Prochlorococcus marinus* MIT9301 genome at *P* < 0.01

| **Rank** | **Transcription Unit** | **Name** | **Putative LexA Binding Site** | **Position** | **Score** | **p-value** | **Have an orthologue or not(Y/N)** |
| --- | --- | --- | --- | --- | --- | --- | --- |
| 1 | P9301_17531 | recA | AGTACAGATGTACT | -85 | 9.453479 | 0 | Y |
| 2 | P9301_00291 | - | AGTACATATGTATT | -7 | 9.368433 | 0 | Y |
| 3 | P9301_03601 | - | AGTACTTATGTACT | 7 | 9.363882 | 0 | Y |
| 4 | P9301_03671 | - | AGTACAGATGTATT | -10 | 9.30E+00 | 0 | Y |
| 5 | P9301_07371 P9301_07381 P9301_07391 | - - - | AGTACACATGTATT | -10 | 9.23E+00 | 0 | Y |
| 6 | P9301_16181 | - | AGTACACTTGTACT | -560 | 8.83E+00 | 0 | Y |
| 7 | P9301_13071 | - | AGTACAAGTGTATT | -32 | 8.819941 | 0 | Y |
| 8 | P9301_00281 P9301_00271 | - pdxA | AATACATATGTACT | -13 | 8.473889 | 3.12E-06 | Y |
| 9 | P9301_07451 | - | AGTACAAACGTATT | -8 | 8.401351 | 6.23E-06 | Y |
| 10 | P9301_13061 | - | AATACACTTGTACT | -105 | 8.389678 | 6.23E-06 | Y |
| 11 | P9301_04481 P9301_04491 | - - | AGTACGGATGTTAG | -26 | 8.230091 | 1.56E-05 | Y |
| 12 | P9301_12861 | - | AGGACCTCTGTTCT | -66 | 8.033466 | 0.000265 | Y |
| 13 | P9301_10761 P9301_10771 | - - | GGCACAATTGCCCT | -581 | 7.918277 | 0.000527 | Y |
| 14 | P9301_12241 P9301_12231 P9301_12221 P9301_12211 | - gid crtH - | AGAACAATCGTAGT | 32 | 7.883175 | 0.000608 | Y |
| 15 | P9301_00621 | - | CGAACCGCTGTCCG | -96 | 7.832937 | 0.000723 | Y |
| 16 | P9301_11281 P9301_11291 | - - | AATACTTCTGTTCT | 24 | 7.805056 | 0.000782 | Y |
| 17 | P9301_15691 | - | TGTACAGCTGTCAA | -42 | 7.800021 | 0.000782 | Y |
| 18 | P9301_07441 | - | AATACGTTTGTACT | -173 | 7.74818 | 0.000913 | Y |
| 19 | P9301_17611 | - | GCTACTAATGTACT | -222 | 7.705592 | 0.001 | Y |
| 20 | P9301_10831 | - | TTTACACCTGTAAT | -599 | 7.704557 | 0.001 | Y |
| 21 | P9301_05291 | - | AGTACCCTTGTAAA | -505 | 7.664513 | 0.001112 | Y |
| 22 | P9301_17491 P9301_17481 P9301_17471 P9301_17461 P9301_17451 P9301_17441 P9301_17431 P9301_17421 P9301_17411 P9301_17401 P9301_17391 P9301_17381 | rplC rplD rplW rplB rpsS rplV rpsC rplP rpmC rpsQ rplN rplX | TGTACAATTGTAAC | -218 | 7.648536 | 0.001159 | Y |
| 23 | P9301_10941 P9301_10931 | - purT | AGAACAGTAGTATT | -163 | 7.618479 | 0.001237 | Y |
| 24 | P9301_00851 | - | GGAACAACTGTACC | -602 | 7.604257 | 0.001265 | Y |
| 25 | P9301_13051 P9301_13041 | - - | GGTACTTTTGAATT | 23 | 7.60272 | 0.001265 | Y |
| 26 | P9301_03611 | - | AGTACATAAGTACT | -71 | 7.5601 | 0.001436 | N |
| 27 | P9301_02441 | - | GGAACAACTGCACC | -429 | 7.540668 | 0.001533 | Y |
| 28 | P9301_01051 | - | AGTCCGAATGGATT | -411 | 7.5293 | 0.001673 | Y |
| 29 | P9301_12891 | - | TGGACGCATGCAAT | -39 | 7.482954 | 0.002128 | Y |
| 30 | P9301_18571 | - | AGGACTAGCGTCCC | -115 | 7.466431 | 0.002409 | Y |
| 31 | P9301_16521 P9301_16531 | - glnB | TGGACATTTGTTTG | 13 | 7.376455 | 0.003801 | Y |
| 32 | P9301_01721 | metF | AGTACACTTGTTAA | -21 | 7.374255 | 0.003801 | Y |
| 33 | P9301_09221 P9301_09211 P9301_09201 | umuD umuC - | AGTACATATATACT | -13 | 7.350294 | 0.004206 | Y |
| 34 | P9301_14971 | hemN | AGTGCTTATGTGCA | 32 | 7.332359 | 0.004633 | Y |
| 35 | P9301_11521 | - | TGGACAACAGTTCT | -499 | 7.324758 | 0.004814 | Y |
| 36 | P9301_10071 | - | AGCACGTTTGTAAA | -58 | 7.317738 | 0.004985 | Y |
| 37 | P9301_15341 | spoIID | GGGACACCTGAGCT | -112 | 7.314429 | 0.004985 | Y |
| 38 | P9301_02271 | clpB2 | GGAACAATTGCTCT | -371 | 7.293074 | 0.005403 | Y |
| 39 | P9301_02921 | - | GGTACTCCTGAAAT | -235 | 7.288788 | 0.005593 | Y |
| 40 | P9301_08751 | - | GGTACTAAAGAACT | -67 | 7.287697 | 0.005593 | Y |
| 41 | P9301_07891 | - | AGCACTTATGTATA | -155 | 7.286368 | 0.005593 | Y |
| 42 | P9301_10951 | btuE | AATACTACTGTTCT | 30 | 7.265338 | 0.005939 | Y |
| 43 | P9301_11231 | - | GCTACATATGTAAT | -73 | 7.257892 | 0.006132 | Y |
| 44 | P9301_17591 | - | TGTACTAAAGTTAT | -700 | 7.251617 | 0.006132 | Y |
| 45 | P9301_11421 | - | AGATCCCATGCTCT | -68 | 7.224475 | 0.006777 | Y |
| 46 | P9301_09751 | coaD | GGTACATTTGATCC | 32 | 7.208664 | 0.007238 | Y |
| 47 | P9301_08711 | - | AGTTCTAGTGAACT | -488 | 7.193168 | 0.007528 | Y |
| 48 | P9301_02331 P9301_02321 | - - | ATTACTGATGTACC | -193 | 7.189664 | 0.00784 | Y |
| 49 | P9301_17371 P9301_17361 P9301_17351 P9301_17341 P9301_17331 P9301_17321 P9301_17311 P9301_17301 | rplE rpsH rplF rplR rpsE rplO secY adk | GGTACTAGAGTTTT | -505 | 7.188982 | 0.00784 | Y |
| 50 | P9301_17101 | - | GGTCCACTTGTTGT | -350 | 7.1835 | 0.00784 | Y |
| 51 | P9301_04981 | gltX | GGGACCGTAGTTCA | -77 | 7.166228 | 0.008544 | Y |
| 52 | P9301_18251 P9301_18241 | gyrB - | AGGACAAAAGATCT | 21 | 7.153969 | 0.008905 | Y |
| 53 | P9301_02341 | - | AGACCTAGTGTAGT | -759 | 7.152868 | 0.008905 | Y |
| 54 | P9301_06491 P9301_06501 P9301_06511 | hisS - - | GGAACAGTAGACCT | 36 | 7.142493 | 0.009344 | Y |

**Table S14**. Predicted LexA binding sites in *Prochlorococcus marinus* MIT 9303 genome at *P* < 0.01

| **Rank** | **Transcription Unit** | **Name** | **Putative LexA Binding Site** | **Position** | **Score** | **p-value** | **Have an orthologue or not(Y/N)** |
| --- | --- | --- | --- | --- | --- | --- | --- |
| 1 | P9303_10871 P9303_10881 | - - | AGTACAGGTGTACT | -14 | 9.85495 | 0 | Y |
| 2 | P9303_00351 | - | AGTACAAATGTATT | 5 | 9.49285 | 0 | Y |
| 3 | P9303_28121 | - | AGTACGATTGTACT | -14 | 9.160465 | 0 | Y |
| 4 | P9303_10911 | - | AATACAGGTGTACT | 0 | 8.814475 | 0 | Y |
| 5 | P9303_25941 P9303_25931 P9303_25921 P9303_25911 | - kefB - galM | AGAACAGATGTTCT | -224 | 8.656075 | 0 | Y |
| 6 | P9303_00341 | - | AATACATTTGTACT | -1 | 8.635032 | 1.77E-06 | Y |
| 7 | P9303_19141 | lexA | GGTACACATGTATT | -41 | 8.280912 | 0.000106 | Y |
| 8 | P9303_28101 | - | AGTACGATTGTACT | -340 | 8.22E+00 | 0.000186 | Y |
| 9 | P9303_16341 | - | AGAACAAGTGTACT | -15 | 8.16E+00 | 0.000257 | Y |
| 10 | P9303_12451 | - | GGTACAAATGTAAT | -30 | 8.15E+00 | 0.000257 | Y |
| 11 | P9303_13691 P9303_13701 P9303_13711 | - - - | AGAACATAAGTACT | -17 | 8.05E+00 | 0.000386 | Y |
| 12 | P9303_16001 | umuD | GGAACAGGTGTACT | -23 | 8.036029 | 0.000416 | Y |
| 13 | P9303_16271 | - | AGTACATGTGTACC | -435 | 7.917686 | 0.000757 | Y |
| 14 | P9303_05941 | - | TGTACGCATGAATT | -77 | 7.855375 | 0.000994 | Y |
| 15 | P9303_15961 | umuC | AGTGCGTCTGTACT | -38 | 7.798749 | 0.001249 | Y |
| 16 | P9303_16261 | - | GGTACACATGTACT | -122 | 7.7919 | 0.001249 | N |
| 17 | P9303_00561 P9303_00551 | - - | AGTACACCTGTACG | -550 | 7.773625 | 0.001349 | Y |
| 18 | P9303_21921 | - | AGTACGCTCGTATT | -58 | 7.734737 | 0.00159 | Y |
| 19 | P9303_03591 | - | GGTATGCCTGTATT | -273 | 7.728125 | 0.001653 | Y |
| 20 | P9303_10181 | - | GCTACTGATGTCTT | -91 | 7.638975 | 0.002465 | Y |
| 21 | P9303_03361 | - | AGTACGCATGCACT | -431 | 7.534315 | 0.003628 | Y |
| 22 | P9303_15651 | - | ATTACATTTGTATT | -49 | 7.528954 | 0.003752 | Y |
| 23 | P9303_03751 P9303_03761 | - - | AGTATGAAAGTACT | -75 | 7.5263 | 0.003752 | Y |
| 24 | P9303_10921 P9303_10931 | - - | AGTACACCTGTATT | -10 | 7.524 | 0.003752 | N |
| 25 | P9303_15461 | - | TGTACGTCTGTACA | -153 | 7.523681 | 0.003752 | Y |
| 26 | P9303_12461 | - | ATTACATTTGTACC | -262 | 7.4714 | 0.004602 | Y |
| 27 | P9303_20181 | - | AGTACAGACGTATT | -767 | 7.468264 | 0.004781 | Y |
| 28 | P9303_21241 | - | AGTACGTAAGTAGT | -94 | 7.441033 | 0.005187 | Y |
| 29 | P9303_12861 | - | AGTACCTATGTCCT | -334 | 7.4395 | 0.005396 | N |
| 30 | P9303_16131 | - | AGTACGTATGAATT | -564 | 7.417292 | 0.0058 | Y |
| 31 | P9303_15661 | modF | AATACAAATGTAAT | -354 | 7.364077 | 0.006844 | Y |
| 32 | P9303_19581 P9303_19571 | - - | GGCACATTTGTCCT | -59 | 7.362321 | 0.006844 | Y |
| 33 | P9303_28741 P9303_28751 P9303_28761 P9303_28771 P9303_28781 P9303_28791 | - - - - - - | AGTACAAGGGTGTT | -78 | 7.326642 | 0.007813 | Y |
| 34 | P9303_09001 | - | AATACAGCCGTACT | -103 | 7.322764 | 0.007813 | Y |
| 35 | P9303_16521 P9303_16511 P9303_16501 | - - - | TGTACCGGTGGACT | -232 | 7.320038 | 0.007813 | Y |
| 36 | P9303_15971 | - | AGTACAGACGCACT | -64 | 7.277832 | 0.009321 | Y |

**Table S15**. Predicted LexA binding sites in *Prochlorococcus marinus* MIT 9312 genome at *P* < 0.01

| **Rank** | **Transcription Unit** | **Name** | **Putative LexA Binding Site** | **Position** | **Score** | **p-value** | **Have an orthologue or not(Y/N)** |
| --- | --- | --- | --- | --- | --- | --- | --- |
| 1 | PMT9312_1654 | - | AGTACAGATGTACT | -86 | 9.458231 | 0 | Y |
| 2 | PMT9312_0030 | - | AGTACATATGTATT | -7 | 9.369288 | 0 | Y |
| 3 | PMT9312_0693 | - | AGTACAAATGTATT | -9 | 9.07655 | 0 | Y |
| 4 | PMT9312_0697 PMT9312_0698 | - - | AGTACAACTGTATT | -8 | 8.83661 | 0 | Y |
| 5 | PMT9312_0029 PMT9312_0028 | - - | AATACATATGTACT | -13 | 8.475565 | 0 | Y |
| 6 | PMT9312_1227 | - | AGTACAAACGTATT | -32 | 8.43E+00 | 0 | Y |
| 7 | PMT9312_1226 | - | AATACGTTTGTACT | -105 | 8.351627 | 3.29E-06 | Y |
| 8 | PMT9312_0344 | - | AGTACAGATGTATT | -306 | 8.258259 | 6.58E-06 | Y |
| 9 | PMT9312_0696 PMT9312_0695 | - - | AATACAGTTGTACT | -169 | 7.979975 | 0.000645 | Y |
| 10 | PMT9312_1759 | - | AGGACTAGCGTCCT | -115 | 7.94547 | 0.000701 | Y |
| 11 | PMT9312_0054 | - | CGAACCGCTGTCCG | -97 | 7.839762 | 0.000767 | Y |
| 12 | PMT9312_0343 PMT9312_0342 | - - | AATACATCTGTACT | -275 | 7.770255 | 0.000846 | Y |
| 13 | PMT9312_1708 PMT9312_1707 PMT9312_1706 PMT9312_1705 | - - - - | AGAACAATTGCAAT | -12 | 7.724375 | 0.000928 | Y |
| 14 | PMT9312_1662 | - | GCTACCAATGTACT | -222 | 7.689085 | 0.00099 | Y |
| 15 | PMT9312_0503 | - | AGTACTCTTGTAAA | -512 | 7.647871 | 0.001099 | Y |
| 16 | PMT9312_1650 PMT9312_1649 PMT9312_1648 PMT9312_1647 PMT9312_1646 PMT9312_1645 PMT9312_1644 PMT9312_1643 PMT9312_1642 PMT9312_1641 PMT9312_1640 PMT9312_1639 | - - - - - - - - - - - - | TGTACAATTGTAAC | -219 | 7.634924 | 0.001139 | Y |
| 17 | PMT9312_1159 PMT9312_1160 | - - | AGTTCAAATGTCTT | 5 | 7.617484 | 0.001204 | Y |
| 18 | PMT9312_1502 | - | CTTACAAATGTATT | 7 | 7.606429 | 0.001227 | Y |
| 19 | PMT9312_1698 | - | AGAACAGCTGCACT | -134 | 7.583934 | 0.001323 | Y |
| 20 | PMT9312_0284 PMT9312_0283 | - - | GGTACTTTTGTATA | -26 | 7.502543 | 0.002053 | Y |
| 21 | PMT9312_1013 | - | AATACTAAAGTACT | -223 | 7.491912 | 0.002165 | Y |
| 22 | PMT9312_1225 | - | GGTACTTTTGAATT | -192 | 7.464244 | 0.002705 | Y |
| 23 | PMT9312_0232 PMT9312_0231 | - - | TGAACATATGAACT | -68 | 7.437776 | 0.003333 | Y |
| 24 | PMT9312_1446 | - | GGTACTCCAGAACT | -112 | 7.417752 | 0.003873 | Y |
| 25 | PMT9312_1616 PMT9312_1615 | - - | GGAACATTTGTTAT | -611 | 7.394954 | 0.004498 | Y |
| 26 | PMT9312_0560 PMT9312_0561 PMT9312_0562 | - - - | AGTACATTTGTTGA | -496 | 7.385808 | 0.004788 | Y |
| 27 | PMT9312_0155 | - | AGTACACTTGTTAA | -21 | 7.379601 | 0.005117 | Y |
| 28 | PMT9312_1176 | - | AGAACCAATGTTAT | -92 | 7.350833 | 0.005742 | Y |
| 29 | PMT9312_0863 PMT9312_0862 PMT9312_0861 | - - - | AGTACATATATACT | -13 | 7.350491 | 0.005742 | Y |
| 30 | PMT9312_1408 | - | AGTGCTTATGTGCA | 32 | 7.339586 | 0.006334 | Y |
| 31 | PMT9312_0209 | - | GGCACAATTGCTCT | -379 | 7.328908 | 0.006561 | Y |
| 32 | PMT9312_0306 PMT9312_0305 | - - | TGAACTTATGAACT | -78 | 7.328163 | 0.006561 | Y |
| 33 | PMT9312_1476 | - | AGGACTCCTGTCTT | -682 | 7.316543 | 0.006861 | Y |
| 34 | PMT9312_1588 | - | TGAACCAATGACCT | -97 | 7.31096 | 0.006861 | Y |
| 35 | PMT9312_0271 | - | GGTACTCCTGAAAT | -232 | 7.29453 | 0.007526 | Y |
| 36 | PMT9312_1001 PMT9312_1002 | - - | AGCACAATTGCCTT | -566 | 7.277855 | 0.00817 | Y |
| 37 | PMT9312_1555 PMT9312_1556 | - - | TGGACATTTGTTTG | 13 | 7.270748 | 0.00817 | Y |
| 38 | PMT9312_1119 PMT9312_1120 PMT9312_1121 PMT9312_1122 | - - - - | GGTACAAGTGTTAC | -366 | 7.270356 | 0.00817 | Y |
| 39 | PMT9312_1128 PMT9312_1127 PMT9312_1126 PMT9312_1125 | - - - - | AGAGCTATTGTCGT | 20 | 7.256064 | 0.008901 | Y |
| 40 | PMT9312_1638 PMT9312_1637 PMT9312_1636 PMT9312_1635 PMT9312_1634 PMT9312_1633 PMT9312_1632 PMT9312_1631 PMT9312_1815 PMT9312_1630 PMT9312_1629 PMT9312_1628 PMT9312_1627 PMT9312_1626 PMT9312_1625 PMT9312_1624 PMT9312_1623 PMT9312_1622 | - - - - - - - - rpl36 - - - - - - - - - | GGTACTAGAGTTTT | -504 | 7.230361 | 0.009576 | Y |

**Table S16** Predicted LexA binding sites in *Prochlorococcus marinus* MIT 9515 genome at *P* < 0.01

| **Rank** | **Transcription Unit** | **Name** | **Putative LexA Binding Site** | **Position** | **Score** | **p-value** | **Have an orthologue or not(Y/N)** |
| --- | --- | --- | --- | --- | --- | --- | --- |
| 1 | P9515_16041 | - | AGTACTTTTGTACT | -11 | 9.473717 | 0 | Y |
| 2 | P9515_17441 | recA | AGTACGCATGTACT | -83 | 9.442976 | 0 | Y |
| 3 | P9515_15641 | - | AGTACAAATGTATT | -396 | 9.213833 | 0 | Y |
| 4 | P9515_04311 P9515_04301 | - - | AGTACAAGTGTATT | -9 | 8.99063 | 0 | Y |
| 5 | P9515_00291 | - | AGTACATATGTATT | -2 | 8.942755 | 0 | Y |
| 6 | P9515_12911 | - | AGTACATATGTATT | -11 | 8.923024 | 0 | Y |
| 7 | P9515_00281 P9515_00271 | - pdxA | AATACATATGTACT | -14 | 8.50E+00 | 0 | Y |
| 8 | P9515_12901 | - | AATACATATGTACT | -108 | 8.39E+00 | 3.06E-06 | Y |
| 9 | P9515_03661 | - | AGTATATTTGTACT | -16 | 7.915649 | 0.000729 | Y |
| 10 | P9515_00611 | - | CGAACCGCTGTCCG | -96 | 7.911474 | 0.000729 | Y |
| 11 | P9515_10331 P9515_10321 P9515_10311 | - - - | GGTGCACTTGTTCA | -40 | 7.886279 | 0.000778 | Y |
| 12 | P9515_14651 P9515_14641 | codA - | TGTACAAGTGTTAT | -467 | 7.856634 | 0.000818 | Y |
| 13 | P9515_12081 P9515_12071 P9515_12061 P9515_12051 | psbY gid crtH - | AGAACTCTTGTCGT | 20 | 7.813343 | 0.000907 | Y |
| 14 | P9515_02361 | clpB2 | GGTACTATTGCTCT | -400 | 7.748516 | 0.001023 | Y |
| 15 | P9515_17571 | - | GCTACTAATGTACT | -220 | 7.736321 | 0.001042 | Y |
| 16 | P9515_06881 P9515_06891 P9515_06901 | hisS - - | GGAACAGTTGATCT | 36 | 7.718649 | 0.001063 | Y |
| 17 | P9515_14481 P9515_14471 P9515_14461 P9515_14451 | - ffh rpsP phoH | GGGACACCTGCACT | -416 | 7.702137 | 0.001097 | Y |
| 18 | P9515_12481 | - | ATTACACATGTATT | 5 | 7.671957 | 0.001232 | Y |
| 19 | P9515_08941 | - | AGTTCAAATGTCTT | -230 | 7.656596 | 0.001296 | Y |
| 20 | P9515_15651 P9515_15661 | - - | AATACATTTGTACT | -215 | 7.64633 | 0.001342 | Y |
| 21 | P9515_02741 | - | GGTACAGATGGAAT | 29 | 7.513922 | 0.002359 | Y |
| 22 | P9515_02431 | - | TGAACCAGCGTTCT | -535 | 7.50992 | 0.002426 | Y |
| 23 | P9515_18041 P9515_18031 P9515_18021 P9515_18011 | ruvB - - - | AGAACAATTGCCAT | -12 | 7.484137 | 0.002629 | Y |
| 24 | P9515_17551 | - | AGTACCAAAGTTAT | -698 | 7.447907 | 0.0031 | Y |
| 25 | P9515_04351 | - | AGTATATTTGTATT | -62 | 7.40635 | 0.003649 | Y |
| 26 | P9515_09411 P9515_09401 | - - | TGAACTTCTGCACT | -108 | 7.40454 | 0.003649 | Y |
| 27 | P9515_07001 | - | AGGACCTATGTTTT | -88 | 7.404511 | 0.003649 | Y |
| 28 | P9515_04631 P9515_04641 P9515_04651 | lipB fadD - | TGTTCATTTGTATT | -385 | 7.361102 | 0.004344 | Y |
| 29 | P9515_04321 | - | AATACACTTGTACT | -148 | 7.306518 | 0.005533 | Y |
| 30 | P9515_03011 | - | TGAACTATTGTCCA | -131 | 7.297135 | 0.00579 | Y |
| 31 | P9515_09661 | coaD | GGTACATTTGATCC | 30 | 7.283904 | 0.006005 | Y |
| 32 | P9515_05671 P9515_05681 | cobK cutA | TGTCCTGCTGGACT | -328 | 7.281597 | 0.006005 | Y |
| 33 | P9515_03711 | - | AGTATTTATGTACT | -218 | 7.281376 | 0.006005 | Y |
| 34 | P9515_10901 P9515_10891 | - purT | AGAACAGGGGTGTT | -169 | 7.278216 | 0.006265 | Y |
| 35 | P9515_03211 | chlD | AGGACAAGTGTGTT | -302 | 7.268261 | 0.00655 | Y |
| 36 | P9515_06241 P9515_06251 P9515_06261 | hisG - - | AGTTCTCTTGTCCC | -236 | 7.267213 | 0.00655 | Y |
| 37 | P9515_04221 | - | CGGACGCATGACAT | -38 | 7.259627 | 0.006838 | Y |
| 38 | P9515_05371 | gltX | GGGACCGTAGTTCA | -33 | 7.247804 | 0.007154 | Y |
| 39 | P9515_16101 | - | GGAACTCTTGCTCT | 30 | 7.242632 | 0.007154 | Y |
| 40 | P9515_10881 | - | AATACTAAAGTTCT | -229 | 7.236357 | 0.007558 | Y |
| 41 | P9515_16411 P9515_16421 | - glnB | TGGACATCTGTTTA | 13 | 7.232611 | 0.007558 | Y |
| 42 | P9515_03721 | - | AGTATTCATGTACT | -319 | 7.219407 | 0.008333 | Y |
| 43 | P9515_04181 | - | AGTACTTAAGTCGT | -486 | 7.200095 | 0.008759 | Y |

**Table S17**. Predicted LexA binding sites in *Prochlorococcus marinus* NATL1A genome at *P* < 0.01

| **Rank** | **Transcription Unit** | **Name** | **Putative LexA Binding Site** | **Position** | **Score** | **p-value** | **Have an orthologue or not(Y/N)** |
| --- | --- | --- | --- | --- | --- | --- | --- |
| 1 | NATL1_11901 | - | AGTACATGTGTACT | -55 | 9.476688 | 0 | Y |
| 2 | NATL1_00291 | - | AGTACAAATGTATT | 5 | 9.2426 | 0 | Y |
| 3 | NATL1_20071 | recA | CGTACGTCTGTACT | -92 | 9.110663 | 0 | Y |
| 4 | NATL1_11911 NATL1_11921 | - - | AGTACACATGTACT | -677 | 8.823519 | 0 | Y |
| 5 | NATL1_01031 | - | AGTACACTAGTATT | -206 | 8.417225 | 7.66E-05 | Y |
| 6 | NATL1_10091 | - | TGGACTTCTGTAAT | 15 | 8.35715 | 7.90E-05 | Y |
| 7 | NATL1_00281 | - | AATACATTTGTACT | -1 | 8.240586 | 8.61E-05 | Y |
| 8 | NATL1_13741 | - | AGTAGGTCTGTACT | -203 | 8.20E+00 | 9.33E-05 | Y |
| 9 | NATL1_18281 | - | AGTACAATTGTATT | -421 | 8.086201 | 0.00011 | Y |
| 10 | NATL1_16261 NATL1_16271 | - - | AGTACACCAGTACC | -148 | 8.007098 | 0.000203 | Y |
| 11 | NATL1_07571 | - | GGCACTTGGGTCCT | -178 | 7.97968 | 0.000246 | Y |
| 12 | NATL1_17121 | lnt | AGTACCCTTGTAAT | -473 | 7.872433 | 0.000637 | Y |
| 13 | NATL1_18271 | - | AATACAATTGTACT | -17 | 7.855381 | 0.000766 | Y |
| 14 | NATL1_17241 NATL1_17231 | codA - | AGTACATGTGTTAT | -542 | 7.78465 | 0.001273 | Y |
| 15 | NATL1_01721 | - | AGCACTGATGTTCT | -296 | 7.70853 | 0.002039 | Y |
| 16 | NATL1_12661 | - | CTTACTCAGGTACT | -107 | 7.6828 | 0.00218 | Y |
| 17 | NATL1_11851 | - | AGTAGTTATGTACT | 7 | 7.546139 | 0.003326 | Y |
| 18 | NATL1_14421 NATL1_14431 NATL1_14441 | - - - | AGTACTTGAGTAAA | -62 | 7.526832 | 0.00351 | Y |
| 19 | NATL1_14291 NATL1_14301 | - - | AGTACTTTTGTTAG | -80 | 7.510441 | 0.003585 | Y |
| 20 | NATL1_01161 | - | CGAACCGCTGTCCG | -124 | 7.497622 | 0.003774 | Y |
| 21 | NATL1_01541 NATL1_01551 | - - | TGTACACTTGTATG | -97 | 7.494248 | 0.003774 | Y |
| 22 | NATL1_21841 NATL1_21831 | purU - | AGTTCATTTGTCCT | 39 | 7.488262 | 0.003836 | Y |
| 23 | NATL1_17441 NATL1_17431 | - - | TGTACTTGTTTACT | -220 | 7.4715 | 0.00392 | Y |
| 24 | NATL1_12991 | - | ATTACTTATGTTCA | -64 | 7.46875 | 0.004013 | Y |
| 25 | NATL1_12691 | - | AGTACGGGTGAAAA | -113 | 7.4234 | 0.004391 | Y |
| 26 | NATL1_04741 | lepA | AGTACTGGTGAGCT | -66 | 7.363779 | 0.004996 | Y |
| 27 | NATL1_19911 NATL1_19901 NATL1_19891 NATL1_19881 NATL1_19871 NATL1_19861 NATL1_19851 NATL1_19841 | rplE rpsH rplF rplR rpsE rplO secY adk | GGTACTAGGGTATT | -521 | 7.359127 | 0.00515 | Y |
| 28 | NATL1_13321 | - | ATTACATTTGTCAT | -27 | 7.357321 | 0.00515 | Y |
| 29 | NATL1_04191 | - | AGCACTGGCGTACT | -571 | 7.343159 | 0.005262 | Y |
| 30 | NATL1_08201 | - | GGCACTCTCGTACT | 16 | 7.33734 | 0.005413 | Y |
| 31 | NATL1_03011 | aspA | GGTACTGTTGCTCT | -429 | 7.336782 | 0.005413 | Y |
| 32 | NATL1_05171 NATL1_05161 NATL1_05151 NATL1_05141 | petC petA lgt cobM | TGAACGTCTGTAAT | 2 | 7.331877 | 0.005413 | Y |
| 33 | NATL1_04531 NATL1_04541 NATL1_04551 | lipB fadD - | TGTACTTACGTAAT | -250 | 7.324157 | 0.005561 | Y |
| 34 | NATL1_16811 NATL1_16801 | argF lexA | GGTACATATGGAAT | -342 | 7.309258 | 0.005903 | Y |
| 35 | NATL1_12981 | - | CGTGCTTATGTCCT | -22 | 7.305221 | 0.005903 | Y |
| 36 | NATL1_01051 | - | AGCACAGCTGTATC | -333 | 7.301798 | 0.005903 | Y |
| 37 | NATL1_12071 | - | AGTACGATTGGATT | -20 | 7.280451 | 0.006423 | Y |
| 38 | NATL1_13341 | - | CGTACTTATGACCT | -718 | 7.271257 | 0.006638 | Y |
| 39 | NATL1_07951 | glyS | GGTACTGAGGAACT | 36 | 7.24871 | 0.007327 | Y |
| 40 | NATL1_10251 | - | AGTACGTGAGTTAT | -349 | 7.224495 | 0.007856 | Y |
| 41 | NATL1_10121 NATL1_10131 NATL1_10141 | rpmG rpsR - | AGTAAGATTGTACT | -39 | 7.211854 | 0.008138 | Y |
| 42 | NATL1_07941 | sir | GGTACCGGTGTAGG | -5 | 7.210927 | 0.008138 | Y |
| 43 | NATL1_05661 NATL1_05651 | proS psb27 | CGTACAATTGGACA | -97 | 7.191946 | 0.008775 | Y |

**Table S18**. Predicted LexA binding sites in *Prochlorococcus marinus* NATL2A genome at *P* < 0.01

| **Rank** | **Transcription Unit** | **Name** | **Putative LexA Binding Site** | **Position** | **Score** | **p-value** | **Have an orthologue or not(Y/N)** |
| --- | --- | --- | --- | --- | --- | --- | --- |
| 1 | PMN2A_0471 | - | AGTACATGTGTACT | -55 | 9.468937 | 0 | Y |
| 2 | PMN2A_1357 | - | AGTACAAATGTATT | 5 | 9.241775 | 0 | Y |
| 3 | PMN2A_1133 | recA | CGTACGTCTGTACT | -92 | 9.119078 | 0 | Y |
| 4 | PMN2A_0472 PMN2A_0473 | - - | AGTACACATGTACT | -676 | 8.827965 | 0 | Y |
| 5 | PMN2A_1404 | - | AGTACACTAGTATT | -186 | 8.42E+00 | 0 | Y |
| 6 | PMN2A_0336 | - | TGGACTTCTGTAAT | 15 | 8.36E+00 | 0 | Y |
| 7 | PMN2A_0126 | - | GGTACTTGGGTCCT | -180 | 8.35E+00 | 0 | Y |
| 8 | PMN2A_1356 | - | AATACATTTGTACT | -1 | 8.25E+00 | 0 | Y |
| 9 | PMN2A_0828 | - | AGGACGAATGTACT | -12 | 8.238003 | 0 | Y |
| 10 | PMN2A_0589 | - | AGTAGGTCTGTACT | -203 | 8.1926 | 0 | Y |
| 11 | PMN2A_0413 | - | TGTTCTCTTGTCCT | -195 | 7.859475 | 0.001702 | Y |
| 12 | PMN2A_0449 | - | AGAACGGAAGTACT | -347 | 7.803971 | 0.00227 | Y |
| 13 | PMN2A_1469 | - | AGCACTGATGTTCT | -295 | 7.716252 | 0.003972 | Y |
| 14 | PMN2A_0504 | - | TGTACCTTTGTGAT | -666 | 7.635338 | 0.00539 | Y |
| 15 | PMN2A_1241 | ribH | CGCACTTTTGTTCT | -274 | 7.597436 | 0.005674 | Y |
| 16 | PMN2A_0467 | - | AGTAGTTATGTACT | 7 | 7.540695 | 0.006241 | Y |
| 17 | PMN2A_0616 PMN2A_0617 | - - | AGTACTTTTGTTAG | -79 | 7.512804 | 0.006241 | Y |
| 18 | PMN2A_1415 | - | CGAACCGCTGTCCG | -124 | 7.508008 | 0.006241 | Y |
| 19 | PMN2A_1311 PMN2A_1310 | - - | AGTTCATTTGTCCT | 39 | 7.500429 | 0.006241 | Y |
| 20 | PMN2A_1452 PMN2A_1453 | - - | TGTACACTTGTATG | -97 | 7.495036 | 0.006241 | Y |
| 21 | PMN2A_1040 | - | AGGACTTAAGTACT | -278 | 7.491004 | 0.006241 | Y |
| 22 | PMN2A_0379 PMN2A_0378 PMN2A_0377 PMN2A_0376 | - - - - | CGAACCTGTGTAAA | -116 | 7.484984 | 0.006241 | Y |
| 23 | PMN2A_0889 PMN2A_0888 | - - | TGTACTTGTTTACT | -144 | 7.477875 | 0.006241 | Y |
| 24 | PMN2A_0543 | - | ATTACTTATGTTCA | -64 | 7.475125 | 0.006241 | Y |
| 25 | PMN2A_0518 | - | AGTACCAAGGTAAA | -107 | 7.448157 | 0.006525 | Y |
| 26 | PMN2A_1682a | psaM | AGTACATTTGATCT | 38 | 7.441809 | 0.006525 | Y |
| 27 | PMN2A_1161 | - | TGTACTCTTGTCAT | -389 | 7.439266 | 0.007376 | Y |
| 28 | PMN2A_0619 PMN2A_0620 | - - | TGTACTTGAGTAAA | -62 | 7.43323 | 0.007376 | Y |
| 29 | PMN2A_0528 | - | AGTACGGGTGAAAA | -64 | 7.4231 | 0.00766 | Y |
| 30 | PMN2A_0131 | - | AGTTCAATTGTTCT | -10 | 7.382445 | 0.008794 | Y |
| 31 | PMN2A_1754 | - | AGTACTGGTGAGCT | -66 | 7.371501 | 0.008794 | Y |
| 32 | PMN2A_0947 | - | AGTCCTATTGTTCT | -652 | 7.365664 | 0.009078 | Y |
| 33 | PMN2A_0565 | - | ATTACATTTGTTAT | -27 | 7.349413 | 0.009645 | Y |
| 34 | PMN2A_1591 | - | GGTACTGTTGCTCT | -429 | 7.346183 | 0.009645 | Y |
| 35 | PMN2A_0188 | - | GGCACTCTCGTACT | 16 | 7.34434 | 0.009645 | Y |
| 36 | PMN2A_1736 PMN2A_1737 PMN2A_1738 | - - - | TGTACTTACGTAAT | -250 | 7.325147 | 0.009929 | Y |

**Table S19**. Predicted LexA binding sites in *Synechococcus* CC9311 genome at *P* < 0.01

| **Rank** | **Transcription Unit** | **Name** | **Putative LexA Binding Site** | **Position** | **Score** | **p-value** | **Have an orthologue or not(Y/N)** |
| --- | --- | --- | --- | --- | --- | --- | --- |
| 1 | sync_1623 | - | AGTACAGGTGTATT | -32 | 9.130074 | 0 | Y |
| 2 | sync_0443 | recA | CGTACATGTGTACT | -132 | 9.045632 | 0 | Y |
| 3 | sync_0036 | - | AGTACAAACGTATT | -59 | 8.6958 | 0 | Y |
| 4 | sync_0035 | wcaG-1 | AATACGTTTGTACT | -1 | 8.485786 | 5.88E-06 | Y |
| 5 | sync_1606 sync_1607 | umuD umuC | AGAACGTTTGTACT | -29 | 8.205994 | 0.000118 | Y |
| 6 | sync_1558 | - | AGTACGGTTGTTCT | -18 | 8.060134 | 0.000198 | Y |
| 7 | sync_2444 | - | AGTACAGCTGTACC | -128 | 7.927714 | 0.000327 | Y |
| 8 | sync_1344 | - | TGTACAGCTGTAGC | -592 | 7.879393 | 0.0004 | Y |
| 9 | sync_2443 | - | GGTACAGCTGTACT | -63 | 7.7522 | 0.000822 | N |
| 10 | sync_0748 sync_0749 sync_0750 sync_0751 | - - lpdA trpC | GGTACATGTGTCTT | -68 | 7.647649 | 0.001453 | Y |
| 11 | sync_0967 | petG | GGAACAATTGTGCT | -27 | 7.593391 | 0.00189 | Y |
| 12 | sync_1373 | - | TGTACACATGTAGT | -153 | 7.4272 | 0.004186 | N |
| 13 | sync_1474 | - | AGTTCAGACGTACT | -3 | 7.419285 | 0.00438 | Y |
| 14 | sync_0453 | cobO-1 | ATTACTGGTGTAAT | -355 | 7.374708 | 0.005159 | Y |
| 15 | sync_2248 sync_2247 sync_2246 sync_2245 sync_2244 | - - - oplaH - | GGTACGCCTGAACT | -165 | 7.37162 | 0.005159 | Y |
| 16 | sync_1473 | - | AGTACGTCTGAACT | -121 | 7.347081 | 0.005771 | Y |
| 17 | sync_0841 | - | TGTGCTGTTGTACT | -413 | 7.23908 | 0.008727 | Y |
| 18 | sync_1596 | - | TGCACGAATGTGCT | -106 | 7.232808 | 0.008727 | Y |
| 19 | sync_0129 | thiC | AGAACATGCGTACT | 7 | 7.210893 | 0.009447 | Y |

**Table S20**. Predicted LexA binding sites in *Synechococcus* CC9605 genome at *P* < 0.01

| **Rank** | **Transcription Unit** | **Name** | **Putative LexA Binding Site** | **Position** | **Score** | **p-value** | **Have an orthologue or not(Y/N)** |
| --- | --- | --- | --- | --- | --- | --- | --- |
| 1 | Syncc9605_1548 | - | AGTACTTCTGTACT | -12 | 9.561164 | 0 | Y |
| 2 | Syncc9605_0859 | - | AGTACAGATGTACT | -14 | 9.496293 | 0 | Y |
| 3 | Syncc9605_0381 | recA | GGTACGCTTGTACT | -66 | 9.051796 | 4.09E-05 | Y |
| 4 | Syncc9605_1180 | - | AGTACTCATGTACT | 5 | 8.91E+00 | 0.000155 | Y |
| 5 | Syncc9605_0036 | - | AATACAGGTGTACT | -1 | 8.861479 | 0.000196 | Y |
| 6 | Syncc9605_0858 Syncc9605_0857 Syncc9605_0856 | - - - | AGTACATCTGTACT | -82 | 8.821387 | 0.000216 | Y |
| 7 | Syncc9605_0475 Syncc9605_0476 Syncc9605_0477 Syncc9605_0478 | - - - - | GGTACACATGTACT | -69 | 8.678651 | 0.00028 | Y |
| 8 | Syncc9605_0774 | - | GGTACAGATGTACT | -13 | 8.614149 | 0.000325 | Y |
| 9 | Syncc9605_1179 Syncc9605_1178 | - - | AGTACATGAGTACT | -24 | 8.323897 | 0.000553 | Y |
| 10 | Syncc9605_1721 Syncc9605_1722 | - - | AGTGCATTTGTATT | -14 | 8.306877 | 0.000571 | Y |
| 11 | Syncc9605_1491 | - | AGTACAGATGTACT | -8 | 8.2205 | 0.000766 | N |
| 12 | Syncc9605_0550 | - | AGTACATCTGTATT | -19 | 7.8601 | 0.001598 | N |
| 13 | Syncc9605_1044 | - | GGTAGCTATGTACT | -50 | 7.787776 | 0.001999 | Y |
| 14 | Syncc9605_2670 | - | CGTACGGCTGTTTT | -3 | 7.761976 | 0.002087 | Y |
| 15 | Syncc9605_0551 Syncc9605_0552 | - - | AATACAGATGTACT | -131 | 7.4926 | 0.004541 | N |
| 16 | Syncc9605_2635 | - | CGGACACCTGTATT | -21 | 7.486971 | 0.004677 | Y |
| 17 | Syncc9605_0949 | - | AATACAGCTGTACT | -11 | 7.4015 | 0.005803 | N |
| 18 | Syncc9605_1794 | - | GGCACGCCTGTCCT | -111 | 7.341986 | 0.007124 | Y |

**Table S21**. Predicted LexA binding sites in *Synechococcus* CC9902 genome at *P* < 0.01

| **Rank** | **Transcription Unit** | **Name** | **Putative LexA Binding Site** | **Position** | **Score** | **p-value** | **Have an orthologue or not(Y/N)** |
| --- | --- | --- | --- | --- | --- | --- | --- |
| 1 | Syncc9902_0758 | - | AGTACAGATGTACT | -19 | 9.655024 | 0 | Y |
| 2 | Syncc9902_0711 | - | AGTACAGATGTACT | -14 | 9.527943 | 0 | Y |
| 3 | Syncc9902_1291 Syncc9902_1292 | - - | AGTACATTTGTACT | -15 | 9.15E+00 | 0 | Y |
| 4 | Syncc9902_1802 | - | AGTACAGATGTACT | -17 | 9.124677 | 0 | Y |
| 5 | Syncc9902_1949 | recA | CGTACGTTTGTACT | -64 | 8.993276 | 0 | Y |
| 6 | Syncc9902_1290 | - | AGTACAAATGTACT | -27 | 8.897006 | 0 | Y |
| 7 | Syncc9902_1803 | - | AGTACATCTGTACT | -126 | 8.701463 | 0.000199 | Y |
| 8 | Syncc9902_0032 Syncc9902_0031 | - - | AATACAGGTGTACT | -1 | 8.672836 | 0.00023 | Y |
| 9 | Syncc9902_0116 | - | AGTACATTTGTTTT | 26 | 8.113686 | 0.000282 | Y |
| 10 | Syncc9902_0847 Syncc9902_0848 Syncc9902_0849 Syncc9902_0850 | - - - - | GGTACCGGTGTTTT | -12 | 7.531238 | 0.003075 | Y |
| 11 | Syncc9902_1955 Syncc9902_1956 Syncc9902_1957 Syncc9902_1958 Syncc9902_1959 Syncc9902_1960 Syncc9902_1961 Syncc9902_1962 Syncc9902_1963 Syncc9902_1964 Syncc9902_1965 Syncc9902_1966 Syncc9902_1967 Syncc9902_1968 Syncc9902_1969 Syncc9902_1970 Syncc9902_1971 Syncc9902_1972 | - - - - - - rpsC - - - - - - - - - rpsE - | AGCACTTTTGTTCT | -51 | 7.516663 | 0.003476 | Y |
| 12 | Syncc9902_0894 Syncc9902_0895 Syncc9902_0896 | - - trpA | AGGACACCTGTCCT | -8 | 7.509744 | 0.003703 | Y |
| 13 | Syncc9902_2151 Syncc9902_2150 | - - | AGAACCCGTGTTCT | -24 | 7.50874 | 0.003703 | Y |
| 14 | Syncc9902_1455 Syncc9902_1456 Syncc9902_1457 | - - - | AATACCTTTGTCCT | -331 | 7.441328 | 0.004977 | Y |
| 15 | Syncc9902_0870 Syncc9902_0871 | - - | TGAACAGTTGTTCT | -149 | 7.400416 | 0.005629 | Y |
| 16 | Syncc9902_0230 Syncc9902_0231 Syncc9902_0232 | - - - | TGTTCTTATGTACT | -11 | 7.370642 | 0.006245 | Y |
| 17 | Syncc9902_0122 | - | AATACTCTTGTATT | -80 | 7.360948 | 0.006426 | Y |
| 18 | Syncc9902_0945 | - | GGAACATGTGTTCT | -298 | 7.323364 | 0.007197 | Y |
| 19 | Syncc9902_1000 | - | GTTACATATGTGCT | -236 | 7.319568 | 0.007417 | Y |
| 20 | Syncc9902_2181 | - | TCTACGGATGTTGT | 7 | 7.309789 | 0.007614 | Y |
| 21 | Syncc9902_0453 | - | GTTACGGCTGTCCT | -40 | 7.303827 | 0.007614 | Y |
| 22 | Syncc9902_1593 | - | ATTACAGCTGTGCT | -528 | 7.297911 | 0.007844 | Y |

**Table S22**. Predicted LexA binding sites in *Synechococcus* PCC7002 genome at *P* < 0.01

| **Rank** | **Transcription Unit** | **Name** | **Putative LexA Binding Site** | **Position** | **Score** | **p-value** | **Have an orthologue or not(Y/N)** |
| --- | --- | --- | --- | --- | --- | --- | --- |
| 1 | SYNPCC7002_A0426 | recA | AGTACGATTGAACT | -50 | 7.919635 | 0.00014 | Y |
| 2 | SYNPCC7002_A1285 | glnB | TGTACAATTGTCTT | -109 | 7.640727 | 0.000541 | Y |
| 3 | SYNPCC7002_A1374 SYNPCC7002_A1373 | - - | AGGACATTTGTAAT | 2 | 7.588826 | 0.000631 | Y |
| 4 | SYNPCC7002_A0582 | - | AGTCCCAATGTACT | -232 | 7.478054 | 0.00107 | Y |
| 5 | SYNPCC7002_A0191 | - | GATACGAATGTCCT | 35 | 7.441895 | 0.001202 | Y |
| 6 | SYNPCC7002_A1544 | - | GGTACAATTGCACT | -41 | 7.426008 | 0.001303 | Y |
| 7 | SYNPCC7002_A0814 | ndk | CGCACATTTGTAAT | 18 | 7.380815 | 0.001486 | Y |
| 8 | SYNPCC7002_A1674 SYNPCC7002_A1673 | - - | AGTGCTTTTGTACT | 20 | 7.377963 | 0.001546 | Y |
| 9 | SYNPCC7002_A0511 | hisC | AGCACCTATGTCCT | 7 | 7.347346 | 0.001817 | Y |
| 10 | SYNPCC7002_A1715 | galE | TGCACGAATGTTCT | -34 | 7.339756 | 0.001917 | Y |
| 11 | SYNPCC7002_A1845 SYNPCC7002_A1846 SYNPCC7002_A1847 | - gmk clpP | GGGACATCTGTATT | -402 | 7.296718 | 0.002421 | Y |
| 12 | SYNPCC7002_A0442 | - | TGTTCGGTTGTACT | -66 | 7.2524 | 0.002967 | Y |
| 13 | SYNPCC7002_A1268 | - | CGTACGTTTGTATG | 17 | 7.145497 | 0.005811 | Y |
| 14 | SYNPCC7002_A1008 | psaF | AGTACAAATGGATT | -57 | 7.130845 | 0.006149 | Y |
| 15 | SYNPCC7002_A1929 | apcB | ATTACTTCTGTAAT | 26 | 7.120107 | 0.006452 | Y |
| 16 | SYNPCC7002_A0659 | - | TGTACTAATGTTCA | -72 | 7.103227 | 0.007023 | Y |
| 17 | SYNPCC7002_A0448 | - | GGGACTGAGGTACT | -270 | 7.089055 | 0.007682 | Y |
| 18 | SYNPCC7002_A1214 | - | GGCACACCTGTACA | -140 | 7.080064 | 0.007682 | Y |
| 19 | SYNPCC7002_A2125 | glgA2 | AGTCCCTATGTACA | 5 | 7.055293 | 0.008699 | Y |
| 20 | SYNPCC7002_A1564 | - | ATTACTGTTGTGCT | -313 | 7.054988 | 0.008699 | Y |

**Table S23**. Predicted LexA binding sites in *Synechococcus* RCC307 genome at *P* < 0.01

| **Rank** | **Transcription Unit** | **Name** | **Putative LexA Binding Site** | **Position** | **Score** | **p-value** | **Have an orthologue or not(Y/N)** |
| --- | --- | --- | --- | --- | --- | --- | --- |
| 1 | SynRCC307_1534 | - | AGTACAGGTGTACT | -45 | 9.1934 | 0.0000000 | Y |
| 2 | SynRCC307_1535 SynRCC307_1536 SynRCC307_1537 | - - - | AGTACACCTGTACT | -73 | 8.9753 | 0.0007644 | Y |
| 3 | SynRCC307_1977 SynRCC307_1976 SynRCC307_1975 | - - - | GGTACGCCTGTACT | -66 | 8.4296 | 0.0010133 | Y |
| 4 | SynRCC307_1076 | - | AGTACGCATGTACT | -66 | 8.2650 | 0.0012691 | N |
| 5 | SynRCC307_0035 SynRCC307_0034 | wcaG - | GATACAGGTGTACT | -20 | 7.9868 | 0.0031257 | Y |
| 6 | SynRCC307_1756 | lexA | GGCACAAATGTATT | -66 | 7.8448 | 0.0035815 | Y |
| 7 | SynRCC307_0171 SynRCC307_0172 SynRCC307_0173 SynRCC307_0174 | - - - - | CTTACATCTGTAGT | -492 | 7.7632 | 0.0051724 | Y |
| 8 | SynRCC307_0579 SynRCC307_0580 SynRCC307_0581 | hisF - ubiE | AGCACTGGTGTGCT | -651 | 7.5570 | 0.0086986 | Y |

**Table S24**. Predicted LexA binding sites in *Synechococcus sp* WH8102 genome at *P* < 0.01

| Rank | Transcription Unit | Name | Putative LexA Binding Site | Position | Score | p-value | Have an orthologue or not(Y/N) |
| --- | --- | --- | --- | --- | --- | --- | --- |
| 1 | SYNW1138 | - | AGTACTGGTGTATT | -14 | 9.32066 | 0 | Y |
| 2 | SYNW2062 | recA | CGTACGCCTGTACT | -64 | 9.100352 | 2.57E-06 | Y |
| 3 | SYNW1466 | - | AGTACTGATGTACT | -1 | 9.007046 | 2.57E-06 | Y |
| 4 | SYNW1467 | - | AGTACATCAGTACT | -22 | 8.749925 | 2.31E-05 | Y |
| 5 | SYNW1045 | - | AGTACTGATGTATT | -54 | 8.65019 | 7.72E-05 | Y |
| 6 | SYNW0037 | - | GATACAGGTGTACT | -1 | 8.596895 | 0.000149 | Y |
| 7 | SYNW2107 SYNW2108 SYNW2109 SYNW2110 SYNW2111 | - - - - - | AGTACACCTGTTCT | -574 | 8.594202 | 0.000149 | Y |
| 8 | SYNW2106 | - | AGAACAGGTGTACT | -14 | 8.429305 | 0.000502 | Y |
| 9 | SYNW1140 | - | AGTACTTGCGTACT | -15 | 8.372088 | 0.000581 | Y |
| 10 | SYNW1661 | - | GGTACAGGTGTATT | -573 | 8.325865 | 0.000615 | Y |
| 11 | SYNW0347 | - | AGCACAATTGTCCT | -270 | 7.707843 | 0.003053 | Y |
| 12 | SYNW0684 | - | AGTTCTTTTGTACT | -240 | 7.572296 | 0.004236 | Y |
| 13 | SYNW0720 SYNW0719 SYNW0718 SYNW0717 SYNW0716 SYNW0715 SYNW0714 SYNW0713 SYNW0712 SYNW0711 | cytM - - - chlI ruvC - - - thrA | TGGACCTCTGTAGT | -568 | 7.563098 | 0.004311 | Y |
| 14 | SYNW1044 SYNW1043 | - umuC | AATACATCAGTACT | -25 | 7.552054 | 0.004414 | Y |
| 15 | SYNW1319 | - | CTTACAAATGTTCT | -78 | 7.515263 | 0.004846 | Y |
| 16 | SYNW1205 SYNW1204 SYNW1203 | - - - | AGCACCCCTGTTCT | -664 | 7.492278 | 0.005185 | Y |
| 17 | SYNW0873 SYNW0874 | - - | AAGACGGATGTACT | -133 | 7.476718 | 0.005435 | Y |
| 18 | SYNW0450 | - | CGTACAGAAGTAAT | -55 | 7.446695 | 0.006044 | Y |
| 19 | SYNW0959 SYNW0958 | - - | TATACCTATGTGCT | -25 | 7.438364 | 0.006255 | Y |
| 20 | SYNW2410 | - | TGTACAATTGTATC | -16 | 7.402841 | 0.007063 | Y |
| 21 | SYNW1148 SYNW1149 | - prfC | AGCACAGATGTTGT | -94 | 7.368741 | 0.008336 | Y |
| 22 | SYNW2347 SYNW2346 SYNW2345 SYNW2344 SYNW2343 SYNW2342 | gloA clpB2 secE nusG rpl11 rpl1 | GGTACAGGGGTAGT | -503 | 7.352108 | 0.008686 | Y |

**Table S25**. Predicted LexA binding sites in *Synechococcus sp* WH7803 genome at *P* < 0.01

| **Rank** | **Transcription Unit** | **Name** | **Putative LexA Binding Site** | **Position** | **Score** | **p-value** | **Have an orthologue or not(Y/N)** |
| --- | --- | --- | --- | --- | --- | --- | --- |
| 1 | SynWH7803_0843 | - | AGTACAGATGTACT | -13 | 9.8770 | 0 | Y |
| 2 | SynWH7803_1500 | - | AGTACATCTGTACT | -8 | 9.5961 | 0 | Y |
| 3 | SynWH7803_0439 | recA | CGTACATCTGTACT | -132 | 9.3132 | 0 | Y |
| 4 | SynWH7803_0037 | - | AGTACAAACGTATT | -59 | 8.9275 | 7.62E-05 | Y |
| 5 | SynWH7803_0036 | wcaG | AATACGTTTGTACT | -1 | 8.8408 | 8.13E-05 | Y |
| 6 | SynWH7803_0844 | - | AGTACATCTGTACT | -29 | 8.6129 | 0.000119 | Y |
| 7 | SynWH7803_0508 | - | AGTACCGCTGTTGT | -128 | 8.2003 | 0.000353 | Y |
| 8 | SynWH7803_0523 SynWH7803_0524 SynWH7803_0525 SynWH7803_0526 | smf hemK - - | AATACATGTGTACT | 5 | 8.1804 | 0.000384 | Y |
| 9 | SynWH7803_0815 SynWH7803_0816 SynWH7803_0817 | - - - | GGTACGCCTGTTCT | -89 | 8.0009 | 0.000996 | Y |
| 10 | SynWH7803_1595 | petG | GGAACAATTGTGCT | -25 | 7.6932 | 0.002243 | Y |
| 11 | SynWH7803_1534 | - | AGTGCGGATGTACT | -544 | 7.6141 | 0.003186 | Y |
| 12 | SynWH7803_2160 | - | ACTACACGTGTGTT | -46 | 7.4489 | 0.006728 | Y |
| 13 | SynWH7803_2033 SynWH7803_2034 SynWH7803_2035 | - - glnB | GGTACAACTGCATT | -148 | 7.4438 | 0.006728 | Y |
| 14 | SynWH7803_2039 | fumC | GGCACCCATGTTCT | -93 | 7.4002 | 0.007663 | Y |
| 15 | SynWH7803_2314 SynWH7803_2315 | ndhH - | AGCACGAATGTTTT | -13 | 7.3898 | 0.008166 | Y |
| 16 | SynWH7803_0568 SynWH7803_0569 SynWH7803_0570 SynWH7803_0571 SynWH7803_0572 SynWH7803_0573 | engA cbiQ - - - proC | CGTGCCTCTGTACT | -30 | 7.3678 | 0.008732 | Y |

**Table S26**. Predicted LexA binding sites in *Synechocystis* PCC6803 genome at *P* < 0.01

| Rank | Transcription Unit | Name | Putative LexA Binding Site | Position | Score | p-value | Have an orthologue or not(Y/N) |
| --- | --- | --- | --- | --- | --- | --- | --- |
| 1 | slr0616 | - | AGCACTATTGTACT | -258 | 7.904186 | 2.50E-05 | Y |
| 2 | slr0070 slr0467 slr0468 | fmt natA - | ACTACGACTGTACT | -162 | 7.477537 | 0.000884 | Y |
| 3 | slr2030 | - | TGTACAGATGTTTT | -541 | 7.468228 | 0.000907 | Y |
| 4 | sll0267 sll0268 | - - | AGTACTATTGTTCG | -50 | 7.43E+00 | 0.00105 | Y |
| 5 | sll0223 ssl0410 | ndhB - | TGTACCTATGGACT | 7 | 7.35E+00 | 0.001501 | Y |
| 6 | sll1770 sll1771 sll1772 | - - mutS | CGCACCTATGTAAT | -108 | 7.316434 | 0.0019 | Y |
| 7 | sll0041 sll0042 sll0043 sll0044 | - tar - - | GGTACTGATGTCCA | -40 | 7.278092 | 0.002352 | Y |
| 8 | sll0051 | - | AGTACAGTCGTAGT | -39 | 7.26331 | 0.002471 | Y |
| 9 | sll0921 | - | CGTACTATTGTTCC | -264 | 7.243182 | 0.002684 | Y |
| 10 | sll0300 | ribC | GGCACAATTGTAAT | -24 | 7.22139 | 0.002928 | Y |
| 11 | slr0906 | psbB | CATACAGTTGTCCT | 36 | 7.182166 | 0.003615 | Y |
| 12 | sll0514 | - | AGTACGACGGTAAT | -76 | 7.167144 | 0.00401 | Y |
| 13 | sll0644 | - | TGGACCATTGTACA | -147 | 7.162814 | 0.00401 | Y |
| 14 | sll1832 sll1833 | - ftsI | CGCACCTTTGTAGT | -16 | 7.09775 | 0.006076 | Y |
| 15 | slr2031 | - | AGAACAATTGTTTT | -114 | 7.094114 | 0.006076 | Y |
| 16 | slr2095 slr2096 slr2097 | - - glbN | GGGACTTATGTTTT | -170 | 7.05515 | 0.007526 | Y |
| 17 | sll0622 sll0623 | nadA - | AGCACCCCTGTGTT | -339 | 7.039534 | 0.00827 | Y |
| 18 | slr0239 | cbiF | GGTACTAGAGTATT | -186 | 7.008976 | 0.009391 | Y |
| 19 | sll1910 | zam | TGTACACCTGCAGT | -120 | 7.000474 | 0.009391 | Y |
