# Supplementary material for: Computational analysis of LexA regulons in Cyanobacteria
Source: BMC Genomics. 2010 Sep 29;11:527. doi: 10.1186/1471-2164-11-527 (PMC3091678; doi:10.1186/1471-2164-11-527)
Supplement: Additional file 5 — Supplementary tables. Additional file 5 contains 5 tables: Table S27-31 containing the predicted LexA-binding sites in the five cyanobacterial genomes without a lexA gene at p < 0.01. [file 1471-2164-11-527-S5.DOC]

**Table S27**. Predicted LexA binding sites in *Synechococcus sp._JA-3-3Ab_ A-Prime* at *P* < 0.01

| **Rank** | **Transcription Unit** | **Name** | **Putative lexA Binding Site** | **Position** | **Score** | **p-value** | **Have an orthologue or not(Y/N)** |
| --- | --- | --- | --- | --- | --- | --- | --- |
| 1 | CYA_0085 CYA_0084 | - rpe | CGTACCTTTGTCCT | -28 | 8.476996 | 6.64E-06 | Y |
| 2 | CYA_0538 CYA_0539 | - - | AGTACAAATGTAAA | -27 | 8.470296 | 6.64E-06 | Y |
| 3 | CYA_1866 CYA_1867 CYA_1868 | aroB asd - | TGTACAACTGTGCT | -320 | 8.112141 | 0.000164 | Y |
| 4 | CYA_2601 CYA_2600 CYA_2599 | - psbX-2 - | CGCACATCTGTATT | -79 | 8.033193 | 0.000343 | Y |
| 5 | CYA_0537 | - | TTTACATTTGTACT | -22 | 8.00421 | 0.000412 | Y |
| 6 | CYA_0493 | - | TGTACTGTAGTCCT | -139 | 7.991888 | 0.000465 | Y |
| 7 | CYA_0829 CYA_0828 | gvpA - | TCTACGGGTGTTCT | -154 | 7.797256 | 0.001308 | Y |
| 8 | CYA_0687 CYA_0688 CYA_0689 | - - - | CATACGTTTGTGGT | -480 | 7.753525 | 0.001565 | Y |
| 9 | CYA_0841 | - | ACTACAACTGTACA | -269 | 7.738039 | 0.001729 | Y |
| 10 | CYA_0149 CYA_0150 CYA_0151 CYA_0152 CYA_0153 CYA_0154 CYA_0155 | - - - - - - - | AGTAACTGTGTGGT | 29 | 7.732825 | 0.001729 | Y |
| 11 | CYA_2257 | rbsK | AGCACGTCTGTAGT | -132 | 7.722471 | 0.001729 | Y |
| 12 | CYA_0541 CYA_0542 | - glpX | AGTACAAATGTAAA | -623 | 7.686817 | 0.002078 | Y |
| 13 | CYA_0081 | miaA | ACTACGGATGTTCT | -71 | 7.567442 | 0.003316 | Y |
| 14 | CYA_2528 CYA_2530 | - - | GGAACTTCTGTTCT | -169 | 7.485461 | 0.004427 | Y |
| 15 | CYA_0598 | - | AGCACGAATGTGCT | -339 | 7.409482 | 0.005748 | Y |
| 16 | CYA_0933 | - | AGAACTTTTGTCCT | -252 | 7.404814 | 0.005748 | Y |
| 17 | CYA_1541 | - | GGAACACTTGTCCT | -96 | 7.400937 | 0.005748 | Y |
| 18 | CYA_2302 | scpA | GCTACCACTGTGCT | -185 | 7.397565 | 0.006158 | Y |
| 19 | CYA_2369 CYA_2370 CYA_2371 | trpC - - | AGCACCTTTGTCTT | -205 | 7.390843 | 0.006158 | Y |
| 20 | CYA_2848 | scpB | TGTGCAATTGTACT | -151 | 7.36351 | 0.006549 | Y |
| 21 | CYA_1009 CYA_1008 | - - | CGTACCCATTTGCT | -235 | 7.343591 | 0.007025 | Y |
| 22 | CYA_1518 | - | AGCACAGTTGTACA | -566 | 7.327316 | 0.00755 | Y |
| 23 | CYA_2210 | - | GCTACTTTTGTTCA | 18 | 7.291304 | 0.008752 | Y |
| 24 | CYA_2425 CYA_2424 CYA_2423 CYA_2422 CYA_2421 CYA_2420 CYA_2419 | - - - - pyrH - yqeG | AGAAGCTCTGTACT | -160 | 7.282493 | 0.008752 | Y |
| 25 | CYA_1135 CYA_1134 CYA_1133 | rpmF - - | AGTAAAGCTGTGTT | -12 | 7.270576 | 0.009318 | Y |
| 26 | CYA_0574 CYA_0575 CYA_0577 | - speE - | GGTAAAGCTGTGCT | -50 | 7.269388 | 0.009318 | Y |

**Table S28**. Predicted LexA binding sites in *Synechococcus_sp_ JA-2-3B'a_(2-13)_B-Prime* at *P* < 0.01

| **Rank** | **Transcription Unit** | **Name** | **Putative lexA Binding Site** | **Position** | **Score** | **p-value** | **Have an orthologue or not(Y/N)** |
| --- | --- | --- | --- | --- | --- | --- | --- |
| 1 | CYB_0550 | - | TGTACAGTAGTCCT | -52 | 8.061455 | 6.60E-04 | Y |
| 2 | CYB_0281 CYB_0282 | psbB psbT | CATACGGTTGTACT | 36 | 7.92635 | 8.28E-04 | Y |
| 3 | CYB_2048 | - | CCTACTCTTGTTCT | -17 | 7.914811 | 0.000875 | Y |
| 4 | CYB_1253 CYB_1254 CYB_1255 CYB_1256 CYB_1257 | gltS acsF - - - | TGTACATTTGTACA | -360 | 7.809637 | 0.001282 | Y |
| 5 | CYB_1833 | gvpA | TCTACGGGTGTTCT | -169 | 7.749744 | 0.001583 | Y |
| 6 | CYB_2126 CYB_2127 CYB_2128 | - - - | CATACGTTTGTGGT | -482 | 7.7056 | 0.001863 | Y |
| 7 | CYB_1353 | rbsK | AGCACGTCTGTAGT | -425 | 7.677726 | 0.002195 | Y |
| 8 | CYB_1885 CYB_1884 CYB_1883 CYB_1882 CYB_1881 CYB_1880 CYB_1879 CYB_1878 CYB_1877 CYB_1876 | - - - - - - - aspC - - | AGTAACTGTGTGGT | 31 | 7.676425 | 0.002195 | Y |
| 9 | CYB_0911 | cphA | TGTACCGATGTGGT | -217 | 7.634317 | 0.002555 | Y |
| 10 | CYB_2255 | - | AGTGCAAATGTAAA | -130 | 7.590563 | 0.002906 | Y |
| 11 | CYB_0803 | phrB-2 | TGTACTGCTGTGGT | 19 | 7.567253 | 0.003107 | Y |
| 12 | CYB_2295 | - | TGGACAACTGTAAT | -410 | 7.548729 | 0.003296 | Y |
| 13 | CYB_0813 CYB_0812 | - psbX | TACACGTCTGTACT | -80 | 7.535736 | 0.003476 | Y |
| 14 | CYB_1328 CYB_1329 CYB_1330 CYB_1331 CYB_1332 CYB_1333 CYB_1334 CYB_1335 CYB_1336 CYB_1337 CYB_1338 CYB_1339 | - - - - - - - ligA clpX - - - | CGGACAGCTGTCCT | -119 | 7.449692 | 0.004302 | Y |
| 15 | CYB_2552 | - | AGTACCCATGTATC | -129 | 7.44281 | 0.004302 | Y |
| 16 | CYB_0283 | - | AGAACGCTTGTCCT | -97 | 7.413728 | 0.004928 | Y |
| 17 | CYB_1816 | - | AGTAAGGATGTTCT | -100 | 7.370576 | 0.005648 | Y |
| 18 | CYB_1154 | scpA | GCTACCACTGTGCT | -164 | 7.352236 | 0.00608 | Y |
| 19 | CYB_1372 CYB_1371 | psbN dapF | TGTAATACTGTAGT | -29 | 7.282014 | 0.0076 | Y |
| 20 | CYB_1245 CYB_1244 CYB_1243 CYB_1242 CYB_1241 | psaE mutM - - - | CGGACTAGGGTAAT | -29 | 7.270525 | 0.008179 | Y |
| 21 | CYB_1048 | - | GCTACCTTTGTTCA | 18 | 7.242314 | 0.008776 | Y |
| 22 | CYB_0794 CYB_0793 CYB_0792 CYB_0791 CYB_0790 CYB_0789 CYB_0788 CYB_0787 CYB_0786 CYB_0785 | - - - - - - - - - - | AGGACTGATGTTTT | -82 | 7.239649 | 0.009382 | Y |
| 23 | CYB_2790 | - | TGTAAGACTGTACA | -282 | 7.227932 | 0.009382 | Y |
| 24 | CYB_0472 CYB_0473 CYB_0474 | - - - | CGTACCTGTCTGCT | -80 | 7.227234 | 0.009382 | Y |
| 25 | CYB_0855 CYB_0856 CYB_0857 CYB_0858 CYB_0859 | petM panE-1 - - - | AGAACGACTGTTTT | -107 | 7.208434 | 0.009938 | Y |
| 26 | CYB_1940 | - | AGTAAACCTGTTCT | -13 | 7.202243 | 0.009938 | Y |

**Table S29**. Predicted LexA binding sites in *Synechococcus_elongatus_PCC_6301* at *P* < 0.01

| **Rank** | **Transcription Unit** | **Name** | **Putative lexA Binding Site** | **Position** | **Score** | **p-value** | **Have an orthologue or not(Y/N)** |
| --- | --- | --- | --- | --- | --- | --- | --- |
| 1 | syc1636_c | dnaK | AGCACATCTGTATT | -493 | 7.410404 | 6.59E-04 | Y |
| 2 | syc1953_c | - | TTTACCGTTGTACT | -627 | 7.384001 | 7.58E-04 | Y |
| 3 | syc1661_d | sphX | CGTGCTGCTGTACT | 39 | 7.277322 | 0.00169 | Y |
| 4 | syc0443_d | - | CGTACCCTTGTTTT | 18 | 7.241084 | 0.0019 | Y |
| 5 | syc1187_d syc1188_d | apcB apcC | ATTACCGCTGTCAT | 24 | 7.236871 | 0.002106 | Y |
| 6 | syc0225_c syc0224_c | - - | TGTGCTTCTGTACT | -30 | 7.2273 | 0.002106 | Y |
| 7 | syc1626_d syc1627_d | psb28 - | AGGACTGGTGTAGT | -316 | 7.193185 | 0.002593 | Y |
| 8 | syc0472_c | - | CGCACAGCTGTCCT | -59 | 7.167266 | 0.002824 | Y |
| 9 | syc0254_d | - | TCTACTTATGTTCT | -135 | 7.139521 | 0.003306 | Y |
| 10 | syc0253_c syc0252_c | - recQ | AGTGCAAATGTCCT | -64 | 7.078275 | 0.004221 | Y |
| 11 | syc1761_d | psaL | TGCACTATTGTCCT | -169 | 6.998538 | 0.005905 | Y |
| 12 | syc0662_c syc0661_c | rne rnhB | GGTAGGGATGTCCT | -22 | 6.964217 | 0.006465 | Y |
| 13 | syc1050_d syc1051_d | secG - | GTTACTTCTGTTTT | -9 | 6.884552 | 0.008819 | Y |

**Table S30**. Predicted LexA binding sites in *Thermosynechococcus_elongatus_BP-1* at *P* < 0.01

| **Rank** | **Transcription Unit** | **Name** | **Putative lexA Binding Site** | **Position** | **Score** | **p-value** | **Have an orthologue or not(Y/N)** |
| --- | --- | --- | --- | --- | --- | --- | --- |
| 1 | tll2476 tll2475 | - trpB | TGTACCACTGTATT | -52 | 7.641883 | 1.84E-03 | Y |
| 2 | tll0418 | - | AGTACACCTGTACC | -459 | 7.517722 | 2.28E-03 | Y |
| 3 | tll1562 | ycf21 | ACTACGACTGTACT | -163 | 7.488005 | 0.002437 | Y |
| 4 | tll0139 tll0138 | - - | TGTTCTATTGTACT | -67 | 7.244651 | 0.005005 | Y |
| 5 | tlr1669 | - | TTTACCCCTGTGCT | -182 | 7.173914 | 0.006341 | Y |
| 6 | tlr1343 tlr1344 | - - | GGTACGCTTGTACG | -434 | 7.169057 | 0.006341 | Y |
| 7 | tll0278 | - | AATACCCATGTCCT | 7 | 7.136161 | 0.007048 | Y |
| 8 | tlr0516 | - | AGAACGCTTGTAGT | -310 | 7.129345 | 0.007048 | Y |
| 9 | tll0956 | apcB | ATTACCGCTGTCAT | 26 | 7.110027 | 0.007439 | Y |
| 10 | tll1488 tll1487 tll1486 | - - - | TGTACCACTGTCCA | -738 | 7.073086 | 0.008198 | Y |
| 11 | tlr1794 tlr1795 | - - | TTTACAGTTGTCCT | -229 | 7.068875 | 0.008198 | Y |

**Table S31**. Predicted LexA binding sites in *Trichodesmium_erythraeum_IMS101* at *P* < 0.01

| **Rank** | **Transcription Unit** | **Name** | **Putative LexA Binding Site** | **Position** | **Score** | **p-value** | **Have an orthologue or not(Y/N)** |
| --- | --- | --- | --- | --- | --- | --- | --- |
| 1 | Tery_1467 | - | GGTACAGTTGTACT | -125 | 8.532914 | 1.02E-06 | Y |
| 2 | Tery_4594 | - | AGAACAATTGTACT | 27 | 8.121846 | 0.0001 | Y |
| 3 | Tery_4681 | - | TGTACGGCTGTATT | -517 | 7.954467 | 0.000263 | Y |
| 4 | Tery_0893 Tery_0894 | - - | TGTACCACTGTACC | -657 | 7.935048 | 0.000302 | Y |
| 5 | Tery_2940 | - | AGCACATCTGTTCT | -180 | 7.896237 | 0.000416 | Y |
| 6 | Tery_0682 Tery_0683 | - - | AGTGCGACTGTACT | -545 | 7.879762 | 0.00049 | Y |
| 7 | Tery_4713 Tery_4714 | - - | TGTACAAATGTTTT | -170 | 7.729046 | 0.001107 | Y |
| 8 | Tery_2189 | - | GGTACTTTTGTAGT | -292 | 7.709084 | 0.001181 | Y |
| 9 | Tery_0046 Tery_0047 | - - | AGTACGGATGTTCT | -271 | 7.6609 | 0.00143 | N |
| 10 | Tery_2339 | - | TGTACTAATGTACC | -190 | 7.657754 | 0.001564 | Y |
| 11 | Tery_0376 | - | AGAACTTATGTACC | -151 | 7.608025 | 0.00189 | Y |
| 12 | Tery_1057 | - | TGTACTTAGGTCCT | -445 | 7.578124 | 0.002267 | Y |
| 13 | Tery_1970 | - | AATACAGATGTCCT | -747 | 7.511813 | 0.00301 | Y |
| 14 | Tery_2380 | - | AGTACCATTGTATC | -217 | 7.490494 | 0.003362 | Y |
| 15 | Tery_0847 | - | GGTACACCAGTACC | -224 | 7.470958 | 0.003721 | Y |
| 16 | Tery_0356 | - | GGTACTTTTGTAAA | -698 | 7.456603 | 0.004119 | Y |
| 17 | Tery_2140 | - | GGTTCTGCTGTTCT | -43 | 7.433517 | 0.004558 | Y |
| 18 | Tery_4312 | - | AGTACTTGAGTAAT | -380 | 7.40385 | 0.004996 | Y |
| 19 | Tery_3195 | - | TGCACTTATGTAAT | -669 | 7.393631 | 0.005484 | Y |
| 20 | Tery_2630 | - | AGCACCATTGTAAT | -484 | 7.377868 | 0.006022 | Y |
| 21 | Tery_2818 Tery_2817 | - - | CCTACTCTTGTACT | -345 | 7.345866 | 0.006616 | Y |
| 22 | Tery_3401 Tery_3402 | - - | AGTACCACCGTATT | -603 | 7.333982 | 0.007156 | Y |
| 23 | Tery_1635 | - | CATACTTTTGTACT | -293 | 7.282384 | 0.008479 | Y |
| 24 | Tery_2667 | - | AGTACCTGAGTAGT | -784 | 7.262245 | 0.009121 | Y |
| 25 | Tery_2009 | - | GGTCCAAATGTACG | -279 | 7.245904 | 0.009742 | Y |
